# Proviral HIV-genome-wide and pol-gene specific Zinc Finger Nucleases: Usability for targeted HIV gene therapy

**DOI:** 10.1186/1742-4682-8-26

**Published:** 2011-07-22

**Authors:** Misaki Wayengera

**Affiliations:** 1 Unit of Genetics, Genomics & Theoretical Biology, Dept of Pathology, School of Biomedical Science, College of Health Sciences, Makerere University. P O Box 7072 Kampala, Uganda

## Abstract

**Background:**

Infection with HIV, which culminates in the establishment of a latent proviral reservoir, presents formidable challenges for ultimate cure. Building on the hypothesis that *ex-vivo *or even *in-vivo *abolition *or *disruption of HIV-gene/genome-action by target mutagenesis or excision can irreversibly abrogate HIV's innate fitness to replicate and survive, we previously identified the isoschizomeric bacteria restriction enzymes (REases) AcsI and ApoI as potent cleavers of the HIV-pol gene (11 and 9 times in HIV-1 and 2, respectively). However, both enzymes, along with others found to cleave across the entire HIV-1 genome, slice (SX) at palindromic sequences that are prevalent within the human genome and thereby pose the risk of host genome toxicity. A long-term goal in the field of R-M enzymatic therapeutics has thus been to generate synthetic restriction endonucleases with longer recognition sites limited in specificity to HIV. We aimed (i) to assemble and construct zinc finger *arrays *and *nucleases *(ZFN) with either proviral-HIV-pol gene or proviral-HIV-1 whole-genome specificity respectively, and (ii) to advance a model for pre-clinically testing lentiviral vectors (LV) that deliver and transduce either ZFN genotype.

**Methods and Results:**

*First, *we computationally generated the consensus sequences of (a) 114 dsDNA-binding zinc finger (Zif) *arrays *(ZFAs or Zif_HIV-pol_) and (b) two zinc-finger *nucleases *(ZFNs) which, unlike the AcsI and ApoI homeodomains, possess specificity to >18 base-pair sequences uniquely present within the HIV-pol gene (Zif_HIV-pol_F_N_). Another 15 ZFNs targeting >18 bp sequences within the complete HIV-1 proviral genome were constructed (Zif_HIV-1_F_N_). *Second, *a model for constructing lentiviral vectors (LVs) that deliver and transduce a diploid copy of either Zif_HIV-pol_F_N _or Zif_HIV-1_F_N _chimeric genes (termed **LV- 2xZif**_**HIV-pol**_**F**_**N **_and **LV- 2xZif**_**HIV-1**_**F**_**N, **_respectively) is proposed. *Third, *two preclinical models for controlled testing of the safety and efficacy of either of these LVs are described using active HIV-infected TZM-bl reporter cells (HeLa-derived JC53-BL cells) and latent HIV-infected cell lines.

**Conclusion:**

**LV-2xZif**_**HIV-pol**_**F**_**N **_and **LV- 2xZif**_**HIV-1**_**F**_**N **_may offer the *ex-vivo *or even *in-vivo *experimental opportunity to halt HIV replication functionally by directly abrogating HIV-pol-gene-action *or *disrupting/excising over 80% of the proviral HIV DNA from latently infected cells.

## Background

### -The global challenge of human immunodeficiency virus (HIV) infection

Human infection with the retrovirus-human immunodeficiency virus (HIV) causes acquired immunodeficiency syndrome (AIDS) [1]. Over a quarter a century since the description of the first clinical cases of AIDS, HIV/AIDS remains a global health challenge [2,3]. There are now over 33 million people currently infected with HIV world-over, and 25 million lives have already been lost to AIDS. Despite the advent of a powerful regimen of highly active anti-retroviral therapy (HAART) to treat HIV/AIDS, HAART has its limitations [4,5]. Specifically, while HAART targets actively replicating HIV, latent-HIV infection, particularly proviral HIV DNA integrated with resting CD4+ve cells, ultimately acts as a source of rebound viremia once treatment is stopped. Recent reports suggest that the reservoir of latent proviral HIV infection may extend beyond just the experimentally demonstrated CD4+ resting memory cells to include cells of the macrophage, natural-killer, dendrite, astrocyte and bone marrow progenitor lineages [6,7]. Overall, in the absence of a vaccine that is 100% effective, novel strategies to tackle the unique challenge of latent HIV infection among patients on HAART are urgently sought [7]. Although different mechanisms for the maintenance of reservoirs of latent HIV-infection have been advanced, the spectrum of emerging trial anti-HIV latency 'pro-drugs' is largely limited to those agents functioning via the awakening of resting host (CD4+ memory) cells; a strategy primarily meant to exorcise the latent provirus [5,6]. Specifically, most of the trial anti-HIV latency pro-drugs (operating by non-specific stimulation of T cell receptors, TCR) function *either *globally via nuclear factor of activated T cells (NFAT) and protein C-kinase (PCK), *or *specifically via reductive oxidative substrates (ROS) and cytokines such as tumor necrotic factor-alpha (TNF-α) and interleukin-7 [8-11].

### -The alternative option of directly disrupting or abolishing HIV gene expression

In 1999, I [12] first proposed the possibility of using the anti-phage DNA machinery inherent in *bacteria *-- the restriction modification (R-M) system (itself a primitive anti-viral immunity) - as a model for devising eukaryotic virus gene therapies. Over the past 10 years, I and colleagues [13,14] have identified several bacterially-derived restriction enzymes with potential to cleave the DNA of human-infecting viruses, including frequency and site mapping of HIV-1, HIV-2 and several other SIV gene-cleavage using a proviral DNA model [15]. The isoschizomeric bacterial restriction enzymes (REases) AcsI and ApoI have, for instance, specifically been found to possess high potency to cleave (slice or disrupt) the HIV pol gene (11 and 9 times in HIV-1 and -2, respectively) [15]. Both enzymes, along with their third isoschizomer XapI, cleave at the palindromic site defined by the sequences 5'-RAATY-3'. Given the high incidence within the human host genome of site-specific units (palindromes) similar to those of the REases identified, matters of *in-situ *safety have proven a priority that is difficult to address, limiting our prior attack-models to the extracellular space [14,16-21]. Specifically, because of the smaller sizes of phage genomes, bacteria evolve to select for R-M systems with small recognition sites (4-6 bp), since these sites occur more frequently in phages. However, this feature - a high incidence of palindromes - also renders the human genome highly susceptible to REase-activity. Therefore, a long-term goal in the field of R-M enzymatic therapeutics has been to generate synthetic restriction endonucleases with longer recognition sites specific only to the eukaryotic virus, by mutating or engineering existing enzymes.

### Zinc Finger Nuclease technology and its applicability in antiviral gene therapy development

Zinc finger *nucleases *- ZFNs - which are artificial, hybrid restriction enzymes created by covalently linking a DNA-binding zinc finger (Zif) domain (composed of three to six finger-arrays) to the non-specific DNA cleavage domain (or simply F_N_) of the Flavobacterium *Okeanokoites *bacterial restriction endonuclease-FokI, have recently become a powerful tool for either primarily editing host genomes to halt viral infectivity, or secondarily targeting incoming or established viral genomes [22-30]. On the one hand, Perez *et al*. [27], using engineered ZFNs targeting human CCR5, previously demonstrated the establishment of HIV-1 resistance in CD4+ T cells through generation of a double-strand break (DSB) at predetermined sites in the CCR5 coding region upstream of the natural CCR5D32 mutation. More recently, Holmes *et al*. [28] demonstrated control of HIV-1 infection within NSG mice transplanted with human hematopoietic stem/progenitor cells modified by zinc-finger nucleases targeting CCR5. On the other hand, with the intent of disrupting incoming viral genomes, Gross *et al*. [29], have recently demonstrated homing (mega-) endonuclease-mediated inhibition of HSV-1 infection in cultured cells. Indeed, Cradick *et al. *[30] had previously shown that zinc finger nucleases could equally offer a novel therapeutic strategy for targeting Hepatitis B Virus DNAs.

On the basis of the above advances in the field of ZFN technology, which permit the generation of synthetic restriction enzymes that are expressible within the human genome without causing functional or structural-genome toxicity, we postulated that synthetic zinc finger *nucleases *(ZFNs) with specificity to > 18 bp- palindromic sequence within the HIV-pol gene, unlike the 5'-RAATY-3' five-bp targeted by AcsI and ApoI, can specifically disrupt the HIV-pol gene with no toxicity-risk to the human genome[25-30]. Therapeutically, observing that the HIV-pol gene (~3,182 base pairs), which codes for the enzymes reverse transcriptase (RT), integrase and protease, is an indispensable section of the HIV genome for viral replication and survival, *ex-vivo *or even *in-vivo *disruption or abolition of HIV-pol should result in irreversible abrogation of HIV's innate fitness to replicate and survive [1]. Alternatively, however, one may opt to target the entirety or most of the proviral genome for either disruption or excision. While inhibition of HIV replication *in-vivo *using small artificial molecules modified to harness the target DNA-binding mechanism inherent in zinc finger (ZF) domains as a strategy to repress HIV transcription has previously been reported by Segal *et al. *[31] and Eberhardy *et al*. [32], respectively, ZFN-based disruption or abolition of HIV genes has yet to be reported. *In other words, this work, unlike previous ZFN-based strategies aiming to cure HIV by targeting the host pathways, is an attempt to attack and modify the HIV pathway directly using ZFN technology.*

The goal of this work was to identify and engineer, respectively, (i) HIV-pol gene and HIV-1 whole genome specific ZF *arrays *(ZFAs) and (ii) ZF-*nucleases *(ZFNs); as well as model construction and pre-clinical testing of lentiviral vectors (LVs) that deliver and transduce a diploid copy of either HIV-specific ZFN genotype.

## Methods, results and discussion

### Assembly of HIV-pol gene/HIV-1-proviral -dsDNA binding zinc finger *arrays *and construct of HIV-pol gene/HIV-1-proviral-dsDNA cleaving zinc finger *nucleases*

**Figure 1:**
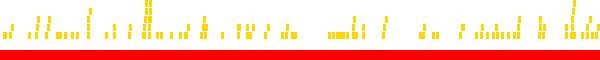
**A graphic map of the distribution of the recognized target DNA sites by the 114 multi-Zif-arrays, along the entire length of the SIV/HIV-pol-gene**. The figure offers a detailed graphics illustration of the distribution of target DNA sites along the full 3,182 bp lengths of the SIV/HIV-pol gene recognized by all 114 multi-Zif-arrays. For details, see Table 1 and Additional file 1.

**Figure 2:**
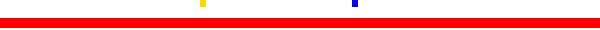
**A graphic map of the distribution of the target-DNA sites recognized by the two ZFNs obtained along the entire length of the SIV/HIV-pol-gene**. This figure offers a graphic map of the distribution of target DNA sites recognized and cleaved by our two ZFNs, along the full 3,182 bp lengths of the SIV/HIV-pol gene. For details, see Table 2 and Additional file 2.

**Figure 3:**
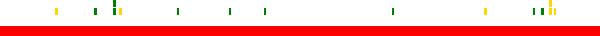
**A graphic map of the distribution of the target-DNA sites recognized by 15 ZFNs obtained along the entire length of the HIV-1 genome**. This figure offers a graphic map of distribution of target DNA sites recognized and cleaved by the 15 ZFNs along the full 9,182 bp lengths of the HIV-1 genome. For details, see additional file 3.

*First, *using the Zinc Finger Consortium's software ZiFiT-CoDA-ZFA and the complete FASTA sequences of the SIV/HIV-pol gene [Genbank: NC_001870.1 > gi|9629914:1714-4893], we assembled 114 ZFA with unique specificity to 9 bp sequences within the SIV/HIV-pol gene. The ZiFit software operates on algorithms primarily build by researchers from the Barbas lab [33,34] with minimal modifications [33-36]. Throughout our computational context-dependent assembly (CoDA) experiments, the ZiFiT software was set at default setting and the exon/intron case-sensitivity algorithm turned to its ON-mode, thereby allowing us to distinguish between intron and exon sequences by denoting exons as uppercase and introns as lowercase [36]. These 114 SIV/HIV-pol gene specific ZFAs comprise three zinc finger (ZF) proteins linked together. Overall, each ZF is a protein motif that has two beta strands and an alpha helix [23-26]. The beta strands and alpha helix are stabilized by coordination of a zinc ion mediated by pairs of conserved cysteine and histidine residues. Residues 1 to 6 of the alpha-helix (numbered relative to the start of the helix) are responsible for the specific recognition of triplets of DNA sequences through the formation of base-specific contacts in the major groove of the double-stranded target DNA [37-41]. Thus, residues 1 to 6 within ZF alpha helices are denoted 'recognition' residues, and these are listed in N- to C-terminal direction, while all other residues in the ZF are called the 'backbone'. ZFs bind target DNA sites (in this case, within the SIV/HIV-pol gene) through amino acids 1 to 6 of the 'recognition' alpha helix binding on to consecutive nucleotides in DNA in the *3' *to *5' *direction, a reverse pattern that can be confusing because the DNA target site is always numbered in the *5' *to *3' *direction, whereas amino acid sequences are numbered from *N *to *C *terminus (reviewed in [37]). Multi-ZF-arrays (like our three ZF-arrays) are generated by combining Finger 1 domains (F1) and Finger 3 domains (F3) that have been preselected to bind their cognate target sites in the context of the same Finger 2 domain (F2) [37]. Five of the 114 ZFAs generated are shown in table 1**(**for all, see additional file 1). A graphic map of the distribution of the recognition sites for the 114 multi-Zif-arrays obtained along the 3,182 bp length of the SIV/HIV-pol gene is shown in Figure 1. These ZFAs may be useful in future for purposes of directing novel or existing small artificial molecules to inhibit the SIV/HIV-pol gene specifically *in-vivo*, in a manner similar to those previously used by Segal *et al. *[31] and Eberhardy *et al*. [32] to repress HIV transcription. *Second, *using the alternate ZiFiT-CoDA-ZFN software set at default and adjusted to allow for a 5, 6, or 7 bp spacer region *plus *the FASTA sequences of the SIV/HIV-pol gene, we constructed two ZFNs with specificity to the SIV/HIV-pol-gene (see table 2 and additional file 2**) **[33-36]. These ZFNs cleave at positions approximately 1063/1089 and 1871/1895 within the SIV/HIV-pol gene. Each arm of these dimeric 3-ZF-*nucleases *recognizes nine base pairs (bp). This implies that the issuing ZFN dimer *in-vivo *will recognize an 18 + (5, 6, or 7 spacer) nucleotide-long region [37]. For instance, the two ZFNs in table 2 recognize, respectively, 25 and 23 bp within the HIV-pol gene. A graphic map of the distribution of the recognition sites for these two ZFNs built along the SIV/HIV-pol gene is shown in Figure 2. Using these two ZFNs, we argue that it may be possible to target and abrogate the SIV/HIV-pol gene by inducing double strand breaks (DSB) that can lead to excision of the region between positions 1063/1089 and 1871/1895 followed by non-homologous end-joining (NHEJ) [37]. Alternatively, however, using a set of 15 ZFNs that we generated by similar methods, which target and cleave within > 18 bp sequences of the entire HIV-1 genome [Genbank: NC_001802.1; >gi|9629357] and are here denoted Zif_HIV-1_F_N _(see Figure 3 and additional file 3), one may opt to excise over 80% of the latent provirus. Overall, using the PCR technique described by Kim *et al. *[22], and primers for gene sequences of both the DNA-cleavage domain of the Fok I endonuclease (F_N_: derived from Flavobacterium *Okeanokoites *and belonging to the type IIS class) and the Zif_HIV-pol _or Zif_HIV-1 _DNA-binding domain (see Table 2); fusion of the two sequences (Zif_HIV-pol _+F_N _or Zif_HIV-1_+F_N_) to yield a haploid copy of the hybrid, chimeric ZFN (Zif_HIV-pol_F_N _or Zif_HIV-1 _F_N_) gene with HIV-pol gene/HIV-1 provirus specificity can be achieved in a bacteria plasmid. *This intermediary step is necessary for cloning and biochemical characterization of the novel Zif*_*HIV-pol*_*F*_*N*_/Zif_HIV-1 _F_N _*gene and protein. *Specifically, characterization of the final cloned hybrid, chimeric ZFN (say-2xZif_HIV-pol_F_N_) gene and its expressed protein (REase) can respectively be done by (i) sequencing the target region of interest within the plasmid, and/or (ii) gel-electrophoretic extraction and biophysical profiling of the purified protein to determine its instability index, aliphatic index, theoretical pI, *in vivo *half life and grand average hydropathy (GRAVY) [13,22]. *These data are relevant for estimating the *in-vivo *ideal temperatures of function, solubility patterns in aqueous solution, and life-expectancies of the functional ZFN genotypes following expression *in-vivo. The specificity of these ZFAs and ZFNs can also further be enhanced through *in-vivo *modifications to the cleavage domain in order to generate a hybrid capable of functionally interrogating the ZFN dimer interface so as to prevent homodimerization, while still enhancing the efficiency of cleavage [38]. Further optimization within a bacteria-one hybrid (B1H) or yeast-one hybrid (Y1H) system may also be required [39].

**Table 1:** Five of the 114 ZFAs with binding specificity to sites within the SIV/HIV-pol gene

Position (n)	Zif #	target-DNA sequence	Zif array α-Helix (F1;F2;F3)
21-32	1	**31 tGCAGAGTGTc 21** **31 aCGTCTCACAg 21**	RHQHLKL; RQDNLGR; QSNVLSR

50-60	**2**	**50 aGAAGACAGGg 60** **50 tCTTCTGTCCc 60**	RRAHLLN; DRGNLTR; QSNNLNR

1515-1525	**56**	1515 **gGCAGAAGCA**g 1525 1515 cCGTCTTCGTc 1525	RGQELRR; QQTNLTR; QGNTLTR

1518-1528	**57**	1518 a**GAAGCAGAA**t 1528 1518 tCTTCGTCTTa 1528	QGSNLAR; QSTTLKR; QRNNLGR

3156-3166	**113**	3156 cGGAGAGGCTa 3166 3156 gCCTCTCCGAt 3166	NKQALDR; RQDNLGR; QANHLSR

NOTE: n is a position on a 1-to-3182 base-pair scale of the SIV/HIV-pol gene total nucleotide content, such that n+1714 (genomic context of first bp in gene) = actual genomic context of the target DNA specificity for the ZFA.

**Table 2:** The 2 ZFNs cleaving >18 bp sequences specifically within the HIV-pol gene

Zinc Finger Nuclease (ZFN)	Left Fn; triplet- α-Helix	Right Fn; triplet- α-Helix
-**target HIV-pol-gene 5**'		

**ZFN-unknown-SP-7-1**		
1063 gTTCTGCCTCAGGGATG**GAAGGGGTC**a 1089	F1; QGSNLAR;(**GAA**)	F1; TKSLLAR;(**GTC**)
1063 c**AAGACGGAG**TCCCTACCTTCCCCAGt 10891	F2; QSTTLKR;(**GCA**)	F2;RREHLVR;(**GGG**)
	F3; RGDNLNR;(**GAG**)	F3;QDGNLGR;(**GAA**)

**-target HIV-pol-gene 3'**		

**ZFN-unknown-SP-5-1**		
1871 cAACACCACCGCTAG**TAAGATTAG**t 1895	F1; AATALRR;(**GTT**)	F1; RSHNLRL;(**TAG**)
1871 g**TTGTGGTGG**CGATCATTCTAATCa 1895	F2; EAHHLSR;(**GGT**)	F2; VRHNLTR;(**GAT**)
	F3; IRHHLKR;(**GGT**)	F3; QQGNLQL;(**TAA**)

### Modeling the construct of lentiviral vectors for the specific delivery of a diploid copy of Zif-F_N _into CD4+ve cells

*Third, *lentiviral vectors (LVs)-by virtue of their unique ability to infect CD4 + cells inclusive of bone-marrow progenitor cell-lines, form an ideal vehicle for delivering and transducing the diploid copy of the SIV/HIV-pol gene/HIV-1 provirus-specific ZFNs (2**x**Zif_HIV-pol_F_N _and 2**x**Zif_HIV-1 _F_N_) identified and cloned above[7,40-42]. Over the past 10 years of our work with REases, LVs have emerged as potent and versatile vectors for *ex vivo *or *in vivo *gene transfer into dividing and non-dividing cells [15,41]. The latter -- ability to infect non-dividing cells - presents a unique opportunity when targeting of proviral HIV DNA in resting CD4 + memory cells is considered [5,6,42]. Moreover, in conjunction with zinc-finger nuclease technology and HIV, LVs allow for site-specific gene correction or addition in predefined chromosomal loci where proviral HIV resides [5,40,43]. Therefore, although other vectors such as adenoviruses and γ-retroviral vectors can be used to deliver either HIV-specific ZFN genotype, the unique advantages offered by LVs plus several design improvements underscore the safety and efficacy of LVs, with significant implications for proviral HIV reservoir targeting gene therapy in humans [43]. Specifically, robust phenotypic correction of diseases in mouse models has been achieved, paving the way toward the first clinical trials. LVs can deliver genes *ex vivo *into bona fide stem cells, particularly hematopoietic stem cells, allowing for stable transgene expression upon hematopoietic reconstitution. LVs can be pseudotyped with distinct viral envelopes that influence vector tropism and transduction efficiency [43]. Nonetheless, our ultimate goal -- expressing proviral HIV DNA-specific Zif-F_N _within dividing and non-dividing CD4+ mammalian cell lines *in-vivo *- calls for specialized LV constructs. *First*, because LVs are derived from HIV-1, a human pathogen, it is critically important to ensure that the corresponding LV is replication-defective. The latest generation LV technology has several built-in safety features that minimize the risk of generating replication-competent wild type human HIV-1 recombinants. Typically, LVs are generated by *trans*-complementation whereby packaging cells are co-transfected with a plasmid containing the vector genome and the packaging constructs that encode only the proteins essential for LV assembly and function. Lentiviral plasmid vectors are in principle constructed by deleting 5 of the 9 wild type HIV genes, specifically *vif*, *vpr*, *vpu*, *nef *and *tat*, leaving behind a gap-pol-rev expression plasmid skeleton [42-44]. The rev gene, which binds to the rev-response protein exportin (RRE) to enable nuclear transport of the lentivirus, is often replaced by either a simian rev/RRE system or the Mason-Pfizer constitutive transport element (CTE), which exploit other intra-cisternal type A elements (IAPE) such as the RNA transport element (RTE) other than the rev/RRE complex to export lentivirus RNA out of the nucleus [45]. *Secondly, *constructing the ultimate lentiviral plasmids encoding either the LV-2**x**Zif_HIV-pol_F_N _or the LV-2**x**Zif_HIV-1_F_N _genotype should exploit the design advanced by Oh *et al. *[44] comprising the HIV 5' long terminal repeat (LTR) fused with the Rous Sarcoma Virus (RSV) U5 region, and containing the phosphoglycerokinase (PGK) promoter required to drive the expression of a diploid copy of the hybrid bacterial, say the Zif_HIV-pol_F_N_, chimeric gene (or simply LV-2xZif_HIV-pol_F_N _particles). As a unique feature, a pair of splice donor (SD) and acceptor (SA) sites, the XbaI/NotI REase specificity sites separated by a 2A peptide, is required to enable PCR-based cloning of the diploid copy of the hybrid bacterial Zif_HIV-pol_F_N _or Zif_HIV-1_F_N _gene into the pHRSVcPGKnls backbone to yield the either LV-2**x**Zif_HIV-pol_F_N _or LV-2**x**Zif_HIV-1_F_N _transfer vector plasmid or particles as final products. Such multicistronic constructs, in which several proteins are encoded by a single messenger RNA, are commonly used in genetically engineered animals [45]. Although the use of an internal ribosomal entry site (IRES) was previously favored for multicistronic constructs, Tichas *et al*. [45] recently demonstrated the efficient use of the 2A peptide for bicistronic expression and co-translational cleavage in transgenic mice. The final LV-particles can then be produced recombinantly in large amounts by the known transient triple-plasmid transfection of 293T cells [40,42,44,46,47]. *In practice, *it is necessary that plasmids are at this stage evaluated for their gene-delivery and transduction potential using the protocols previously described by Oh *et al. *[44] and Mátrai *et al. *[42], but tailored to ZFN_HIV-pol _before their packaging. Ultimately, packaging cells are transfected with the lentiviral vector plasmid and three helper (packaging) constructs encoding Gag, Pol, Rev, and VSV-G. Only the vector contains the packaging sequence Ψ, whereas the packaging constructs are devoid of Ψ. The LV is flanked by the 5' and 3' LTR sequences that have promoter/enhancer activity and are essential for the correct expression of the full-length vector transcript. The LTRs also play important roles in reverse transcription and integration of the vector into the target cell genome. Overall, self-inactivating (SIN) LTR sequences that contain a partial deletion (Δ), Woodchuck post-transcriptional regulatory element (WPRE), central polypurine tract (cPPT), and Rev responsive element (RRE) are used. Assembled vector particles can then be harvested from the supernatant and, if required, subjected to further purification and concentration. Packaging LVs encoding different envelope genes only serves to allow for production of distinct LV pseudotypes with different tropisms [42].

#### 3. Testing the efficacy and safety of the lentiviral vectors delivering and transducing SIV/HIV-pol-gene specific, ZFN

*Thirdly and finally*, preclinical models for controlled testing of the safety and efficacy of LV- 2xZif_HIV-pol_F_N _or LV- 2xZif_HIV-1_F_N _may be devised using *either *active HIV-infected TZM-bl reporter cells (HeLa-derived JC53-BL cells that express high levels of CD4, CXCR4, and CCR5, and contain reporter cassettes for luciferase and β-galactosidase, both driven by the HIV-1 long terminal repeat); *or *latent-HIV-infected J-Lat cell lines that harbor a full-length HIV-1 genome that is transcriptionally competent and is integrated within actively transcribed cellular genes, but is inhibited at the transcriptional level [41,48]. Note, however, that the J-Lat cells may not offer us an appropriate model of latency, and Oh *et al*. [49] have recently established two novel cell lines latently infected with HIV-1 by limiting dilution cloning of resting A3.01 cells infected with HIV-1. These represent an alternative and better option to J-Lat cells for studying the molecular mechanisms of viral latency and development of anti-reservoir therapy of HIV-1 infection. *In the first instance, *I propose the innoculation of a single-parent culture of TZM-bl reporter cells on Dulbecco medium (DMEM), which is subsequently divided into two: a test-daughter (*td*) sample and a control-daughter (*cd*) sample. The *td*-sample is modified by transfection with, say, LV- 2**x**Zif_HIV-pol_F_N _to express Zif_HIV-pol_F_N _(the efficiency of Zif_HIV-pol_F_N _expression must be tested here, say by ELISA assays); the *cd *sample is left untreated. At time zero (T0), both *td *and *cd *samples are infected with HIV at infectious doses of 0.1, 0.2, 0.3 million particles per unit, after which they are cultured for a further 24-36 hours. The efficacy for abolition *or *disruption of HIV-pol gene expression can be measured by studying the level of abrogation in HIV's innate fitness to replicate and survive *in-vivo*, through measuring the level of chemiluminescence from the reporter cassettes for luciferase and β-galactosidase (expected to be diminished in *td *sample once Zif_HIV-pol_F_N _is highly efficacious, since reporter cassettes are driven by the HIV-1 long terminal repeat). This initial experiment essentially offers a model for testing the primary prevention of HIV infection by LV-2**x**Zif_HIV-pol_F_N _(a preventive vaccine mode). Safety should be evaluated by assaying and comparing levels of inflammatory cytokines, apoptotic DNA ladders, and targeted sequencing of proviral HIV integration hot spots (say via PCR amplification of the HIV-LTR) within the TZM-bl reporter cells in *td *relative to *cd-*samples (no significant differences are expected for a safe profile). *In the second alternative scenario*, using either J-Lat or the Oh *et al*. [49] cell lines that offer us an *in vitro *model of HIV-1 latency, we can devise a model for testing the potency of LV-2**x**Zif_HIV-pol_F_N _towards the end-goal of HIV therapeutic cure and latent provirus eradication [47]. Specifically, a parent culture of J-Lat or Oh *et al*. [49] cells maintained on DMEM is divided into a *td*- and *cd*- sample. As above, the *td-*sample is transfected with (say) LV-2**x**Zif_HIV-pol_F_N _at time zero (T0) and the extent of Zif_HIV-pol_F_N _expression again measured, say by ELISA-assays, while the *cd*-sample is left untreated. The efficacy of LV-2**x**Zif_HIV-pol_F_N _in irreversibly abrogating the innate fitness of the HIV provirus to replicate within latently infected cells through the abolition *or *disruption of HIV-pol gene/genome action can be measured by studying the level of fluorescence (a marker of latent provirus, and one expected to be low in the *td*-sample once Zif_HIV-pol_F_N _are expressed and efficacious); following the addition of agents that exorcise proviral HIV-DNA [5,8-11]. This assay should be facilitated by ensuring that the latent provirus integrated in the Oh *et al*. [48] cell lines, as in J-Lat cells, also includes the GFP gene [48]. The latter would provide us with a fluorescent marker of HIV-1 transcriptional activity. Again, safety here can be evaluated by assaying and comparing levels of inflammatory cytokines, apoptotic DNA ladders, and targeted sequencing of proviral HIV integration hot spots (say via PCR amplification of HIV-LTR) within the J-Lat or Oh *et al*. [49] cells in *td *relative to *cd-*samples (no significant differences are expected in respect of safety).

#### 4. Availability: Databases and software

- The ZFN consortium CoDA-ZiFiT-ZFA/ZFN software and algorithms used are available at the following url: http://www.zincfingers.org/scientific-background.htm

- The NCBI gene database hosting the HIV-pol gene and HIV-1 whole genome are available at the following url:

(i)http://www.ncbi.nlm.nih.gov/nuccore/NC_001870.1

(ii) http://www.ncbi.nlm.nih.gov/nuccore/9629357?report=fasta

### General discussion

I report here SIV/HIV-pol gene and HIV-1 whole genome specific zinc finger nucleases, which are proposed for use towards targeted HIV gene therapy. Specifically, because of the notoriety and promiscuousness of HIV at evading previous therapeutic and vaccine attempts, we - building on the bacterial R-M enzymatic machinery as a primitive anti-viral model and prior work identifying bacterial REases against SIV/HIV genomes - postulated that *ex-vivo *or even *in-vivo *disruption of viral gene action or excision of over 80% of proviral HIV DNA from within infected cells can irreversibly inactivate both active and latent virus [4-7,12-14]. The SIV/HIV-specific bacterial REases previously identified towards this purpose also target short palindromic targets present within the human genome and thereby carry the risk for toxicity [15]. Now, however, in the wake of advances in zinc finger technology, I have assembled 114 ZFAs (Figure 1, Table 1, and Additional file 2) and constructed 2 ZFNs (Figure 2, Table 2, and Additional file 2) with unique specificity to >18 bp sequences present only within the SIV/HIV pol gene. In addition, another 15 ZFNs were constructed that target and cleave within the >18 bp sequences present only within the proviral DNA of the whole HIV-1 genome (see Figure 3 for graphic distribution of the cleavage sites and pattern. For details of the latter, see additional file 3). It is therefore speculated that lentiviral vectors carrying either genotype (**LV-2xZif**_**HIV-pol**_**F**_**N **_or **LV- 2xZif**_**HIV-1**_**F**_**N**_) may offer the *ex-vivo *or even *in-vivo *experimental opportunity to halt HIV replication functionally by directly-either abrogating HIV-pol gene action or excising over 80% of proviral HIV dsDNA from latently infected cells [40,42].

*Several potential limitations are contingent on the above proposition that readers should take into account, as these may require addressing before this technology is moved from the lab into human trials. First*, the possibility of genome toxicity, though minimized by the shift from our prior REase model to hybrid ZFN prototypes, remains and underlines the rationale for conducting the above suggested genome-safety profiling [14,18,36]. In this regard, perhaps the HIV-pol gene or HIV-1 whole genome specificity of those 3-zinc finger *nucleases *identified in this study may benefit from further modular enhancements towards 4, 5, or 6 finger arrays [37]. The specificity of such multi-finger proteins can also be enhanced by *in-vitro *optimization using a bacteria-one hybrid (B1H) or yeast-one-hybrid (Y1H) system [39]. Moreover, modifications to the cleavage domain in order to generate a hybrid capable of functionally interrogating the ZFN dimer interface so as to prevent homodimerization, while still enhancing the efficiency of cleavage, are equally possible [38]. ***Second***, clinical trails of lentiviral vectors are still limited globally, a fact that may hinder the global use of the technology, particularly within the low and middle income countries most affected by the HIV epidemic [2]. Outweighing these potential shortcomings, though, is that LV technology has greatly improved over the past decade [40,42]. Moreover, LVs offer us the added user-friendly advantage that they may be directly administered to patients via intravenous (IV) or intra-osseous (IO) *in-vivo *routes and yet still effect a therapeutically adequate gene delivery and transduction for HIV preventive or therapeutic purposes (vaccines); though this may be less than the up-to-17% achieved by *ex-vivo *routes [28,42]. Overall, for purposes of targeting latent proviral HIV reservoir, the likelihood that *in-vivo *delivered LVs would ever find and effectively transduce a latently-infected cell with the diploid copy of the ZFN remains to be established, considering that those latently infected cells might be circulating randomly all around the body in the blood [5,6]. Perhaps experiments to evaluate the efficiency and extent of *in-vivo *LV-delivery using humanized mice, as Wilen *et al. *[43] recently did, and fluorescent labeled LVs, may suffice here. Until such experiments establish these *in-vivo *LV-delivery routes as adequate, however, the already proven *ex-vivo *alternative remains most viable [27,28,43]. *Alternatively, *since only about 1 in 1,000,000 memory T-cells are latently infected in the body, they are hard targets to hit by LVs delivered directly *in-vivo*, and more strategies may be required, either to enhance the above-presented model or act as completely novel *in-vivo *ZFN-delivery vehicles [42,43]. Also, the efficiency of targeted mutagenesis by LVs delivered in this manner, which would have to be extremely high in order to affect enough cells to be useful, remains questionable yet relevant to know, since even a small residual reservoir of cells carrying the provirus would be sufficient to restart a systemic infection [5,23-28]. One may, however, counter this reasoning by arguing that *neither *all *nor *any resting memory CD+ cells need to be modified by LVs in order to halt the buildup of a re-infection functionally. Specifically, once the newly emerging active (non-resting) CD4+ve cells from the progenitor cell-lines are all resistant, any new HIV particles will have no active CD4+ cells to infect and propagate in. In addition, there could be several proviral HIV integration sites in a single CD4+ cell genome, unlike the loci for host gene targets such as CCR5, and that presents another challenge and yet an opportunity for LVs to wipe out HIV efficaciously from within infected cells[5,6,27,28,42]. Despite all the above reservations surrounding the efficiency of gene delivery associated with *in-vivo *relative to *ex-vivo routes*, we can maintain that further exploration of novel LV designs for *in-vivo *delivery may circumvent these obstacles and allow for a wider usability of these HIV gene- or genome-specific ZFNs as therapeutics, particularly since this would eliminate the need for the costly process of autologous bone marrow pre-harvest, ZFN-engineering and subsequent transplantation [12,13,28]. That would make these treatments easily accessible to people within resource-limited settings where HIV is most prevalent and the former science is lacking. Induction of a type I interferon response that reduces LVs availability is possible, but this is likely to be very minimal among persons living with HIV or AIDS who are in late stages of immunosuppression, while use of steroid or non-myeloablative preparative regimens may be required for those not yet immunocompromised [50,51]. *Third*, HIV is notorious for evolving mutations that lead to resistance to drugs, and ZFNs are no exceptions. For instance, it is possible that HIV, by evolving single base substitutions, may evade the specificity of REases [4,5]. Nonetheless, we note that because many mutations leading to resistance to HAART are single point mutations, and ZFN-resistance mutations may need to accrue in >18 bp, such cumulative substitutions are likely to be selectively disfavored since the progeny virus possessing them is phenotypically disadvantaged [4,52]. *Last but not least*, many lentiviral vector plasmids exploit a gap-pol core as the expression plasmid-skeleton, which raises the possibility that the LV-plasmid's pol-gene component may itself be targeted by our HIV specific Zinc Finger Nucleases, particularly Zif_HIV-pol_F_N_. To eliminate this possibility, perhaps the design for the Zif_HIV-pol_F_N _genotype carrying LV-plasmids should exploit constructs recently described by Oh *et al. *[44], which comprise just the HIV 5' long terminal repeat fused with the Rous Sarcoma Virus U5 region and use the phosphoglycerokinase (PGK) promoter to drive the expression of the diplod copy of Zif_HIV-pol_F_N_. Only the cis-acting DNA element known as the central polypurine tract sequence (or cppt) from the pol gene is included 5' to PGK [42,46].

In conclusion, on the basis of the hypothesis that *in-vivo *abolition of HIV gene action *or *disruption/excision of over 80% of proviral HIV DNA can abrogate HIV's innate fitness to replicate and survive, I engineer HIV-pol gene and HIV-1 whole genome specific Zinc Finger Nucleases (ZFNs) and advance the protocol for constructing and pre-clinically testing lentiviral vectors that deliver and transduce a diploid copy of either ZFN genotype **(LV-2xZif**_**HIV-pol**_**F**_**N **_or **LV- 2xZif**_**HIV-pol**_**F**_**N**_**)**. **LV-2xZif**_**HIV-pol**_**F**_**N **_and **LV- 2xZif**_**HIV-1**_**F**_**N **_may offer the *ex-vivo *or even *in-vivo *experimental opportunity to halt HIV replication functionally by directly abrogating HIV-pol gene action or disrupting/excising over 80% of proviral HIV dsDNA from latently infected cells.

## Competing interests

WM is Chief Scientific Officer at Restrizymes Biotherapeutics (U) Ltd, Kampala-Uganda, and a member of the steering committee of the Young and Early Careers' Investigators (YECI) of the Global HIV Vaccine Enterprise.

## Authors' contributions

WM conceived the idea for this article, designed and undertook the experiments, and wrote the MS. All authors read and approved the final manuscript.

## Supplementary Material

Additional file 1 **A detailed list of the Multi-Zif assembly targeting sequences of the SIV/HIV-pol-gene**. This file offers a list of the 114 ZFAs that target specific DNA sequences within the SIV/HIV-pol-gene; detailing their alpha-helical recognition sequences and target DNA sites. Click here for file

Additional file 2 **Two ZFNs cleaving within the SIV/HIV-pol gene**. This file offers a list of the two zinc finger nucleases that specifically target and cleave within DNA sequences of the SIV/HIV-pol gene, detailing their alpha-helical recognition sequences and target DNA sites. Click here for file

Additional file 3 **A detailed list of the 15 ZFN cleaving within the HIV-1 whole genome**. This file offers a list of the 15 zinc finger *nucleases *that specifically target and cleave within >18 DNA- bp-sequences of the HIV-1 whole genome; detailing their alpha helical recognition sequences, and target-DNA sites. Click here for file

## References

[B1] Gallo RC Montagnier L The Discovery of HIV as the Cause of AIDS N Engl J Med 2003 24 2283 2285 349 10.1056/NEJMp038194 14668451

[B2] CDC Pneumocystis carinii pneumonia among persons with hemophilia A MMWR 1982 31 365 7 6815443

[B3] UNAIDS & World Health Organization. AIDS Epidemic Update 2009 Geneva

[B4] Wayengera M Kajumbula H Byarugaba W Harnessing pharmacogenomics to tackle resistance to the "Nucleoside Reverse Transcriptase Inhibitor" backbone of highly active antiretroviral therapy in resource limited settings Open AIDS Journal 2008 2 78 81 10.2174/1874613600802010078 19274067 PMC2627514

[B5] Wayengera M Targeting persistent HIV: Where and how, if possible? HIV & AIDS Review 2011 10 821780031

[B6] Onafuwa-Nuga A McNamara LA Collins KL Towards a cure for HIV: the identification and characterization of HIV reservoirs in optimally treated people Cell Res 2011 20 11 1185 1187 10.1038/cr.2010.140 20877313

[B7] The Council of the Global HIV Vaccine Enterprise The 2010 scientific strategic plan of the Global HIV Vaccine Enterprise Nature Med 2010 16 981 989 10.1038/nm0910-981 20823883

[B8] Antoni BA Rabson AB Kinter A Bodkin M Poli G NF-kappa B dependent and -independent pathways of HIV activation in a chronically infected T cell line Virology 1994 202 684 694 10.1006/viro.1994.1390 7913275

[B9] Contreras X Barboric M Lenasi T Peterlin BM HMBA releases P-TEFb from HEXIM1 and 7SK snRNA via PI3K/Akt and activates HIV transcription PLoS Pathog 2007 3 10 e146 10.1371/journal.ppat.0030146 PMC2014796 17937499

[B10] Mehla R Bivalkar-Mehla S Zhang R Handy I Albrecht H Giri S Nagarkatti P Nagarkatti M Chauhanet A Bryostatin Modulates Latent HIV-1 Infection via PKC and AMPK Signaling but Inhibits Acute Infection in a Receptor Independent Manner PLoS ONE 2010 5 6 e11160 10.1371/journal.pone.0011160 20585398 PMC2886842

[B11] Choudhary SK Margolis DM Curing HIV: Pharmacologic Approaches to Target HIV-1 Latency Annu Rev Pharmacol Toxicol 2011 51 397 418 10.1146/annurev-pharmtox-010510-100237 21210747 PMC3958947

[B12] Wayengera M HIV and Gene Therapy: The proposed [R-M enzymatic] model for a gene therapy against HIV Makerere Med J 2003 38 28 30

[B13] Wayengera M Kajumbula H Byarugaba W Identification of restriction endonuclease with potential ability to cleave the HSV-2 genome: inherent potential for biosynthetic versus live microbicides BMC Theor Biol Med Model 2008 5 18 10.1186/1742-4682-5-18 PMC2526989 18687114

[B14] Wayengera M Why bacteria derived R-M nucleic enzymatic peptides are efficient therapeutic molecules for use in the design and development of novel HIV therapeutic molecules Afr J Biotechnol 2008 7 12 1791 1796

[B15] Wayengera M Kajumbula H Byarugaba W Frequency and site mapping of HIV-1/SIVcpz, HIV-2/SIVsmm and Other SIV gene sequence cleavage by various bacteria restriction enzymes: Precursors for a novel HIV inhibitory product Afr J Biotechnol 2007 6 10 1225 1232

[B16] Wayengera M A Recombinant lactobacillus strain producing restriction enzymes that cleave proviral HIV DNA may offer a novel Vagino-cervical mucosal exogenous live Microbicide strategy for preventing HIV transmission among high-risk women Afr J Biotechnol 2007 6 15 1750 1756

[B17] Wayengera M PREX-1979: Modeling the first ever prototype of could be a 5^th ^generation of Microbicides for preventing HIV infection among high-risk women Afr J Biotechnol 2007 6 10 1221 1224

[B18] Wayengera M Pre-Integration gene slicing (PRINT-GSX) as an alternate or complimentary gene therapy modem to RNA interference J Appl Biol Sci 2008 1 2 56 63

[B19] Wayengera M Diverting primary HIV entry and replication to vaginal commensal lactobacillus expressing R-M nucleic enzymatic peptides with potent activity at cleaving proviral DNA as a novel HIV live microbicide strategy Microbicide- New Delhi, India 2008 Abs-10

[B20] Wayengera M Preparing for a Phase 1 Preclinical trial of VRX-SMR: a Lentiviral Vector transduced with restriction enzymes cleaving HIV proviral DNA as a therapeutic vaccine: Opportunities and Challenges Vaccine Congress -Amsterdam, Netherlands 2007 24OR

[B21] Wayengera M *xREPLAB*: A recombinant lactobacillus strain producing restriction enzymes with potent activity against HIV proviral DNA as a Live Microbicide Strategy AIDS vaccine- Washington, Seattle 2007 P05 01

[B22] Kim YG Cha J Chandrasegaran S Hybrid restriction enzymes: zinc finger fusions to Fok I cleavage domain Proc Natl Acad Sci USA 1996 93 3 1156 1160 10.1073/pnas.93.3.1156 8577732 PMC40048

[B23] Cathomen T Joung JK Zinc-finger nucleases: the next generation emerges Mol Ther 2008 16 7 1200 1207 10.1038/mt.2008.114 18545224

[B24] Guo J Gaj T Barbas CF Directed Evolution of an Enhanced and Highly Efficient FokI Cleavage Domain for Zinc Finger Nucleases Journal of Molecular Biology 2010 400 1 96 10.1016/j.jmb.2010.04.060 20447404 PMC2885538

[B25] Durai S Mani M Kandavelou K Wu J Porteus MH Chandrasegaran S Zinc finger nucleases: custom-designed molecular scissors for genome engineering of plant and mammalian cells Nucleic Acids Res 2005 33 18 5978 5990 10.1093/nar/gki912 16251401 PMC1270952

[B26] Porteus MH Carroll D Gene targeting using zinc finger nucleases Nat Biotechnol 2005 23 8 967 73 10.1038/nbt1125 16082368

[B27] Perez E Wang J Miller JC Jouvenot Y Kim KA Liu O Wang N Lee G Bartsevich VV Lee YL Guschin DY Rupniewski I Waite AJ Carpenito C Carroll RG Orange JS Urnov FD Rebar EJ Ando D Gregory PD Riley JL Holmes MC June CH Establishment of HIV-1 resistance in CD4+ T cells by genome editing using zinc-finger nucleases Nat Biotechnol 2008 26 7 808 816 10.1038/nbt1410 18587387 PMC3422503

[B28] Holt N Wang J Kim K Friedman G Wang X Taupin V Crooks GM Kohn DB Gregory PD Holmes MC Cannon PM Human hematopoietic stem/progenitor cells modified by zinc-finger nucleases targeted to CCR5 control HIV-1 *in vivo* Nat Biotechnol 2010 28 8 839 847 10.1038/nbt.1663 20601939 PMC3080757

[B29] Grosse S Huot N Mahiet C Arnould S Barradeau S Clerre DL Chion-Sotinel I Jacqmarcq C Chapellier B Ergani A Desseaux C Cédrone F Conseiller E Pâques F Labetoulle M Smith J Meganuclease-mediated Inhibition of HSV1 Infection in Cultured Cells Mol Ther 2011 19 4 694 702 10.1038/mt.2010.302 21224832 PMC3070101

[B30] Cradick TJ Keck K Bradshaw S Jamieson AC McCaffrey AP Zinc-finger Nucleases as a Novel Therapeutic Strategy for Targeting Hepatitis B Virus DNAs Mol Ther 2010 18 5 947 954 10.1038/mt.2010.20 20160705 PMC2890117

[B31] Segal DJ Goncalves J Eberhardy SR Swan CH Torbett BE Li X Barbas CF Attenuation of HIV-1 Replication in Primary Human Cells with a Designed Zinc Finger Transcription Factor J Biochem 2004 279 15 14509 14519 10.1074/jbc.M400349200 14734553

[B32] Eberhardy SR Goncalves J Coelho S Segal DJ Berkhout B Barbas CF Inhibition of Human Immunodeficiency Virus Type 1 Replication with Artificial Transcription Factors Targeting the Highly Conserved Primer-Binding Site J Virol 2006 80 6 2873 2883 10.1128/JVI.80.6.2873-2883.2006 16501096 PMC1395442

[B33] Dreier B Segal DJ Barbas CF Insights into the molecular recognition of the 5 '-GNN-3 ' family of DNA sequences by zinc finger domains J Mol Biol 2000 303 4 489 502 10.1006/jmbi.2000.4133 11054286

[B34] Mandell JG Barbas CF Zinc finger tools: custom DNA-binding domains for transcription factors and nucleases Nucleic Acids Res 2006 34 Sp.Iss. SI W516 W523 16845061 10.1093/nar/gkl209 PMC1538883

[B35] Sander JD Zaback PZ Joung JK Voytas DF Dobbs D Zinc Finger Targeter (ZiFiT): an engineered zinc finger/target site design tool Nucleic Acids Research 2007 35 W599 605 10.1093/nar/gkm349 17526515 PMC1933188

[B36] Sander JD Dahlborg EJ Goodwin MJ Cade L Zhang F Cifuentes D Curtin SJ Blackburn JS Thibodeau-Beganny S Qi Y Pierick CJ Hoffman E Maeder ML Khayter C Reyon D Dobbs D Langenau DM Stupar RM Giraldez AJ Voytas DF Peterson RT Yeh JR Joung JK Selection-free zinc-finger-nuclease engineering by context-dependent assembly (CoDA) Nat Methods 2011 8 1 67 69 10.1038/nmeth.1542 21151135 PMC3018472

[B37] Urnov FD Rebar EJ Holmes MC Zhang HS Gregory PD Genome editing with engineered zinc finger nucleases Nat Rev Genet 2010 11 9 636 646 S, 10.1038/nrg2842 20717154

[B38] Doyon Y Vo TD Mendel MC Greenberg SG Wang J Xia DF Miller JC Urnov FD Gregory PD Holmes MC Enhancing zinc-finger-*nuclease *activity with improved obligate heterodimeric architectures Nat Methods 2011 8 1 74 79 10.1038/nmeth.1539 21131970

[B39] Herrmann F Garriga-Canut M Baumstark R Fajardo-Sanchez E Cotterell J Minoche A Himmelbauer H Isalan M p53 Gene Repair with Zinc Finger Nucleases Optimised by Yeast 1-Hybrid and Validated by Solexa Sequencing PLoS ONE 2011 6 6 e20913 10.1371/journal.pone.0020913 21695267 PMC3111460

[B40] Naldini L Blomer U Gallay P Ory D Mulligan R Gage FH Verma IM Trono D In vivo gene delivery and stable transduction of non dividing cells by a lentiviral vector Science 1996 272 263 267 10.1126/science.272.5259.263 8602510

[B41] Jordan A Bisgrove D Verdin E HIV reproducibly establishes a latent infection after acute infection of T cells in vitro Embo J 2003 22 1868 1877 10.1093/emboj/cdg188 12682019 PMC154479

[B42] Mátrai J Chuah MK VandenDriessche T Recent advances in lentiviral vector development and applications Mol Ther 2010 18 3 477 490 10.1038/mt.2009.319 20087315 PMC2839421

[B43] Wilen CB Wang J Tilton JC Miller JC Kim KA Rebar EJ Sherrill-Mix SA Patro SC Secreto AJ Jordan APO1 Lee G Kahn J Aye PP Bunnell BA Lackner AA Hoxie JA Danet-Desnoyers GA Bushman FD Riley JL Gregory PD June CH Holmes MC Doms RM Engineering HIV-Resistant Human CD4+ T Cells with CXCR4-Specific Zinc-Finger Nucleases PLoS Pathog 2011 7 4 e1002020 10.1371/journal.ppat.1002020 21533216 PMC3077364

[B44] Oh T Bajwa A Jia G Park F Lentiviral vector design using alternative RNA export elements Retrovirology 2007 4 38 10.1186/1742-4690-4-38 17550606 PMC1904242

[B45] Trichas G Begbie J Srinivas S Use of the viral 2A peptide for bicistronic expression in transgenic mice BMC Biol 2008 6 40 10.1186/1741-7007-6-40 18793381 PMC2553761

[B46] Zufferey R Dull T Mandel RJ Bukovsky A Quiroz D Naldini L Trono D Self-inactivating lentivirus vector for safe and efficient in vivo gene delivery J Virol 1998 72 9873 9880 9811723 10.1128/jvi.72.12.9873-9880.1998 PMC110499

[B47] Wodrich H Schambach A Krausslich HG Multiple copies of the Mason-Pfizer monkey virus constitutive RNA transport element lead to enhanced HIV-1 Gag expression in a contextdependent manner Nucleic Acids Res 2000 28 901 910 10.1093/nar/28.4.901 10648781 PMC102582

[B48] Kauder SE Bosque A Lindqvist A Planelles V Verdin E Epigenetic regulation of HIV-1 latency by cytosine methylation PLoS Pathog 2009 5 6 e1000495 10.1371/journal.ppat.1000495 19557157 PMC2695767

[B49] Oh YT Kim KC Hong KJ Lee HS Jang DH Lee JS Choi SY Kim SS Choi BS Establishment of novel cell lines latently infected with human immunodeficiency virus 1 Acta Virol 2011 55 2 155 9 10.4149/av_2011_02_155 21692564

[B50] Brown BD Sitia G Annoni A Hauben E Sergi LS Zingale A Roncarolo MG Guidotti LG Naldini L In vivo administration of lentiviral vectors triggers a type I interferon response that restricts hepatocyte gene transfer and promotes vector clearance Blood 2007 109 7 2797 805 17170119 10.1182/blood-2006-10-049312

[B51] Kawai T Cosimi AB Spitzer TR Tolkoff-Rubin N Suthanthiran M Saidman SL Shaffer J Preffer FI Ding R Sharma V Fishman JA Dey B Ko DS Hertl M Goes NB Wong W Williams WW Jr Colvin RB Sykes M Sachs DH HLA-mismatched renal transplantation without maintenance immunosuppression N Engl J Med 2008 358 4 353 361 10.1056/NEJMoa071074 18216355 PMC2819046

[B52] Wayengera M Identity of zinc finger nucleases with specificity to Herpes simplex virus type II genomic DNA: novel HSV-2 vaccine/therapy precursors Theor Biol Med Model 2011 8 1 23 10.1186/1742-4682-8-23 21702927 PMC3138452

