# Supplementary material for: Proviral HIV-genome-wide and pol-gene specific Zinc Finger Nucleases: Usability for targeted HIV gene therapy
Source: Theor Biol Med Model. 2011 Jul 22;8:26. doi: 10.1186/1742-4682-8-26 (PMC3152896; doi:10.1186/1742-4682-8-26)
Supplement: Additional file 1 — A detailed list of the Multi-Zif assembly targeting sequences of the SIV/HIV-pol-gene. This file offers a list of the 114 ZFAs that target specific DNA sequences within the SIV/HIV-pol-gene; detailing their alpha-helical recognition sequences and target DNA sites. [file 1742-4682-8-26-S1.DOC]

**Zinc Finger Site Type:** Array
**Zinc Finger Engineering Method:** CoDA
**Sequence Name** : SIV/HIV pol
**Sequence Length**:3182
**Nucleotide Sequence** :nTTGGAATTGTGGGAAAGAGGGACACTCTGCAAGGCAATGCAGAGCCCCAAGAAGACAGGGATGCTGGAAATGTGGAAAAATGGACCATGTTATGGCCAAATGCCCAGACAGACAGGCGGGTTTTTTAGGCCTTGGTCCATGGGGAAAGAAGCCCCGCAATTTCCCCATGGCTCAAGTGCATCAGGGGCTGATGCCAACTGCTCCCCCAGAGGACCCAGCTGTGGATCTGCTAAAGAACTACATGCAGTTGGGCAAGCAGCAGAGAGAAAAGCAGAGAGAAAGCAGAGAGAAGCCTTACAAGGAGGTGACAGAGGATTTGCTGCACCTCAATTCTCTCTTTGGAGGAGACCAGTAGTCACTGCTCATATTGAAGGACAGCCTGTAGAAGTATTACTGGATACAGGGGCTGATGATTCTATTGTAACAGGAATAGAGTTAGGTCCACATTATACCCCAAAAATAGTAGGAGGAATAGGAGGTTTTATTAATACTAAAGAATACAAAAATGTAGAAATAGAAGTTTTAGGCAAAAGGATTAAAGGGACAATCATGACAGGGGACACCCCGATTAACATTTTTGGTAGAAATTTGCTAACAGCTCTGGGGATGTCTCTAAATTTTCCCATAGCTAAAGTAGAGCCTGTAAAAGTCGCCTTAAAGCCAGGAAAGGATGGACCAAAATTGAAGCAGTGGCCATTATCAAAAGAAAAGATAGTTGCATTAAGAGAAATCTGTGAAAAGATGGAAAAGGATGGTCAGTTGGAGGAAGCTCCCCCGACCAATCCATACAACACCCCCACATTTGCTATAAAGAAAAAGGATAAGAACAAATGGAGAATGCTGATAGATTTTAGGGAACTAAATAGGGTCACTCAGGACTTTACGGAAGTCCAATTAGGAATACCACACCCTGCAGGACTAGCAAAAAGGAAAAGAATTACAGTACTGGATATAGGTGATGCATATTTCTCCATACCTCTAGATGAAGAATTTAGGCAGTACACTGCCTTTACTTTACCATCAGTAAATAATGCAGAGCCAGGAAAACGATACATTTATAAGGTTCTGCCTCAGGGATGGAAGGGGTCACCAGCCATCTTCCAATACACTATGAGACATGTGCTAGAACCCTTCAGGAAGGCAAATCCAGATGTGACCTTAGTCCAGTATATGGATGACATCTTAATAGCTAGTGACAGGACAGACCTGGAACATGACAGGGTAGTTTTACAGTCAAAGGAACTCTTGAATAGCATAGGGTTTTCTACCCCAGAAGAGAAATTCCAAAAAGATCCCCCATTTCAATGGATGGGGTACGAATTGTGGCCAACAAAATGGAAGTTGCAAAAGATAGAGTTGCCACAAAGAGAGACCTGGACAGTGAATGATATACAGAAGTTAGTAGGAGTATTAAATTGGGCAGCTCAAATTTATCCAGGTATAAAAACCAAACATCTCTGTAGGTTAATTAGAGGAAAAATGACTCTAACAGAGGAAGTTCAGTGGACTGAGATGGCAGAAGCAGAATATGAGGAAAATAAAATAATTCTCAGTCAGGAACAAGAAGGATGTTATTACCAAGAAGGCAAGCCATTAGAAGCCACGGTAATAAAGAGTCAGGACAATCAGTGGTCTTATAAAATTCACCAAGAAGACAAAATACTGAAAGTAGGAAAATTTGCAAAGATAAAGAATACACATACCAATGGAGTGAGACTATTAGCACATGTAATACAGAAAATAGGAAAGGAAGCAATAGTGATCTGGGGACAGGTCCCAAAATTCCACTTACCAGTTGAGAAGGATGTATGGGAACAGTGGTGGACAGACTATTGGCAGGTAACCTGGATACCGGAATGGGATTTTATCTCAACACCACCGCTAGTAAGATTAGTCTTCAATCTAGTGAAGGACCCTATAGAGGGAGAAGAAACCTATTATACAGATGGATCATGTAATAAACAGTCAAAAGAAGGGAAAGCAGGATATATCACAGATAGGGGCAAAGACAAAGTAAAAGTGTTAGAACAGACTACTAATCAACAAGCAGAATTGGAAGCATTTCTCATGGCATTGACAGACTCAGGGCCAAAGGCAAATATTATAGTAGATTCACAATATGTTATGGGAATAATAACAGGATGCCCTACAGAATCAGAGAGCAGGCTAGTTAATCAAATAATAGAAGAAATGATTAAAAAGTCAGAAATTTATGTAGCATGGGTACCAGCACACAAAGGTATAGGAGGAAACCAAGAAATAGACCACCTAGTTAGTCAAGGGATTAGACAAGTTCTCTTCTTGGAAAAGATAGAGCCAGCACAAGAAGAACATGATAAATACCATAGTAATGTAAAAGAATTGGTATTCAAATTTGGATTACCCAGAATAGTGGCCAGACAGATAGTAGACACCTGTGATAAATGTCATCAGAAAGGAGAGGCTATACATGGGCAGGCAAATTCAGATCTAGGGACTTGGCAAATGGATTGTACCCATCTAGAGGGAAAAATAATCATAGTTGCAGTACATGTAGCTAGTGGATTCATAGAAGCAGAGGTAATTCCACAAGAGACAGGAAGACAGACAGCACTATTTCTGTTAAAATTGGCAGGCAGATGGCCTATTACACATCTACACACAGATAATGGTGCTAACTTTGCTTCGCAAGAAGTAAAGATGGTTGCATGGTGGGCAGGGATAGAGCACACCTTTGGGGTACCATACAATCCACAGAGTCAGGGAGTAGTGGAAGCAATGAATCACCACCTGAAAAATCAAATAGATAGAATCAGGGAACAAGCAAATTCAGTAGAAACCATAGTATTAATGGCAGTTCATTGCATGAATTTTAAAAGAAGGGGAGGAATAGGGGATATGACTCCAGCAGAAAGATTAATTAACATGATCACTACAGAACAAGAGATACAATTTCAACAATCAAAAAACTCAAAATTTAAAAATTTTCGGGTCTATTACAGAGAAGGCAGAGATCAACTGTGGAAGGGACCCGGTGAGCTATTGTGGAAAGGGGAAGGAGCAGTCATCTTAAAGGTAGGGACAGACATTAAGGTAGTACCCAGAAGAAAGGCTAAAATTATCAAAGATTATGGAGGAGGAAAAGAGGTGGATAGCAGTTCCCACATGGAGGATACCGGAGAGGCTAGAGAGGTGGCATAGn
**Selected Module Sets:**
**Selected Module Count:** 3
**Ignore Asp Overlap:** True

The results below are zinc finger arrays that can be constructed using CoDA. Note that other methods (including modular assembly and OPEN) can also potentially be used to target the input sequence of interest.”

**Sort By:**

**[

](http://zifit.partners.org/ZiFiT/CoDAZiFiTArray.aspx#ctl00_ContentPlaceHolder1_tree12_SkipLink)**

| ZFA-unknown-1 31 t[GCAGAGTGT](http://bindr.gdcb.iastate.edu:8080/ZiFDB/controller/searchArray?site=TGTGAGGCA)c 21  31 aCGTCTCACAg 21 |
| --- |

|  | 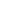 | | | FINGER | HELIX | TRIPLET | REFERENCE NUMBER | SOURCE | | --- | --- | --- | --- | --- | | F1 | RHQHLKL | [TGT](http://bindr.gdcb.iastate.edu:8080/ZiFDB/controller/searchFinger?target=TGT) | - | CoDA | | F2 | RQDNLGR | [GAG](http://bindr.gdcb.iastate.edu:8080/ZiFDB/controller/searchFinger?target=GAG) | - | CoDA | | F3 | QSNVLSR | [GCA](http://bindr.gdcb.iastate.edu:8080/ZiFDB/controller/searchFinger?target=GCA) | - | CoDA |   [ZF DNA Sequence](javascript:CoDAPopupArrayWindow("ZFA-unknown-1","RHQHLKL","RQDNLGR","QSNVLSR")) | |
| --- | --- | --- | --- | --- | --- | --- | --- | --- | --- | --- | --- | --- | --- | --- | --- | --- | --- | --- | --- | --- | --- | --- | --- | --- |
| [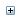](javascript:TreeView_ToggleNode(ctl00_ContentPlaceHolder1_tree12_Data,2,document.getElementById('ctl00_ContentPlaceHolder1_tree12n2'),'%20',document.getElementById('ctl00_ContentPlaceHolder1_tree12n2Nodes'))) | | ZFA-unknown-2 50 a[GAAGACAGG](http://bindr.gdcb.iastate.edu:8080/ZiFDB/controller/searchArray?site=AGGGACGAA)g 60  50 tCTTCTGTCCc 60 | |  |

|  | 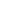 | | | FINGER | HELIX | TRIPLET | REFERENCE NUMBER | SOURCE | | --- | --- | --- | --- | --- | | F1 | RRAHLLN | [AGG](http://bindr.gdcb.iastate.edu:8080/ZiFDB/controller/searchFinger?target=AGG) | - | CoDA | | F2 | DRGNLTR | [GAC](http://bindr.gdcb.iastate.edu:8080/ZiFDB/controller/searchFinger?target=GAC) | - | CoDA | | F3 | QSNNLNR | [GAA](http://bindr.gdcb.iastate.edu:8080/ZiFDB/controller/searchFinger?target=GAA) | - | CoDA |   [ZF DNA Sequence](javascript:CoDAPopupArrayWindow("ZFA-unknown-2","RRAHLLN","DRGNLTR","QSNNLNR")) | |
| --- | --- | --- | --- | --- | --- | --- | --- | --- | --- | --- | --- | --- | --- | --- | --- | --- | --- | --- | --- | --- | --- | --- | --- | --- |
| [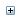](javascript:TreeView_ToggleNode(ctl00_ContentPlaceHolder1_tree12_Data,4,document.getElementById('ctl00_ContentPlaceHolder1_tree12n4'),'%20',document.getElementById('ctl00_ContentPlaceHolder1_tree12n4Nodes'))) | | ZFA-unknown-3 59 g[GATGCTGGA](http://bindr.gdcb.iastate.edu:8080/ZiFDB/controller/searchArray?site=GGAGCTGAT)a 69  59 cCTACGACCTt 69 | |  |

|  | 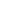 | | | FINGER | HELIX | TRIPLET | REFERENCE NUMBER | SOURCE | | --- | --- | --- | --- | --- | | F1 | RPAKLVL | [GGA](http://bindr.gdcb.iastate.edu:8080/ZiFDB/controller/searchFinger?target=GGA) | - | CoDA | | F2 | QRSDLTR | [GCT](http://bindr.gdcb.iastate.edu:8080/ZiFDB/controller/searchFinger?target=GCT) | - | CoDA | | F3 | LTHNLRR | [GAT](http://bindr.gdcb.iastate.edu:8080/ZiFDB/controller/searchFinger?target=GAT) | - | CoDA |   [ZF DNA Sequence](javascript:CoDAPopupArrayWindow("ZFA-unknown-3","RPAKLVL","QRSDLTR","LTHNLRR")) | |
| --- | --- | --- | --- | --- | --- | --- | --- | --- | --- | --- | --- | --- | --- | --- | --- | --- | --- | --- | --- | --- | --- | --- | --- | --- |
| [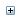](javascript:TreeView_ToggleNode(ctl00_ContentPlaceHolder1_tree12_Data,6,document.getElementById('ctl00_ContentPlaceHolder1_tree12n6'),'%20',document.getElementById('ctl00_ContentPlaceHolder1_tree12n6Nodes'))) | | ZFA-unknown-4 159 t[TGCGGGGCT](http://bindr.gdcb.iastate.edu:8080/ZiFDB/controller/searchArray?site=GCTGGGTGC)t 149  159 aACGCCCCGAa 149 | |  |

|  | 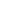 | | | FINGER | HELIX | TRIPLET | REFERENCE NUMBER | SOURCE | | --- | --- | --- | --- | --- | | F1 | THSMLAR | [GCT](http://bindr.gdcb.iastate.edu:8080/ZiFDB/controller/searchFinger?target=GCT) | - | CoDA | | F2 | RREHLVR | [GGG](http://bindr.gdcb.iastate.edu:8080/ZiFDB/controller/searchFinger?target=GGG) | - | CoDA | | F3 | ANRTLVH | [TGC](http://bindr.gdcb.iastate.edu:8080/ZiFDB/controller/searchFinger?target=TGC) | - | CoDA |   [ZF DNA Sequence](javascript:CoDAPopupArrayWindow("ZFA-unknown-4","THSMLAR","RREHLVR","ANRTLVH")) | |
| --- | --- | --- | --- | --- | --- | --- | --- | --- | --- | --- | --- | --- | --- | --- | --- | --- | --- | --- | --- | --- | --- | --- | --- | --- |
| [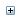](javascript:TreeView_ToggleNode(ctl00_ContentPlaceHolder1_tree12_Data,8,document.getElementById('ctl00_ContentPlaceHolder1_tree12n8'),'%20',document.getElementById('ctl00_ContentPlaceHolder1_tree12n8Nodes'))) | | ZFA-unknown-5 186 g[GCTGATGCC](http://bindr.gdcb.iastate.edu:8080/ZiFDB/controller/searchArray?site=GCCGATGCT)a 196  186 cCGACTACGGt 196 | |  |

|  | 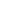 | | | FINGER | HELIX | TRIPLET | REFERENCE NUMBER | SOURCE | | --- | --- | --- | --- | --- | | F1 | DGSTLNR | [GCC](http://bindr.gdcb.iastate.edu:8080/ZiFDB/controller/searchFinger?target=GCC) | - | CoDA | | F2 | VRHNLTR | [GAT](http://bindr.gdcb.iastate.edu:8080/ZiFDB/controller/searchFinger?target=GAT) | - | CoDA | | F3 | LKHDLRR | [GCT](http://bindr.gdcb.iastate.edu:8080/ZiFDB/controller/searchFinger?target=GCT) | - | CoDA |   [ZF DNA Sequence](javascript:CoDAPopupArrayWindow("ZFA-unknown-5","DGSTLNR","VRHNLTR","LKHDLRR")) | |
| --- | --- | --- | --- | --- | --- | --- | --- | --- | --- | --- | --- | --- | --- | --- | --- | --- | --- | --- | --- | --- | --- | --- | --- | --- |
| [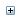](javascript:TreeView_ToggleNode(ctl00_ContentPlaceHolder1_tree12_Data,10,document.getElementById('ctl00_ContentPlaceHolder1_tree12n10'),'%20',document.getElementById('ctl00_ContentPlaceHolder1_tree12n10Nodes'))) | | ZFA-unknown-6 202 a[GCAGTTGGC](http://bindr.gdcb.iastate.edu:8080/ZiFDB/controller/searchArray?site=GGCGTTGCA)a 192  202 tCGTCAACCGt 192 | |  |

|  | 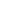 | | | FINGER | HELIX | TRIPLET | REFERENCE NUMBER | SOURCE | | --- | --- | --- | --- | --- | | F1 | APSKLKR | [GGC](http://bindr.gdcb.iastate.edu:8080/ZiFDB/controller/searchFinger?target=GGC) | - | CoDA | | F2 | HKSSLTR | [GTT](http://bindr.gdcb.iastate.edu:8080/ZiFDB/controller/searchFinger?target=GTT) | - | CoDA | | F3 | QGNTLTR | [GCA](http://bindr.gdcb.iastate.edu:8080/ZiFDB/controller/searchFinger?target=GCA) | - | CoDA |   [ZF DNA Sequence](javascript:CoDAPopupArrayWindow("ZFA-unknown-6","APSKLKR","HKSSLTR","QGNTLTR")) | |
| --- | --- | --- | --- | --- | --- | --- | --- | --- | --- | --- | --- | --- | --- | --- | --- | --- | --- | --- | --- | --- | --- | --- | --- | --- |
| [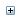](javascript:TreeView_ToggleNode(ctl00_ContentPlaceHolder1_tree12_Data,12,document.getElementById('ctl00_ContentPlaceHolder1_tree12n12'),'%20',document.getElementById('ctl00_ContentPlaceHolder1_tree12n12Nodes'))) | | ZFA-unknown-7 208 t[GGGGGAGCA](http://bindr.gdcb.iastate.edu:8080/ZiFDB/controller/searchArray?site=GCAGGAGGG)g 198  208 aCCCCCTCGTc 198 | |  |

|  | 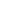 | | | FINGER | HELIX | TRIPLET | REFERENCE NUMBER | SOURCE | | --- | --- | --- | --- | --- | | F1 | KNTRLSV | [GCA](http://bindr.gdcb.iastate.edu:8080/ZiFDB/controller/searchFinger?target=GCA) | - | CoDA | | F2 | QSAHLKR | [GGA](http://bindr.gdcb.iastate.edu:8080/ZiFDB/controller/searchFinger?target=GGA) | - | CoDA | | F3 | RTEHLAR | [GGG](http://bindr.gdcb.iastate.edu:8080/ZiFDB/controller/searchFinger?target=GGG) | - | CoDA |   [ZF DNA Sequence](javascript:CoDAPopupArrayWindow("ZFA-unknown-7","KNTRLSV","QSAHLKR","RTEHLAR")) | |
| --- | --- | --- | --- | --- | --- | --- | --- | --- | --- | --- | --- | --- | --- | --- | --- | --- | --- | --- | --- | --- | --- | --- | --- | --- |
| [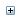](javascript:TreeView_ToggleNode(ctl00_ContentPlaceHolder1_tree12_Data,14,document.getElementById('ctl00_ContentPlaceHolder1_tree12n14'),'%20',document.getElementById('ctl00_ContentPlaceHolder1_tree12n14Nodes'))) | | ZFA-unknown-8 243 t[GCAGTTGGG](http://bindr.gdcb.iastate.edu:8080/ZiFDB/controller/searchArray?site=GGGGTTGCA)c 253  243 aCGTCAACCCg 253 | |  |

|  | 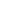 | | | FINGER | HELIX | TRIPLET | REFERENCE NUMBER | SOURCE | | --- | --- | --- | --- | --- | | F1 | KSNHLHV | [GGG](http://bindr.gdcb.iastate.edu:8080/ZiFDB/controller/searchFinger?target=GGG) | - | CoDA | | F2 | HKSSLTR | [GTT](http://bindr.gdcb.iastate.edu:8080/ZiFDB/controller/searchFinger?target=GTT) | - | CoDA | | F3 | QGNTLTR | [GCA](http://bindr.gdcb.iastate.edu:8080/ZiFDB/controller/searchFinger?target=GCA) | - | CoDA |   [ZF DNA Sequence](javascript:CoDAPopupArrayWindow("ZFA-unknown-8","KSNHLHV","HKSSLTR","QGNTLTR")) | |
| --- | --- | --- | --- | --- | --- | --- | --- | --- | --- | --- | --- | --- | --- | --- | --- | --- | --- | --- | --- | --- | --- | --- | --- | --- |
| [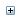](javascript:TreeView_ToggleNode(ctl00_ContentPlaceHolder1_tree12_Data,16,document.getElementById('ctl00_ContentPlaceHolder1_tree12n16'),'%20',document.getElementById('ctl00_ContentPlaceHolder1_tree12n16Nodes'))) | | ZFA-unknown-9 264 c[TCTGCTGCT](http://bindr.gdcb.iastate.edu:8080/ZiFDB/controller/searchArray?site=GCTGCTTCT)t 254  264 gAGACGACGAa 254 | |  |

|  | 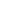 | | | FINGER | HELIX | TRIPLET | REFERENCE NUMBER | SOURCE | | --- | --- | --- | --- | --- | | F1 | MKNTLTR | [GCT](http://bindr.gdcb.iastate.edu:8080/ZiFDB/controller/searchFinger?target=GCT) | - | CoDA | | F2 | QRSDLTR | [GCT](http://bindr.gdcb.iastate.edu:8080/ZiFDB/controller/searchFinger?target=GCT) | - | CoDA | | F3 | QRNTLKG | [TCT](http://bindr.gdcb.iastate.edu:8080/ZiFDB/controller/searchFinger?target=TCT) | - | CoDA |   [ZF DNA Sequence](javascript:CoDAPopupArrayWindow("ZFA-unknown-9","MKNTLTR","QRSDLTR","QRNTLKG")) | |
| --- | --- | --- | --- | --- | --- | --- | --- | --- | --- | --- | --- | --- | --- | --- | --- | --- | --- | --- | --- | --- | --- | --- | --- | --- |
| [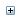](javascript:TreeView_ToggleNode(ctl00_ContentPlaceHolder1_tree12_Data,18,document.getElementById('ctl00_ContentPlaceHolder1_tree12n18'),'%20',document.getElementById('ctl00_ContentPlaceHolder1_tree12n18Nodes'))) | | ZFA-unknown-10 255 a[GCAGCAGAG](http://bindr.gdcb.iastate.edu:8080/ZiFDB/controller/searchArray?site=GAGGCAGCA)a 265  255 tCGTCGTCTCt 265 | |  |

|  | 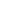 | | | FINGER | HELIX | TRIPLET | REFERENCE NUMBER | SOURCE | | --- | --- | --- | --- | --- | | F1 | KHSNLAR | [GAG](http://bindr.gdcb.iastate.edu:8080/ZiFDB/controller/searchFinger?target=GAG) | - | CoDA | | F2 | QSTTLKR | [GCA](http://bindr.gdcb.iastate.edu:8080/ZiFDB/controller/searchFinger?target=GCA) | - | CoDA | | F3 | QPNTLTR | [GCA](http://bindr.gdcb.iastate.edu:8080/ZiFDB/controller/searchFinger?target=GCA) | - | CoDA |   [ZF DNA Sequence](javascript:CoDAPopupArrayWindow("ZFA-unknown-10","KHSNLAR","QSTTLKR","QPNTLTR")) | |
| --- | --- | --- | --- | --- | --- | --- | --- | --- | --- | --- | --- | --- | --- | --- | --- | --- | --- | --- | --- | --- | --- | --- | --- | --- |
| [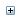](javascript:TreeView_ToggleNode(ctl00_ContentPlaceHolder1_tree12_Data,20,document.getElementById('ctl00_ContentPlaceHolder1_tree12n20'),'%20',document.getElementById('ctl00_ContentPlaceHolder1_tree12n20Nodes'))) | | ZFA-unknown-11 300 a[GGAGGTGAC](http://bindr.gdcb.iastate.edu:8080/ZiFDB/controller/searchArray?site=GACGGTGGA)a 310  300 tCCTCCACTGt 310 | |  |

|  | 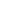 | | | FINGER | HELIX | TRIPLET | REFERENCE NUMBER | SOURCE | | --- | --- | --- | --- | --- | | F1 | EEANLRR | [GAC](http://bindr.gdcb.iastate.edu:8080/ZiFDB/controller/searchFinger?target=GAC) | - | CoDA | | F2 | EAHHLSR | [GGT](http://bindr.gdcb.iastate.edu:8080/ZiFDB/controller/searchFinger?target=GGT) | - | CoDA | | F3 | QNSHLRR | [GGA](http://bindr.gdcb.iastate.edu:8080/ZiFDB/controller/searchFinger?target=GGA) | - | CoDA |   [ZF DNA Sequence](javascript:CoDAPopupArrayWindow("ZFA-unknown-11","EEANLRR","EAHHLSR","QNSHLRR")) | |
| --- | --- | --- | --- | --- | --- | --- | --- | --- | --- | --- | --- | --- | --- | --- | --- | --- | --- | --- | --- | --- | --- | --- | --- | --- |
| [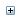](javascript:TreeView_ToggleNode(ctl00_ContentPlaceHolder1_tree12_Data,22,document.getElementById('ctl00_ContentPlaceHolder1_tree12n22'),'%20',document.getElementById('ctl00_ContentPlaceHolder1_tree12n22Nodes'))) | | ZFA-unknown-12 327 a[GGTGCAGCA](http://bindr.gdcb.iastate.edu:8080/ZiFDB/controller/searchArray?site=GCAGCAGGT)a 317  327 tCCACGTCGTt 317 | |  |

|  | 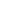 | | | FINGER | HELIX | TRIPLET | REFERENCE NUMBER | SOURCE | | --- | --- | --- | --- | --- | | F1 | QRGTLNR | [GCA](http://bindr.gdcb.iastate.edu:8080/ZiFDB/controller/searchFinger?target=GCA) | - | CoDA | | F2 | QSTTLKR | [GCA](http://bindr.gdcb.iastate.edu:8080/ZiFDB/controller/searchFinger?target=GCA) | - | CoDA | | F3 | VDHHLRR | [GGT](http://bindr.gdcb.iastate.edu:8080/ZiFDB/controller/searchFinger?target=GGT) | - | CoDA |   [ZF DNA Sequence](javascript:CoDAPopupArrayWindow("ZFA-unknown-12","QRGTLNR","QSTTLKR","VDHHLRR")) | |
| --- | --- | --- | --- | --- | --- | --- | --- | --- | --- | --- | --- | --- | --- | --- | --- | --- | --- | --- | --- | --- | --- | --- | --- | --- |
| [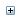](javascript:TreeView_ToggleNode(ctl00_ContentPlaceHolder1_tree12_Data,24,document.getElementById('ctl00_ContentPlaceHolder1_tree12n24'),'%20',document.getElementById('ctl00_ContentPlaceHolder1_tree12n24Nodes'))) | | ZFA-unknown-13 340 t[GGAGGAGAC](http://bindr.gdcb.iastate.edu:8080/ZiFDB/controller/searchArray?site=GACGGAGGA)c 350  340 aCCTCCTCTGg 350 | |  |

|  | 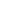 | | | FINGER | HELIX | TRIPLET | REFERENCE NUMBER | SOURCE | | --- | --- | --- | --- | --- | | F1 | DEANLRR | [GAC](http://bindr.gdcb.iastate.edu:8080/ZiFDB/controller/searchFinger?target=GAC) | - | CoDA | | F2 | QSAHLKR | [GGA](http://bindr.gdcb.iastate.edu:8080/ZiFDB/controller/searchFinger?target=GGA) | - | CoDA | | F3 | QMSHLKR | [GGA](http://bindr.gdcb.iastate.edu:8080/ZiFDB/controller/searchFinger?target=GGA) | - | CoDA |   [ZF DNA Sequence](javascript:CoDAPopupArrayWindow("ZFA-unknown-13","DEANLRR","QSAHLKR","QMSHLKR")) | |
| --- | --- | --- | --- | --- | --- | --- | --- | --- | --- | --- | --- | --- | --- | --- | --- | --- | --- | --- | --- | --- | --- | --- | --- | --- |
| [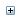](javascript:TreeView_ToggleNode(ctl00_ContentPlaceHolder1_tree12_Data,26,document.getElementById('ctl00_ContentPlaceHolder1_tree12n26'),'%20',document.getElementById('ctl00_ContentPlaceHolder1_tree12n26Nodes'))) | | ZFA-unknown-14 381 t[GTAGAAGTA](http://bindr.gdcb.iastate.edu:8080/ZiFDB/controller/searchArray?site=GTAGAAGTA)t 391  381 aCATCTTCATa 391 | |  |

|  | 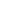 | | | FINGER | HELIX | TRIPLET | REFERENCE NUMBER | SOURCE | | --- | --- | --- | --- | --- | | F1 | QKQALDR | [GTA](http://bindr.gdcb.iastate.edu:8080/ZiFDB/controller/searchFinger?target=GTA) | - | CoDA | | F2 | QQTNLTR | [GAA](http://bindr.gdcb.iastate.edu:8080/ZiFDB/controller/searchFinger?target=GAA) | - | CoDA | | F3 | QSTSLQR | [GTA](http://bindr.gdcb.iastate.edu:8080/ZiFDB/controller/searchFinger?target=GTA) | - | CoDA |   [ZF DNA Sequence](javascript:CoDAPopupArrayWindow("ZFA-unknown-14","QKQALDR","QQTNLTR","QSTSLQR")) | |
| --- | --- | --- | --- | --- | --- | --- | --- | --- | --- | --- | --- | --- | --- | --- | --- | --- | --- | --- | --- | --- | --- | --- | --- | --- |
| [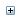](javascript:TreeView_ToggleNode(ctl00_ContentPlaceHolder1_tree12_Data,28,document.getElementById('ctl00_ContentPlaceHolder1_tree12n28'),'%20',document.getElementById('ctl00_ContentPlaceHolder1_tree12n28Nodes'))) | | ZFA-unknown-15 405 g[GCTGATGAT](http://bindr.gdcb.iastate.edu:8080/ZiFDB/controller/searchArray?site=GATGATGCT)t 415  405 cCGACTACTAa 415 | |  |

|  | 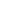 | | | FINGER | HELIX | TRIPLET | REFERENCE NUMBER | SOURCE | | --- | --- | --- | --- | --- | | F1 | TKQRLVV | [GAT](http://bindr.gdcb.iastate.edu:8080/ZiFDB/controller/searchFinger?target=GAT) | - | CoDA | | F2 | VRHNLTR | [GAT](http://bindr.gdcb.iastate.edu:8080/ZiFDB/controller/searchFinger?target=GAT) | - | CoDA | | F3 | LKHDLRR | [GCT](http://bindr.gdcb.iastate.edu:8080/ZiFDB/controller/searchFinger?target=GCT) | - | CoDA |   [ZF DNA Sequence](javascript:CoDAPopupArrayWindow("ZFA-unknown-15","TKQRLVV","VRHNLTR","LKHDLRR")) | |
| --- | --- | --- | --- | --- | --- | --- | --- | --- | --- | --- | --- | --- | --- | --- | --- | --- | --- | --- | --- | --- | --- | --- | --- | --- |
| [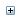](javascript:TreeView_ToggleNode(ctl00_ContentPlaceHolder1_tree12_Data,30,document.getElementById('ctl00_ContentPlaceHolder1_tree12n30'),'%20',document.getElementById('ctl00_ContentPlaceHolder1_tree12n30Nodes'))) | | ZFA-unknown-16 462 a[GTAGGAGGA](http://bindr.gdcb.iastate.edu:8080/ZiFDB/controller/searchArray?site=GGAGGAGTA)a 472  462 tCATCCTCCTt 472 | |  |

|  | 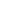 | | | FINGER | HELIX | TRIPLET | REFERENCE NUMBER | SOURCE | | --- | --- | --- | --- | --- | | F1 | RTDRLIR | [GGA](http://bindr.gdcb.iastate.edu:8080/ZiFDB/controller/searchFinger?target=GGA) | - | CoDA | | F2 | QSAHLKR | [GGA](http://bindr.gdcb.iastate.edu:8080/ZiFDB/controller/searchFinger?target=GGA) | - | CoDA | | F3 | QSTSLQR | [GTA](http://bindr.gdcb.iastate.edu:8080/ZiFDB/controller/searchFinger?target=GTA) | - | CoDA |   [ZF DNA Sequence](javascript:CoDAPopupArrayWindow("ZFA-unknown-16","RTDRLIR","QSAHLKR","QSTSLQR")) | |
| --- | --- | --- | --- | --- | --- | --- | --- | --- | --- | --- | --- | --- | --- | --- | --- | --- | --- | --- | --- | --- | --- | --- | --- | --- |
| [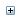](javascript:TreeView_ToggleNode(ctl00_ContentPlaceHolder1_tree12_Data,32,document.getElementById('ctl00_ContentPlaceHolder1_tree12n32'),'%20',document.getElementById('ctl00_ContentPlaceHolder1_tree12n32Nodes'))) | | ZFA-unknown-17 463 g[TAGGAGGAA](http://bindr.gdcb.iastate.edu:8080/ZiFDB/controller/searchArray?site=GAAGAGTAG)t 473  463 cATCCTCCTTa 473 | |  |

|  | 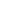 | | | FINGER | HELIX | TRIPLET | REFERENCE NUMBER | SOURCE | | --- | --- | --- | --- | --- | | F1 | QASNLLR | [GAA](http://bindr.gdcb.iastate.edu:8080/ZiFDB/controller/searchFinger?target=GAA) | - | CoDA | | F2 | RQDNLGR | [GAG](http://bindr.gdcb.iastate.edu:8080/ZiFDB/controller/searchFinger?target=GAG) | - | CoDA | | F3 | RPESLRP | [TAG](http://bindr.gdcb.iastate.edu:8080/ZiFDB/controller/searchFinger?target=TAG) | - | CoDA |   [ZF DNA Sequence](javascript:CoDAPopupArrayWindow("ZFA-unknown-17","QASNLLR","RQDNLGR","RPESLRP")) | |
| --- | --- | --- | --- | --- | --- | --- | --- | --- | --- | --- | --- | --- | --- | --- | --- | --- | --- | --- | --- | --- | --- | --- | --- | --- |
| [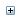](javascript:TreeView_ToggleNode(ctl00_ContentPlaceHolder1_tree12_Data,34,document.getElementById('ctl00_ContentPlaceHolder1_tree12n34'),'%20',document.getElementById('ctl00_ContentPlaceHolder1_tree12n34Nodes'))) | | ZFA-unknown-18 466 g[GAGGAATAG](http://bindr.gdcb.iastate.edu:8080/ZiFDB/controller/searchArray?site=TAGGAAGAG)g 476  466 cCTCCTTATCc 476 | |  |

|  | 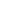 | | | FINGER | HELIX | TRIPLET | REFERENCE NUMBER | SOURCE | | --- | --- | --- | --- | --- | | F1 | RRRNLQI | [TAG](http://bindr.gdcb.iastate.edu:8080/ZiFDB/controller/searchFinger?target=TAG) | - | CoDA | | F2 | QQTNLTR | [GAA](http://bindr.gdcb.iastate.edu:8080/ZiFDB/controller/searchFinger?target=GAA) | - | CoDA | | F3 | RRDNLNR | [GAG](http://bindr.gdcb.iastate.edu:8080/ZiFDB/controller/searchFinger?target=GAG) | - | CoDA |   [ZF DNA Sequence](javascript:CoDAPopupArrayWindow("ZFA-unknown-18","RRRNLQI","QQTNLTR","RRDNLNR")) | |
| --- | --- | --- | --- | --- | --- | --- | --- | --- | --- | --- | --- | --- | --- | --- | --- | --- | --- | --- | --- | --- | --- | --- | --- | --- |
| [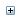](javascript:TreeView_ToggleNode(ctl00_ContentPlaceHolder1_tree12_Data,36,document.getElementById('ctl00_ContentPlaceHolder1_tree12n36'),'%20',document.getElementById('ctl00_ContentPlaceHolder1_tree12n36Nodes'))) | | ZFA-unknown-19 472 a[TAGGAGGTT](http://bindr.gdcb.iastate.edu:8080/ZiFDB/controller/searchArray?site=GTTGAGTAG)t 482  472 tATCCTCCAAa 482 | |  |

|  | 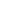 | | | FINGER | HELIX | TRIPLET | REFERENCE NUMBER | SOURCE | | --- | --- | --- | --- | --- | | F1 | TTTVLAR | [GTT](http://bindr.gdcb.iastate.edu:8080/ZiFDB/controller/searchFinger?target=GTT) | - | CoDA | | F2 | RQDNLGR | [GAG](http://bindr.gdcb.iastate.edu:8080/ZiFDB/controller/searchFinger?target=GAG) | - | CoDA | | F3 | RPESLRP | [TAG](http://bindr.gdcb.iastate.edu:8080/ZiFDB/controller/searchFinger?target=TAG) | - | CoDA |   [ZF DNA Sequence](javascript:CoDAPopupArrayWindow("ZFA-unknown-19","TTTVLAR","RQDNLGR","RPESLRP")) | |
| --- | --- | --- | --- | --- | --- | --- | --- | --- | --- | --- | --- | --- | --- | --- | --- | --- | --- | --- | --- | --- | --- | --- | --- | --- |
| [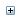](javascript:TreeView_ToggleNode(ctl00_ContentPlaceHolder1_tree12_Data,38,document.getElementById('ctl00_ContentPlaceHolder1_tree12n38'),'%20',document.getElementById('ctl00_ContentPlaceHolder1_tree12n38Nodes'))) | | ZFA-unknown-20 569 a[TCGGGGTGT](http://bindr.gdcb.iastate.edu:8080/ZiFDB/controller/searchArray?site=TGTGGGTCG)c 559  569 tAGCCCCACAg 559 | |  |

|  | 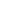 | | | FINGER | HELIX | TRIPLET | REFERENCE NUMBER | SOURCE | | --- | --- | --- | --- | --- | | F1 | RKQHLTL | [TGT](http://bindr.gdcb.iastate.edu:8080/ZiFDB/controller/searchFinger?target=TGT) | - | CoDA | | F2 | RREHLVR | [GGG](http://bindr.gdcb.iastate.edu:8080/ZiFDB/controller/searchFinger?target=GGG) | - | CoDA | | F3 | RMDSLGG | [TCG](http://bindr.gdcb.iastate.edu:8080/ZiFDB/controller/searchFinger?target=TCG) | - | CoDA |   [ZF DNA Sequence](javascript:CoDAPopupArrayWindow("ZFA-unknown-20","RKQHLTL","RREHLVR","RMDSLGG")) | |
| --- | --- | --- | --- | --- | --- | --- | --- | --- | --- | --- | --- | --- | --- | --- | --- | --- | --- | --- | --- | --- | --- | --- | --- | --- |
| [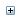](javascript:TreeView_ToggleNode(ctl00_ContentPlaceHolder1_tree12_Data,40,document.getElementById('ctl00_ContentPlaceHolder1_tree12n40'),'%20',document.getElementById('ctl00_ContentPlaceHolder1_tree12n40Nodes'))) | | ZFA-unknown-21 601 c[TGGGGATGT](http://bindr.gdcb.iastate.edu:8080/ZiFDB/controller/searchArray?site=TGTGGATGG)c 611  601 gACCCCTACAg 611 | |  |

|  | 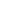 | | | FINGER | HELIX | TRIPLET | REFERENCE NUMBER | SOURCE | | --- | --- | --- | --- | --- | | F1 | RRQHLQY | [TGT](http://bindr.gdcb.iastate.edu:8080/ZiFDB/controller/searchFinger?target=TGT) | - | CoDA | | F2 | QSAHLKR | [GGA](http://bindr.gdcb.iastate.edu:8080/ZiFDB/controller/searchFinger?target=GGA) | - | CoDA | | F3 | RSDHLSL | [TGG](http://bindr.gdcb.iastate.edu:8080/ZiFDB/controller/searchFinger?target=TGG) | - | CoDA |   [ZF DNA Sequence](javascript:CoDAPopupArrayWindow("ZFA-unknown-21","RRQHLQY","QSAHLKR","RSDHLSL")) | |
| --- | --- | --- | --- | --- | --- | --- | --- | --- | --- | --- | --- | --- | --- | --- | --- | --- | --- | --- | --- | --- | --- | --- | --- | --- |
| [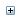](javascript:TreeView_ToggleNode(ctl00_ContentPlaceHolder1_tree12_Data,42,document.getElementById('ctl00_ContentPlaceHolder1_tree12n42'),'%20',document.getElementById('ctl00_ContentPlaceHolder1_tree12n42Nodes'))) | | ZFA-unknown-22 602 t[GGGGATGTC](http://bindr.gdcb.iastate.edu:8080/ZiFDB/controller/searchArray?site=GTCGATGGG)t 612  602 aCCCCTACAGa 612 | |  |

|  | 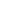 | | | FINGER | HELIX | TRIPLET | REFERENCE NUMBER | SOURCE | | --- | --- | --- | --- | --- | | F1 | TKKILTV | [GTC](http://bindr.gdcb.iastate.edu:8080/ZiFDB/controller/searchFinger?target=GTC) | - | CoDA | | F2 | VRHNLTR | [GAT](http://bindr.gdcb.iastate.edu:8080/ZiFDB/controller/searchFinger?target=GAT) | - | CoDA | | F3 | RGDKLGP | [GGG](http://bindr.gdcb.iastate.edu:8080/ZiFDB/controller/searchFinger?target=GGG) | - | CoDA |   [ZF DNA Sequence](javascript:CoDAPopupArrayWindow("ZFA-unknown-22","TKKILTV","VRHNLTR","RGDKLGP")) | |
| --- | --- | --- | --- | --- | --- | --- | --- | --- | --- | --- | --- | --- | --- | --- | --- | --- | --- | --- | --- | --- | --- | --- | --- | --- |
| [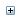](javascript:TreeView_ToggleNode(ctl00_ContentPlaceHolder1_tree12_Data,44,document.getElementById('ctl00_ContentPlaceHolder1_tree12n44'),'%20',document.getElementById('ctl00_ContentPlaceHolder1_tree12n44Nodes'))) | | ZFA-unknown-23 683 t[GAAGCAGTG](http://bindr.gdcb.iastate.edu:8080/ZiFDB/controller/searchArray?site=GTGGCAGAA)g 693  683 aCTTCGTCACc 693 | |  |

|  | 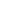 | | | FINGER | HELIX | TRIPLET | REFERENCE NUMBER | SOURCE | | --- | --- | --- | --- | --- | | F1 | RSHILTN | [GTG](http://bindr.gdcb.iastate.edu:8080/ZiFDB/controller/searchFinger?target=GTG) | - | CoDA | | F2 | QSTTLKR | [GCA](http://bindr.gdcb.iastate.edu:8080/ZiFDB/controller/searchFinger?target=GCA) | - | CoDA | | F3 | QRNNLGR | [GAA](http://bindr.gdcb.iastate.edu:8080/ZiFDB/controller/searchFinger?target=GAA) | - | CoDA |   [ZF DNA Sequence](javascript:CoDAPopupArrayWindow("ZFA-unknown-23","RSHILTN","QSTTLKR","QRNNLGR")) | |
| --- | --- | --- | --- | --- | --- | --- | --- | --- | --- | --- | --- | --- | --- | --- | --- | --- | --- | --- | --- | --- | --- | --- | --- | --- |
| [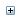](javascript:TreeView_ToggleNode(ctl00_ContentPlaceHolder1_tree12_Data,46,document.getElementById('ctl00_ContentPlaceHolder1_tree12n46'),'%20',document.getElementById('ctl00_ContentPlaceHolder1_tree12n46Nodes'))) | | ZFA-unknown-24 686 a[GCAGTGGCC](http://bindr.gdcb.iastate.edu:8080/ZiFDB/controller/searchArray?site=GCCGTGGCA)a 696  686 tCGTCACCGGt 696 | |  |

|  | 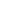 | | | FINGER | HELIX | TRIPLET | REFERENCE NUMBER | SOURCE | | --- | --- | --- | --- | --- | | F1 | KRRDLDR | [GCC](http://bindr.gdcb.iastate.edu:8080/ZiFDB/controller/searchFinger?target=GCC) | - | CoDA | | F2 | RREVLEN | [GTG](http://bindr.gdcb.iastate.edu:8080/ZiFDB/controller/searchFinger?target=GTG) | - | CoDA | | F3 | QGGTLRR | [GCA](http://bindr.gdcb.iastate.edu:8080/ZiFDB/controller/searchFinger?target=GCA) | - | CoDA |   [ZF DNA Sequence](javascript:CoDAPopupArrayWindow("ZFA-unknown-24","KRRDLDR","RREVLEN","QGGTLRR")) | |
| --- | --- | --- | --- | --- | --- | --- | --- | --- | --- | --- | --- | --- | --- | --- | --- | --- | --- | --- | --- | --- | --- | --- | --- | --- |
| [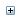](javascript:TreeView_ToggleNode(ctl00_ContentPlaceHolder1_tree12_Data,48,document.getElementById('ctl00_ContentPlaceHolder1_tree12n48'),'%20',document.getElementById('ctl00_ContentPlaceHolder1_tree12n48Nodes'))) | | ZFA-unknown-25 758 a[GTTGGAGGA](http://bindr.gdcb.iastate.edu:8080/ZiFDB/controller/searchArray?site=GGAGGAGTT)a 768  758 tCAACCTCCTt 768 | |  |

|  | 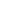 | | | FINGER | HELIX | TRIPLET | REFERENCE NUMBER | SOURCE | | --- | --- | --- | --- | --- | | F1 | RTDRLIR | [GGA](http://bindr.gdcb.iastate.edu:8080/ZiFDB/controller/searchFinger?target=GGA) | - | CoDA | | F2 | QSAHLKR | [GGA](http://bindr.gdcb.iastate.edu:8080/ZiFDB/controller/searchFinger?target=GGA) | - | CoDA | | F3 | HHNSLTR | [GTT](http://bindr.gdcb.iastate.edu:8080/ZiFDB/controller/searchFinger?target=GTT) | - | CoDA |   [ZF DNA Sequence](javascript:CoDAPopupArrayWindow("ZFA-unknown-25","RTDRLIR","QSAHLKR","HHNSLTR")) | |
| --- | --- | --- | --- | --- | --- | --- | --- | --- | --- | --- | --- | --- | --- | --- | --- | --- | --- | --- | --- | --- | --- | --- | --- | --- |
| [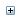](javascript:TreeView_ToggleNode(ctl00_ContentPlaceHolder1_tree12_Data,50,document.getElementById('ctl00_ContentPlaceHolder1_tree12n50'),'%20',document.getElementById('ctl00_ContentPlaceHolder1_tree12n50Nodes'))) | | ZFA-unknown-26 762 g[GAGGAAGCT](http://bindr.gdcb.iastate.edu:8080/ZiFDB/controller/searchArray?site=GCTGAAGAG)c 772  762 cCTCCTTCGAg 772 | |  |

|  | 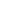 | | | FINGER | HELIX | TRIPLET | REFERENCE NUMBER | SOURCE | | --- | --- | --- | --- | --- | | F1 | QRQALDR | [GCT](http://bindr.gdcb.iastate.edu:8080/ZiFDB/controller/searchFinger?target=GCT) | - | CoDA | | F2 | QQTNLTR | [GAA](http://bindr.gdcb.iastate.edu:8080/ZiFDB/controller/searchFinger?target=GAA) | - | CoDA | | F3 | RRDNLNR | [GAG](http://bindr.gdcb.iastate.edu:8080/ZiFDB/controller/searchFinger?target=GAG) | - | CoDA |   [ZF DNA Sequence](javascript:CoDAPopupArrayWindow("ZFA-unknown-26","QRQALDR","QQTNLTR","RRDNLNR")) | |
| --- | --- | --- | --- | --- | --- | --- | --- | --- | --- | --- | --- | --- | --- | --- | --- | --- | --- | --- | --- | --- | --- | --- | --- | --- |
| [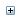](javascript:TreeView_ToggleNode(ctl00_ContentPlaceHolder1_tree12_Data,52,document.getElementById('ctl00_ContentPlaceHolder1_tree12n52'),'%20',document.getElementById('ctl00_ContentPlaceHolder1_tree12n52Nodes'))) | | ZFA-unknown-27 777 c[GGGGGAGCT](http://bindr.gdcb.iastate.edu:8080/ZiFDB/controller/searchArray?site=GCTGGAGGG)t 767  777 gCCCCCTCGAa 767 | |  |

|  | 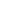 | | | FINGER | HELIX | TRIPLET | REFERENCE NUMBER | SOURCE | | --- | --- | --- | --- | --- | | F1 | TTQALRR | [GCT](http://bindr.gdcb.iastate.edu:8080/ZiFDB/controller/searchFinger?target=GCT) | - | CoDA | | F2 | QSAHLKR | [GGA](http://bindr.gdcb.iastate.edu:8080/ZiFDB/controller/searchFinger?target=GGA) | - | CoDA | | F3 | RTEHLAR | [GGG](http://bindr.gdcb.iastate.edu:8080/ZiFDB/controller/searchFinger?target=GGG) | - | CoDA |   [ZF DNA Sequence](javascript:CoDAPopupArrayWindow("ZFA-unknown-27","TTQALRR","QSAHLKR","RTEHLAR")) | |
| --- | --- | --- | --- | --- | --- | --- | --- | --- | --- | --- | --- | --- | --- | --- | --- | --- | --- | --- | --- | --- | --- | --- | --- | --- |
| [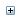](javascript:TreeView_ToggleNode(ctl00_ContentPlaceHolder1_tree12_Data,54,document.getElementById('ctl00_ContentPlaceHolder1_tree12n54'),'%20',document.getElementById('ctl00_ContentPlaceHolder1_tree12n54Nodes'))) | | ZFA-unknown-28 779 g[TCGGGGGAG](http://bindr.gdcb.iastate.edu:8080/ZiFDB/controller/searchArray?site=GAGGGGTCG)c 769  779 cAGCCCCCTCg 769 | |  |

|  | 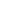 | | | FINGER | HELIX | TRIPLET | REFERENCE NUMBER | SOURCE | | --- | --- | --- | --- | --- | | F1 | RNTNLTR | [GAG](http://bindr.gdcb.iastate.edu:8080/ZiFDB/controller/searchFinger?target=GAG) | - | CoDA | | F2 | RREHLVR | [GGG](http://bindr.gdcb.iastate.edu:8080/ZiFDB/controller/searchFinger?target=GGG) | - | CoDA | | F3 | RMDSLGG | [TCG](http://bindr.gdcb.iastate.edu:8080/ZiFDB/controller/searchFinger?target=TCG) | - | CoDA |   [ZF DNA Sequence](javascript:CoDAPopupArrayWindow("ZFA-unknown-28","RNTNLTR","RREHLVR","RMDSLGG")) | |
| --- | --- | --- | --- | --- | --- | --- | --- | --- | --- | --- | --- | --- | --- | --- | --- | --- | --- | --- | --- | --- | --- | --- | --- | --- |
| [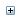](javascript:TreeView_ToggleNode(ctl00_ContentPlaceHolder1_tree12_Data,56,document.getElementById('ctl00_ContentPlaceHolder1_tree12n56'),'%20',document.getElementById('ctl00_ContentPlaceHolder1_tree12n56Nodes'))) | | ZFA-unknown-29 780 g[GTCGGGGGA](http://bindr.gdcb.iastate.edu:8080/ZiFDB/controller/searchArray?site=GGAGGGGTC)g 770  780 cCAGCCCCCTc 770 | |  |

|  | 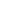 | | | FINGER | HELIX | TRIPLET | REFERENCE NUMBER | SOURCE | | --- | --- | --- | --- | --- | | F1 | TSAHLAR | [GGA](http://bindr.gdcb.iastate.edu:8080/ZiFDB/controller/searchFinger?target=GGA) | - | CoDA | | F2 | RREHLVR | [GGG](http://bindr.gdcb.iastate.edu:8080/ZiFDB/controller/searchFinger?target=GGG) | - | CoDA | | F3 | DPTSLNR | [GTC](http://bindr.gdcb.iastate.edu:8080/ZiFDB/controller/searchFinger?target=GTC) | - | CoDA |   [ZF DNA Sequence](javascript:CoDAPopupArrayWindow("ZFA-unknown-29","TSAHLAR","RREHLVR","DPTSLNR")) | |
| --- | --- | --- | --- | --- | --- | --- | --- | --- | --- | --- | --- | --- | --- | --- | --- | --- | --- | --- | --- | --- | --- | --- | --- | --- |
| [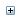](javascript:TreeView_ToggleNode(ctl00_ContentPlaceHolder1_tree12_Data,58,document.getElementById('ctl00_ContentPlaceHolder1_tree12n58'),'%20',document.getElementById('ctl00_ContentPlaceHolder1_tree12n58Nodes'))) | | ZFA-unknown-30 796 g[GGTGTTGTA](http://bindr.gdcb.iastate.edu:8080/ZiFDB/controller/searchArray?site=GTAGTTGGT)t 786  796 cCCACAACATa 786 | |  |

|  | 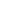 | | | FINGER | HELIX | TRIPLET | REFERENCE NUMBER | SOURCE | | --- | --- | --- | --- | --- | | F1 | QQQALKR | [GTA](http://bindr.gdcb.iastate.edu:8080/ZiFDB/controller/searchFinger?target=GTA) | - | CoDA | | F2 | HKSSLTR | [GTT](http://bindr.gdcb.iastate.edu:8080/ZiFDB/controller/searchFinger?target=GTT) | - | CoDA | | F3 | HGHRLKT | [GGT](http://bindr.gdcb.iastate.edu:8080/ZiFDB/controller/searchFinger?target=GGT) | - | CoDA |   [ZF DNA Sequence](javascript:CoDAPopupArrayWindow("ZFA-unknown-30","QQQALKR","HKSSLTR","HGHRLKT")) | |
| --- | --- | --- | --- | --- | --- | --- | --- | --- | --- | --- | --- | --- | --- | --- | --- | --- | --- | --- | --- | --- | --- | --- | --- | --- |
| [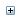](javascript:TreeView_ToggleNode(ctl00_ContentPlaceHolder1_tree12_Data,60,document.getElementById('ctl00_ContentPlaceHolder1_tree12n60'),'%20',document.getElementById('ctl00_ContentPlaceHolder1_tree12n60Nodes'))) | | ZFA-unknown-31 799 t[GGGGGTGTT](http://bindr.gdcb.iastate.edu:8080/ZiFDB/controller/searchArray?site=GTTGGTGGG)g 789  799 aCCCCCACAAc 789 | |  |

|  | 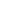 | | | FINGER | HELIX | TRIPLET | REFERENCE NUMBER | SOURCE | | --- | --- | --- | --- | --- | | F1 | AATALRR | [GTT](http://bindr.gdcb.iastate.edu:8080/ZiFDB/controller/searchFinger?target=GTT) | - | CoDA | | F2 | EAHHLSR | [GGT](http://bindr.gdcb.iastate.edu:8080/ZiFDB/controller/searchFinger?target=GGT) | - | CoDA | | F3 | RTEHLAR | [GGG](http://bindr.gdcb.iastate.edu:8080/ZiFDB/controller/searchFinger?target=GGG) | - | CoDA |   [ZF DNA Sequence](javascript:CoDAPopupArrayWindow("ZFA-unknown-31","AATALRR","EAHHLSR","RTEHLAR")) | |
| --- | --- | --- | --- | --- | --- | --- | --- | --- | --- | --- | --- | --- | --- | --- | --- | --- | --- | --- | --- | --- | --- | --- | --- | --- |
| [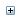](javascript:TreeView_ToggleNode(ctl00_ContentPlaceHolder1_tree12_Data,62,document.getElementById('ctl00_ContentPlaceHolder1_tree12n62'),'%20',document.getElementById('ctl00_ContentPlaceHolder1_tree12n62Nodes'))) | | ZFA-unknown-32 800 g[TGGGGGTGT](http://bindr.gdcb.iastate.edu:8080/ZiFDB/controller/searchArray?site=TGTGGGTGG)t 790  800 cACCCCCACAa 790 | |  |

|  | 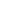 | | | FINGER | HELIX | TRIPLET | REFERENCE NUMBER | SOURCE | | --- | --- | --- | --- | --- | | F1 | RKQHLTL | [TGT](http://bindr.gdcb.iastate.edu:8080/ZiFDB/controller/searchFinger?target=TGT) | - | CoDA | | F2 | RREHLVR | [GGG](http://bindr.gdcb.iastate.edu:8080/ZiFDB/controller/searchFinger?target=GGG) | - | CoDA | | F3 | RMDHLAG | [TGG](http://bindr.gdcb.iastate.edu:8080/ZiFDB/controller/searchFinger?target=TGG) | - | CoDA |   [ZF DNA Sequence](javascript:CoDAPopupArrayWindow("ZFA-unknown-32","RKQHLTL","RREHLVR","RMDHLAG")) | |
| --- | --- | --- | --- | --- | --- | --- | --- | --- | --- | --- | --- | --- | --- | --- | --- | --- | --- | --- | --- | --- | --- | --- | --- | --- |
| [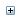](javascript:TreeView_ToggleNode(ctl00_ContentPlaceHolder1_tree12_Data,64,document.getElementById('ctl00_ContentPlaceHolder1_tree12n64'),'%20',document.getElementById('ctl00_ContentPlaceHolder1_tree12n64Nodes'))) | | ZFA-unknown-33 801 t[GTGGGGGTG](http://bindr.gdcb.iastate.edu:8080/ZiFDB/controller/searchArray?site=GTGGGGGTG)t 791  801 aCACCCCCACa 791 | |  |

|  | 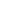 | | | FINGER | HELIX | TRIPLET | REFERENCE NUMBER | SOURCE | | --- | --- | --- | --- | --- | | F1 | SRFTLGR | [GTG](http://bindr.gdcb.iastate.edu:8080/ZiFDB/controller/searchFinger?target=GTG) | - | CoDA | | F2 | RREHLVR | [GGG](http://bindr.gdcb.iastate.edu:8080/ZiFDB/controller/searchFinger?target=GGG) | - | CoDA | | F3 | RPDALPR | [GTG](http://bindr.gdcb.iastate.edu:8080/ZiFDB/controller/searchFinger?target=GTG) | - | CoDA |   [ZF DNA Sequence](javascript:CoDAPopupArrayWindow("ZFA-unknown-33","SRFTLGR","RREHLVR","RPDALPR")) | |
| --- | --- | --- | --- | --- | --- | --- | --- | --- | --- | --- | --- | --- | --- | --- | --- | --- | --- | --- | --- | --- | --- | --- | --- | --- |
| [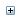](javascript:TreeView_ToggleNode(ctl00_ContentPlaceHolder1_tree12_Data,66,document.getElementById('ctl00_ContentPlaceHolder1_tree12n66'),'%20',document.getElementById('ctl00_ContentPlaceHolder1_tree12n66Nodes'))) | | ZFA-unknown-34 802 a[TGTGGGGGT](http://bindr.gdcb.iastate.edu:8080/ZiFDB/controller/searchArray?site=GGTGGGTGT)g 792  802 tACACCCCCAc 792 | |  |

|  | 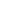 | | | FINGER | HELIX | TRIPLET | REFERENCE NUMBER | SOURCE | | --- | --- | --- | --- | --- | | F1 | RQSRLQR | [GGT](http://bindr.gdcb.iastate.edu:8080/ZiFDB/controller/searchFinger?target=GGT) | - | CoDA | | F2 | RREHLVR | [GGG](http://bindr.gdcb.iastate.edu:8080/ZiFDB/controller/searchFinger?target=GGG) | - | CoDA | | F3 | QRHGLSS | [TGT](http://bindr.gdcb.iastate.edu:8080/ZiFDB/controller/searchFinger?target=TGT) | - | CoDA |   [ZF DNA Sequence](javascript:CoDAPopupArrayWindow("ZFA-unknown-34","RQSRLQR","RREHLVR","QRHGLSS")) | |
| --- | --- | --- | --- | --- | --- | --- | --- | --- | --- | --- | --- | --- | --- | --- | --- | --- | --- | --- | --- | --- | --- | --- | --- | --- |
| [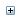](javascript:TreeView_ToggleNode(ctl00_ContentPlaceHolder1_tree12_Data,68,document.getElementById('ctl00_ContentPlaceHolder1_tree12n68'),'%20',document.getElementById('ctl00_ContentPlaceHolder1_tree12n68Nodes'))) | | ZFA-unknown-35 832 t[GGAGAATGC](http://bindr.gdcb.iastate.edu:8080/ZiFDB/controller/searchArray?site=TGCGAAGGA)t 842  832 aCCTCTTACGa 842 | |  |

|  | 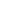 | | | FINGER | HELIX | TRIPLET | REFERENCE NUMBER | SOURCE | | --- | --- | --- | --- | --- | | F1 | RRRNLTL | [TGC](http://bindr.gdcb.iastate.edu:8080/ZiFDB/controller/searchFinger?target=TGC) | - | CoDA | | F2 | QQTNLTR | [GAA](http://bindr.gdcb.iastate.edu:8080/ZiFDB/controller/searchFinger?target=GAA) | - | CoDA | | F3 | QTTHLSR | [GGA](http://bindr.gdcb.iastate.edu:8080/ZiFDB/controller/searchFinger?target=GGA) | - | CoDA |   [ZF DNA Sequence](javascript:CoDAPopupArrayWindow("ZFA-unknown-35","RRRNLTL","QQTNLTR","QTTHLSR")) | |
| --- | --- | --- | --- | --- | --- | --- | --- | --- | --- | --- | --- | --- | --- | --- | --- | --- | --- | --- | --- | --- | --- | --- | --- | --- |
| [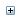](javascript:TreeView_ToggleNode(ctl00_ContentPlaceHolder1_tree12_Data,70,document.getElementById('ctl00_ContentPlaceHolder1_tree12n70'),'%20',document.getElementById('ctl00_ContentPlaceHolder1_tree12n70Nodes'))) | | ZFA-unknown-36 850 t[TTAGGGAAC](http://bindr.gdcb.iastate.edu:8080/ZiFDB/controller/searchArray?site=AACGGGTTA)t 860  850 aAATCCCTTGa 860 | |  |

|  | 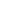 | | | FINGER | HELIX | TRIPLET | REFERENCE NUMBER | SOURCE | | --- | --- | --- | --- | --- | | F1 | HRTNLIA | [AAC](http://bindr.gdcb.iastate.edu:8080/ZiFDB/controller/searchFinger?target=AAC) | - | CoDA | | F2 | RREHLVR | [GGG](http://bindr.gdcb.iastate.edu:8080/ZiFDB/controller/searchFinger?target=GGG) | - | CoDA | | F3 | QQTGLNV | [TTA](http://bindr.gdcb.iastate.edu:8080/ZiFDB/controller/searchFinger?target=TTA) | - | CoDA |   [ZF DNA Sequence](javascript:CoDAPopupArrayWindow("ZFA-unknown-36","HRTNLIA","RREHLVR","QQTGLNV")) | |
| --- | --- | --- | --- | --- | --- | --- | --- | --- | --- | --- | --- | --- | --- | --- | --- | --- | --- | --- | --- | --- | --- | --- | --- | --- |
| [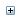](javascript:TreeView_ToggleNode(ctl00_ContentPlaceHolder1_tree12_Data,72,document.getElementById('ctl00_ContentPlaceHolder1_tree12n72'),'%20',document.getElementById('ctl00_ContentPlaceHolder1_tree12n72Nodes'))) | | ZFA-unknown-37 915 t[GCAGGGTGT](http://bindr.gdcb.iastate.edu:8080/ZiFDB/controller/searchArray?site=TGTGGGGCA)g 905  915 aCGTCCCACAc 905 | |  |

|  | 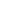 | | | FINGER | HELIX | TRIPLET | REFERENCE NUMBER | SOURCE | | --- | --- | --- | --- | --- | | F1 | RKQHLTL | [TGT](http://bindr.gdcb.iastate.edu:8080/ZiFDB/controller/searchFinger?target=TGT) | - | CoDA | | F2 | RREHLVR | [GGG](http://bindr.gdcb.iastate.edu:8080/ZiFDB/controller/searchFinger?target=GGG) | - | CoDA | | F3 | QTATLKR | [GCA](http://bindr.gdcb.iastate.edu:8080/ZiFDB/controller/searchFinger?target=GCA) | - | CoDA |   [ZF DNA Sequence](javascript:CoDAPopupArrayWindow("ZFA-unknown-37","RKQHLTL","RREHLVR","QTATLKR")) | |
| --- | --- | --- | --- | --- | --- | --- | --- | --- | --- | --- | --- | --- | --- | --- | --- | --- | --- | --- | --- | --- | --- | --- | --- | --- |
| [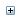](javascript:TreeView_ToggleNode(ctl00_ContentPlaceHolder1_tree12_Data,74,document.getElementById('ctl00_ContentPlaceHolder1_tree12n74'),'%20',document.getElementById('ctl00_ContentPlaceHolder1_tree12n74Nodes'))) | | ZFA-unknown-38 954 a[GGTGATGCA](http://bindr.gdcb.iastate.edu:8080/ZiFDB/controller/searchArray?site=GCAGATGGT)t 964  954 tCCACTACGTa 964 | |  |

|  | 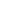 | | | FINGER | HELIX | TRIPLET | REFERENCE NUMBER | SOURCE | | --- | --- | --- | --- | --- | | F1 | LRHSLSR | [GCA](http://bindr.gdcb.iastate.edu:8080/ZiFDB/controller/searchFinger?target=GCA) | - | CoDA | | F2 | VRHNLTR | [GAT](http://bindr.gdcb.iastate.edu:8080/ZiFDB/controller/searchFinger?target=GAT) | - | CoDA | | F3 | QPHHLPR | [GGT](http://bindr.gdcb.iastate.edu:8080/ZiFDB/controller/searchFinger?target=GGT) | - | CoDA |   [ZF DNA Sequence](javascript:CoDAPopupArrayWindow("ZFA-unknown-38","LRHSLSR","VRHNLTR","QPHHLPR")) | |
| --- | --- | --- | --- | --- | --- | --- | --- | --- | --- | --- | --- | --- | --- | --- | --- | --- | --- | --- | --- | --- | --- | --- | --- | --- |
| [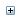](javascript:TreeView_ToggleNode(ctl00_ContentPlaceHolder1_tree12_Data,76,document.getElementById('ctl00_ContentPlaceHolder1_tree12n76'),'%20',document.getElementById('ctl00_ContentPlaceHolder1_tree12n76Nodes'))) | | ZFA-unknown-39 981 a[GATGAAGAA](http://bindr.gdcb.iastate.edu:8080/ZiFDB/controller/searchArray?site=GAAGAAGAT)t 991  981 tCTACTTCTTa 991 | |  |

|  | 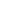 | | | FINGER | HELIX | TRIPLET | REFERENCE NUMBER | SOURCE | | --- | --- | --- | --- | --- | | F1 | QASNLTR | [GAA](http://bindr.gdcb.iastate.edu:8080/ZiFDB/controller/searchFinger?target=GAA) | - | CoDA | | F2 | QQTNLTR | [GAA](http://bindr.gdcb.iastate.edu:8080/ZiFDB/controller/searchFinger?target=GAA) | - | CoDA | | F3 | VGSNLTR | [GAT](http://bindr.gdcb.iastate.edu:8080/ZiFDB/controller/searchFinger?target=GAT) | - | CoDA |   [ZF DNA Sequence](javascript:CoDAPopupArrayWindow("ZFA-unknown-39","QASNLTR","QQTNLTR","VGSNLTR")) | |
| --- | --- | --- | --- | --- | --- | --- | --- | --- | --- | --- | --- | --- | --- | --- | --- | --- | --- | --- | --- | --- | --- | --- | --- | --- |
| [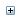](javascript:TreeView_ToggleNode(ctl00_ContentPlaceHolder1_tree12_Data,78,document.getElementById('ctl00_ContentPlaceHolder1_tree12n78'),'%20',document.getElementById('ctl00_ContentPlaceHolder1_tree12n78Nodes'))) | | ZFA-unknown-40 992 t[TAGGCAGTA](http://bindr.gdcb.iastate.edu:8080/ZiFDB/controller/searchArray?site=GTAGCATAG)c 1002  992 aATCCGTCATg 1002 | |  |

|  | 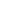 | | | FINGER | HELIX | TRIPLET | REFERENCE NUMBER | SOURCE | | --- | --- | --- | --- | --- | | F1 | QKQALTR | [GTA](http://bindr.gdcb.iastate.edu:8080/ZiFDB/controller/searchFinger?target=GTA) | - | CoDA | | F2 | QSTTLKR | [GCA](http://bindr.gdcb.iastate.edu:8080/ZiFDB/controller/searchFinger?target=GCA) | - | CoDA | | F3 | RRDGLAG | [TAG](http://bindr.gdcb.iastate.edu:8080/ZiFDB/controller/searchFinger?target=TAG) | - | CoDA |   [ZF DNA Sequence](javascript:CoDAPopupArrayWindow("ZFA-unknown-40","QKQALTR","QSTTLKR","RRDGLAG")) | |
| --- | --- | --- | --- | --- | --- | --- | --- | --- | --- | --- | --- | --- | --- | --- | --- | --- | --- | --- | --- | --- | --- | --- | --- | --- |
| [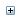](javascript:TreeView_ToggleNode(ctl00_ContentPlaceHolder1_tree12_Data,80,document.getElementById('ctl00_ContentPlaceHolder1_tree12n80'),'%20',document.getElementById('ctl00_ContentPlaceHolder1_tree12n80Nodes'))) | | ZFA-unknown-41 1073 t[GAGGCAGAA](http://bindr.gdcb.iastate.edu:8080/ZiFDB/controller/searchArray?site=GAAGCAGAG)c 1063  1073 aCTCCGTCTTg 1063 | |  |

|  | 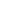 | | | FINGER | HELIX | TRIPLET | REFERENCE NUMBER | SOURCE | | --- | --- | --- | --- | --- | | F1 | QGSNLAR | [GAA](http://bindr.gdcb.iastate.edu:8080/ZiFDB/controller/searchFinger?target=GAA) | - | CoDA | | F2 | QSTTLKR | [GCA](http://bindr.gdcb.iastate.edu:8080/ZiFDB/controller/searchFinger?target=GCA) | - | CoDA | | F3 | RGDNLNR | [GAG](http://bindr.gdcb.iastate.edu:8080/ZiFDB/controller/searchFinger?target=GAG) | - | CoDA |   [ZF DNA Sequence](javascript:CoDAPopupArrayWindow("ZFA-unknown-41","QGSNLAR","QSTTLKR","RGDNLNR")) | |
| --- | --- | --- | --- | --- | --- | --- | --- | --- | --- | --- | --- | --- | --- | --- | --- | --- | --- | --- | --- | --- | --- | --- | --- | --- |
| [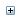](javascript:TreeView_ToggleNode(ctl00_ContentPlaceHolder1_tree12_Data,82,document.getElementById('ctl00_ContentPlaceHolder1_tree12n82'),'%20',document.getElementById('ctl00_ContentPlaceHolder1_tree12n82Nodes'))) | | ZFA-unknown-42 1075 g[GATGGAAGG](http://bindr.gdcb.iastate.edu:8080/ZiFDB/controller/searchArray?site=AGGGGAGAT)g 1085  1075 cCTACCTTCCc 1085 | |  |

|  | 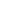 | | | FINGER | HELIX | TRIPLET | REFERENCE NUMBER | SOURCE | | --- | --- | --- | --- | --- | | F1 | RPHHLDA | [AGG](http://bindr.gdcb.iastate.edu:8080/ZiFDB/controller/searchFinger?target=AGG) | - | CoDA | | F2 | QSAHLKR | [GGA](http://bindr.gdcb.iastate.edu:8080/ZiFDB/controller/searchFinger?target=GGA) | - | CoDA | | F3 | ISHNLAR | [GAT](http://bindr.gdcb.iastate.edu:8080/ZiFDB/controller/searchFinger?target=GAT) | - | CoDA |   [ZF DNA Sequence](javascript:CoDAPopupArrayWindow("ZFA-unknown-42","RPHHLDA","QSAHLKR","ISHNLAR")) | |
| --- | --- | --- | --- | --- | --- | --- | --- | --- | --- | --- | --- | --- | --- | --- | --- | --- | --- | --- | --- | --- | --- | --- | --- | --- |
| [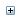](javascript:TreeView_ToggleNode(ctl00_ContentPlaceHolder1_tree12_Data,84,document.getElementById('ctl00_ContentPlaceHolder1_tree12n84'),'%20',document.getElementById('ctl00_ContentPlaceHolder1_tree12n84Nodes'))) | | ZFA-unknown-43 1079 g[GAAGGGGTC](http://bindr.gdcb.iastate.edu:8080/ZiFDB/controller/searchArray?site=GTCGGGGAA)a 1089  1079 cCTTCCCCAGt 1089 | |  |

|  | 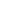 | | | FINGER | HELIX | TRIPLET | REFERENCE NUMBER | SOURCE | | --- | --- | --- | --- | --- | | F1 | TKSLLAR | [GTC](http://bindr.gdcb.iastate.edu:8080/ZiFDB/controller/searchFinger?target=GTC) | - | CoDA | | F2 | RREHLVR | [GGG](http://bindr.gdcb.iastate.edu:8080/ZiFDB/controller/searchFinger?target=GGG) | - | CoDA | | F3 | QDGNLGR | [GAA](http://bindr.gdcb.iastate.edu:8080/ZiFDB/controller/searchFinger?target=GAA) | - | CoDA |   [ZF DNA Sequence](javascript:CoDAPopupArrayWindow("ZFA-unknown-43","TKSLLAR","RREHLVR","QDGNLGR")) | |
| --- | --- | --- | --- | --- | --- | --- | --- | --- | --- | --- | --- | --- | --- | --- | --- | --- | --- | --- | --- | --- | --- | --- | --- | --- |
| [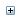](javascript:TreeView_ToggleNode(ctl00_ContentPlaceHolder1_tree12_Data,86,document.getElementById('ctl00_ContentPlaceHolder1_tree12n86'),'%20',document.getElementById('ctl00_ContentPlaceHolder1_tree12n86Nodes'))) | | ZFA-unknown-44 1095 g[GCTGGTGAC](http://bindr.gdcb.iastate.edu:8080/ZiFDB/controller/searchArray?site=GACGGTGCT)c 1085  1095 cCGACCACTGg 1085 | |  |

|  | 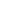 | | | FINGER | HELIX | TRIPLET | REFERENCE NUMBER | SOURCE | | --- | --- | --- | --- | --- | | F1 | EEANLRR | [GAC](http://bindr.gdcb.iastate.edu:8080/ZiFDB/controller/searchFinger?target=GAC) | - | CoDA | | F2 | EAHHLSR | [GGT](http://bindr.gdcb.iastate.edu:8080/ZiFDB/controller/searchFinger?target=GGT) | - | CoDA | | F3 | EGSGLKR | [GCT](http://bindr.gdcb.iastate.edu:8080/ZiFDB/controller/searchFinger?target=GCT) | - | CoDA |   [ZF DNA Sequence](javascript:CoDAPopupArrayWindow("ZFA-unknown-44","EEANLRR","EAHHLSR","EGSGLKR")) | |
| --- | --- | --- | --- | --- | --- | --- | --- | --- | --- | --- | --- | --- | --- | --- | --- | --- | --- | --- | --- | --- | --- | --- | --- | --- |
| [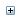](javascript:TreeView_ToggleNode(ctl00_ContentPlaceHolder1_tree12_Data,88,document.getElementById('ctl00_ContentPlaceHolder1_tree12n88'),'%20',document.getElementById('ctl00_ContentPlaceHolder1_tree12n88Nodes'))) | | ZFA-unknown-45 1102 g[GAAGATGGC](http://bindr.gdcb.iastate.edu:8080/ZiFDB/controller/searchArray?site=GGCGATGAA)t 1092  1102 cCTTCTACCGa 1092 | |  |

|  | 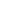 | | | FINGER | HELIX | TRIPLET | REFERENCE NUMBER | SOURCE | | --- | --- | --- | --- | --- | | F1 | SPSKLAR | [GGC](http://bindr.gdcb.iastate.edu:8080/ZiFDB/controller/searchFinger?target=GGC) | - | CoDA | | F2 | VRHNLTR | [GAT](http://bindr.gdcb.iastate.edu:8080/ZiFDB/controller/searchFinger?target=GAT) | - | CoDA | | F3 | QRNNLGR | [GAA](http://bindr.gdcb.iastate.edu:8080/ZiFDB/controller/searchFinger?target=GAA) | - | CoDA |   [ZF DNA Sequence](javascript:CoDAPopupArrayWindow("ZFA-unknown-45","SPSKLAR","VRHNLTR","QRNNLGR")) | |
| --- | --- | --- | --- | --- | --- | --- | --- | --- | --- | --- | --- | --- | --- | --- | --- | --- | --- | --- | --- | --- | --- | --- | --- | --- |
| [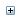](javascript:TreeView_ToggleNode(ctl00_ContentPlaceHolder1_tree12_Data,90,document.getElementById('ctl00_ContentPlaceHolder1_tree12n90'),'%20',document.getElementById('ctl00_ContentPlaceHolder1_tree12n90Nodes'))) | | ZFA-unknown-46 1186 t[TAAGATGTC](http://bindr.gdcb.iastate.edu:8080/ZiFDB/controller/searchArray?site=GTCGATTAA)a 1176  1186 aATTCTACAGt 1176 | |  |

|  | 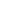 | | | FINGER | HELIX | TRIPLET | REFERENCE NUMBER | SOURCE | | --- | --- | --- | --- | --- | | F1 | TKKILTV | [GTC](http://bindr.gdcb.iastate.edu:8080/ZiFDB/controller/searchFinger?target=GTC) | - | CoDA | | F2 | VRHNLTR | [GAT](http://bindr.gdcb.iastate.edu:8080/ZiFDB/controller/searchFinger?target=GAT) | - | CoDA | | F3 | QQGNLQL | [TAA](http://bindr.gdcb.iastate.edu:8080/ZiFDB/controller/searchFinger?target=TAA) | - | CoDA |   [ZF DNA Sequence](javascript:CoDAPopupArrayWindow("ZFA-unknown-46","TKKILTV","VRHNLTR","QQGNLQL")) | |
| --- | --- | --- | --- | --- | --- | --- | --- | --- | --- | --- | --- | --- | --- | --- | --- | --- | --- | --- | --- | --- | --- | --- | --- | --- |
| [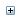](javascript:TreeView_ToggleNode(ctl00_ContentPlaceHolder1_tree12_Data,92,document.getElementById('ctl00_ContentPlaceHolder1_tree12n92'),'%20',document.getElementById('ctl00_ContentPlaceHolder1_tree12n92Nodes'))) | | ZFA-unknown-47 1269 g[GTAGAAAAC](http://bindr.gdcb.iastate.edu:8080/ZiFDB/controller/searchArray?site=AACGAAGTA)c 1259  1269 cCATCTTTTGg 1259 | |  |

|  | 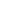 | | | FINGER | HELIX | TRIPLET | REFERENCE NUMBER | SOURCE | | --- | --- | --- | --- | --- | | F1 | GASALRQ | [AAC](http://bindr.gdcb.iastate.edu:8080/ZiFDB/controller/searchFinger?target=AAC) | - | CoDA | | F2 | QQTNLTR | [GAA](http://bindr.gdcb.iastate.edu:8080/ZiFDB/controller/searchFinger?target=GAA) | - | CoDA | | F3 | QSTSLQR | [GTA](http://bindr.gdcb.iastate.edu:8080/ZiFDB/controller/searchFinger?target=GTA) | - | CoDA |   [ZF DNA Sequence](javascript:CoDAPopupArrayWindow("ZFA-unknown-47","GASALRQ","QQTNLTR","QSTSLQR")) | |
| --- | --- | --- | --- | --- | --- | --- | --- | --- | --- | --- | --- | --- | --- | --- | --- | --- | --- | --- | --- | --- | --- | --- | --- | --- |
| [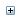](javascript:TreeView_ToggleNode(ctl00_ContentPlaceHolder1_tree12_Data,94,document.getElementById('ctl00_ContentPlaceHolder1_tree12n94'),'%20',document.getElementById('ctl00_ContentPlaceHolder1_tree12n94Nodes'))) | | ZFA-unknown-48 1272 t[GGGGTAGAA](http://bindr.gdcb.iastate.edu:8080/ZiFDB/controller/searchArray?site=GAAGTAGGG)a 1262  1272 aCCCCATCTTt 1262 | |  |

|  | 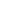 | | | FINGER | HELIX | TRIPLET | REFERENCE NUMBER | SOURCE | | --- | --- | --- | --- | --- | | F1 | HRPNLTR | [GAA](http://bindr.gdcb.iastate.edu:8080/ZiFDB/controller/searchFinger?target=GAA) | - | CoDA | | F2 | QRSSLVR | [GTA](http://bindr.gdcb.iastate.edu:8080/ZiFDB/controller/searchFinger?target=GTA) | - | CoDA | | F3 | RTEHLAR | [GGG](http://bindr.gdcb.iastate.edu:8080/ZiFDB/controller/searchFinger?target=GGG) | - | CoDA |   [ZF DNA Sequence](javascript:CoDAPopupArrayWindow("ZFA-unknown-48","HRPNLTR","QRSSLVR","RTEHLAR")) | |
| --- | --- | --- | --- | --- | --- | --- | --- | --- | --- | --- | --- | --- | --- | --- | --- | --- | --- | --- | --- | --- | --- | --- | --- | --- |
| [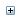](javascript:TreeView_ToggleNode(ctl00_ContentPlaceHolder1_tree12_Data,96,document.getElementById('ctl00_ContentPlaceHolder1_tree12n96'),'%20',document.getElementById('ctl00_ContentPlaceHolder1_tree12n96Nodes'))) | | ZFA-unknown-49 1307 g[GATGGGGTA](http://bindr.gdcb.iastate.edu:8080/ZiFDB/controller/searchArray?site=GTAGGGGAT)c 1317  1307 cCTACCCCATg 1317 | |  |

|  | 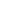 | | | FINGER | HELIX | TRIPLET | REFERENCE NUMBER | SOURCE | | --- | --- | --- | --- | --- | | F1 | QQSSLLR | [GTA](http://bindr.gdcb.iastate.edu:8080/ZiFDB/controller/searchFinger?target=GTA) | - | CoDA | | F2 | RREHLVR | [GGG](http://bindr.gdcb.iastate.edu:8080/ZiFDB/controller/searchFinger?target=GGG) | - | CoDA | | F3 | ISHNLAR | [GAT](http://bindr.gdcb.iastate.edu:8080/ZiFDB/controller/searchFinger?target=GAT) | - | CoDA |   [ZF DNA Sequence](javascript:CoDAPopupArrayWindow("ZFA-unknown-49","QQSSLLR","RREHLVR","ISHNLAR")) | |
| --- | --- | --- | --- | --- | --- | --- | --- | --- | --- | --- | --- | --- | --- | --- | --- | --- | --- | --- | --- | --- | --- | --- | --- | --- |
| [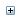](javascript:TreeView_ToggleNode(ctl00_ContentPlaceHolder1_tree12_Data,98,document.getElementById('ctl00_ContentPlaceHolder1_tree12n98'),'%20',document.getElementById('ctl00_ContentPlaceHolder1_tree12n98Nodes'))) | | ZFA-unknown-50 1309 a[TGGGGTACG](http://bindr.gdcb.iastate.edu:8080/ZiFDB/controller/searchArray?site=ACGGGTTGG)a 1319  1309 tACCCCATGCt 1319 | |  |

|  | 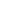 | | | FINGER | HELIX | TRIPLET | REFERENCE NUMBER | SOURCE | | --- | --- | --- | --- | --- | | F1 | KNNDLTR | [ACG](http://bindr.gdcb.iastate.edu:8080/ZiFDB/controller/searchFinger?target=ACG) | - | CoDA | | F2 | EAHHLSR | [GGT](http://bindr.gdcb.iastate.edu:8080/ZiFDB/controller/searchFinger?target=GGT) | - | CoDA | | F3 | RSDHLSL | [TGG](http://bindr.gdcb.iastate.edu:8080/ZiFDB/controller/searchFinger?target=TGG) | - | CoDA |   [ZF DNA Sequence](javascript:CoDAPopupArrayWindow("ZFA-unknown-50","KNNDLTR","EAHHLSR","RSDHLSL")) | |
| --- | --- | --- | --- | --- | --- | --- | --- | --- | --- | --- | --- | --- | --- | --- | --- | --- | --- | --- | --- | --- | --- | --- | --- | --- |
|  | | ZFA-unknown-51 1335 t[TTTGTTGGC](http://bindr.gdcb.iastate.edu:8080/ZiFDB/controller/searchArray?site=GGCGTTTTT)c 1325  1335 aAAACAACCGg 1325 | |  |

|  |  | | | FINGER | HELIX | TRIPLET | REFERENCE NUMBER | SOURCE | | --- | --- | --- | --- | --- | | F1 | APSKLKR | [GGC](http://bindr.gdcb.iastate.edu:8080/ZiFDB/controller/searchFinger?target=GGC) | - | CoDA | | F2 | HKSSLTR | [GTT](http://bindr.gdcb.iastate.edu:8080/ZiFDB/controller/searchFinger?target=GTT) | - | CoDA | | F3 | QRNALSG | [TTT](http://bindr.gdcb.iastate.edu:8080/ZiFDB/controller/searchFinger?target=TTT) | - | CoDA |   [ZF DNA Sequence](javascript:CoDAPopupArrayWindow("ZFA-unknown-51","APSKLKR","HKSSLTR","QRNALSG")) | |
| --- | --- | --- | --- | --- | --- | --- | --- | --- | --- | --- | --- | --- | --- | --- | --- | --- | --- | --- | --- | --- | --- | --- | --- | --- |
|  | | ZFA-unknown-52 1337 g[GAAGTTGCA](http://bindr.gdcb.iastate.edu:8080/ZiFDB/controller/searchArray?site=GCAGTTGAA)a 1347  1337 cCTTCAACGTt 1347 | |  |

|  |  | | | FINGER | HELIX | TRIPLET | REFERENCE NUMBER | SOURCE | | --- | --- | --- | --- | --- | | F1 | RRQELKR | [GCA](http://bindr.gdcb.iastate.edu:8080/ZiFDB/controller/searchFinger?target=GCA) | - | CoDA | | F2 | HKSSLTR | [GTT](http://bindr.gdcb.iastate.edu:8080/ZiFDB/controller/searchFinger?target=GTT) | - | CoDA | | F3 | QTNNLGR | [GAA](http://bindr.gdcb.iastate.edu:8080/ZiFDB/controller/searchFinger?target=GAA) | - | CoDA |   [ZF DNA Sequence](javascript:CoDAPopupArrayWindow("ZFA-unknown-52","RRQELKR","HKSSLTR","QTNNLGR")) | |
| --- | --- | --- | --- | --- | --- | --- | --- | --- | --- | --- | --- | --- | --- | --- | --- | --- | --- | --- | --- | --- | --- | --- | --- | --- |
|  | | ZFA-unknown-53 1401 a[GTAGGAGTA](http://bindr.gdcb.iastate.edu:8080/ZiFDB/controller/searchArray?site=GTAGGAGTA)t 1411  1401 tCATCCTCATa 1411 | |  |

|  |  | | | FINGER | HELIX | TRIPLET | REFERENCE NUMBER | SOURCE | | --- | --- | --- | --- | --- | | F1 | QQQALTR | [GTA](http://bindr.gdcb.iastate.edu:8080/ZiFDB/controller/searchFinger?target=GTA) | - | CoDA | | F2 | QSAHLKR | [GGA](http://bindr.gdcb.iastate.edu:8080/ZiFDB/controller/searchFinger?target=GGA) | - | CoDA | | F3 | QSTSLQR | [GTA](http://bindr.gdcb.iastate.edu:8080/ZiFDB/controller/searchFinger?target=GTA) | - | CoDA |   [ZF DNA Sequence](javascript:CoDAPopupArrayWindow("ZFA-unknown-53","QQQALTR","QSAHLKR","QSTSLQR")) | |
| --- | --- | --- | --- | --- | --- | --- | --- | --- | --- | --- | --- | --- | --- | --- | --- | --- | --- | --- | --- | --- | --- | --- | --- | --- |
|  | | ZFA-unknown-54 1416 t[TGGGCAGCT](http://bindr.gdcb.iastate.edu:8080/ZiFDB/controller/searchArray?site=GCTGCATGG)c 1426  1416 aACCCGTCGAg 1426 | |  |

|  |  | | | FINGER | HELIX | TRIPLET | REFERENCE NUMBER | SOURCE | | --- | --- | --- | --- | --- | | F1 | TKQILGR | [GCT](http://bindr.gdcb.iastate.edu:8080/ZiFDB/controller/searchFinger?target=GCT) | - | CoDA | | F2 | QSTTLKR | [GCA](http://bindr.gdcb.iastate.edu:8080/ZiFDB/controller/searchFinger?target=GCA) | - | CoDA | | F3 | RSDHLSL | [TGG](http://bindr.gdcb.iastate.edu:8080/ZiFDB/controller/searchFinger?target=TGG) | - | CoDA |   [ZF DNA Sequence](javascript:CoDAPopupArrayWindow("ZFA-unknown-54","TKQILGR","QSTTLKR","RSDHLSL")) | |
| --- | --- | --- | --- | --- | --- | --- | --- | --- | --- | --- | --- | --- | --- | --- | --- | --- | --- | --- | --- | --- | --- | --- | --- | --- |
|  | | ZFA-unknown-55 1491 a[GAGGAAGTT](http://bindr.gdcb.iastate.edu:8080/ZiFDB/controller/searchArray?site=GTTGAAGAG)c 1501  1491 tCTCCTTCAAg 1501 | |  |

|  |  | | | FINGER | HELIX | TRIPLET | REFERENCE NUMBER | SOURCE | | --- | --- | --- | --- | --- | | F1 | TSTLLKR | [GTT](http://bindr.gdcb.iastate.edu:8080/ZiFDB/controller/searchFinger?target=GTT) | - | CoDA | | F2 | QQTNLTR | [GAA](http://bindr.gdcb.iastate.edu:8080/ZiFDB/controller/searchFinger?target=GAA) | - | CoDA | | F3 | RRDNLNR | [GAG](http://bindr.gdcb.iastate.edu:8080/ZiFDB/controller/searchFinger?target=GAG) | - | CoDA |   [ZF DNA Sequence](javascript:CoDAPopupArrayWindow("ZFA-unknown-55","TSTLLKR","QQTNLTR","RRDNLNR")) | |
| --- | --- | --- | --- | --- | --- | --- | --- | --- | --- | --- | --- | --- | --- | --- | --- | --- | --- | --- | --- | --- | --- | --- | --- | --- |
|  | | ZFA-unknown-56 1515 g[GCAGAAGCA](http://bindr.gdcb.iastate.edu:8080/ZiFDB/controller/searchArray?site=GCAGAAGCA)g 1525  1515 cCGTCTTCGTc 1525 | |  |

|  |  | | | FINGER | HELIX | TRIPLET | REFERENCE NUMBER | SOURCE | | --- | --- | --- | --- | --- | | F1 | RGQELRR | [GCA](http://bindr.gdcb.iastate.edu:8080/ZiFDB/controller/searchFinger?target=GCA) | - | CoDA | | F2 | QQTNLTR | [GAA](http://bindr.gdcb.iastate.edu:8080/ZiFDB/controller/searchFinger?target=GAA) | - | CoDA | | F3 | QGNTLTR | [GCA](http://bindr.gdcb.iastate.edu:8080/ZiFDB/controller/searchFinger?target=GCA) | - | CoDA |   [ZF DNA Sequence](javascript:CoDAPopupArrayWindow("ZFA-unknown-56","RGQELRR","QQTNLTR","QGNTLTR")) | |
| --- | --- | --- | --- | --- | --- | --- | --- | --- | --- | --- | --- | --- | --- | --- | --- | --- | --- | --- | --- | --- | --- | --- | --- | --- |
|  | | ZFA-unknown-57 1518 a[GAAGCAGAA](http://bindr.gdcb.iastate.edu:8080/ZiFDB/controller/searchArray?site=GAAGCAGAA)t 1528  1518 tCTTCGTCTTa 1528 | |  |

|  |  | | | FINGER | HELIX | TRIPLET | REFERENCE NUMBER | SOURCE | | --- | --- | --- | --- | --- | | F1 | QGSNLAR | [GAA](http://bindr.gdcb.iastate.edu:8080/ZiFDB/controller/searchFinger?target=GAA) | - | CoDA | | F2 | QSTTLKR | [GCA](http://bindr.gdcb.iastate.edu:8080/ZiFDB/controller/searchFinger?target=GCA) | - | CoDA | | F3 | QRNNLGR | [GAA](http://bindr.gdcb.iastate.edu:8080/ZiFDB/controller/searchFinger?target=GAA) | - | CoDA |   [ZF DNA Sequence](javascript:CoDAPopupArrayWindow("ZFA-unknown-57","QGSNLAR","QSTTLKR","QRNNLGR")) | |
| --- | --- | --- | --- | --- | --- | --- | --- | --- | --- | --- | --- | --- | --- | --- | --- | --- | --- | --- | --- | --- | --- | --- | --- | --- |
|  | | ZFA-unknown-58 1556 t[GACTGAGAA](http://bindr.gdcb.iastate.edu:8080/ZiFDB/controller/searchArray?site=GAATGAGAC)t 1546  1556 aCTGACTCTTa 1546 | |  |

|  |  | | | FINGER | HELIX | TRIPLET | REFERENCE NUMBER | SOURCE | | --- | --- | --- | --- | --- | | F1 | QNANLKR | [GAA](http://bindr.gdcb.iastate.edu:8080/ZiFDB/controller/searchFinger?target=GAA) | - | CoDA | | F2 | QREHLTT | [TGA](http://bindr.gdcb.iastate.edu:8080/ZiFDB/controller/searchFinger?target=TGA) | - | CoDA | | F3 | DPSNLRR | [GAC](http://bindr.gdcb.iastate.edu:8080/ZiFDB/controller/searchFinger?target=GAC) | - | CoDA |   [ZF DNA Sequence](javascript:CoDAPopupArrayWindow("ZFA-unknown-58","QNANLKR","QREHLTT","DPSNLRR")) | |
| --- | --- | --- | --- | --- | --- | --- | --- | --- | --- | --- | --- | --- | --- | --- | --- | --- | --- | --- | --- | --- | --- | --- | --- | --- |
|  | | ZFA-unknown-59 1563 a[GAAGGATGT](http://bindr.gdcb.iastate.edu:8080/ZiFDB/controller/searchArray?site=TGTGGAGAA)t 1573  1563 tCTTCCTACAa 1573 | |  |

|  |  | | | FINGER | HELIX | TRIPLET | REFERENCE NUMBER | SOURCE | | --- | --- | --- | --- | --- | | F1 | RRQHLQY | [TGT](http://bindr.gdcb.iastate.edu:8080/ZiFDB/controller/searchFinger?target=TGT) | - | CoDA | | F2 | QSAHLKR | [GGA](http://bindr.gdcb.iastate.edu:8080/ZiFDB/controller/searchFinger?target=GGA) | - | CoDA | | F3 | LGENLRR | [GAA](http://bindr.gdcb.iastate.edu:8080/ZiFDB/controller/searchFinger?target=GAA) | - | CoDA |   [ZF DNA Sequence](javascript:CoDAPopupArrayWindow("ZFA-unknown-59","RRQHLQY","QSAHLKR","LGENLRR")) | |
| --- | --- | --- | --- | --- | --- | --- | --- | --- | --- | --- | --- | --- | --- | --- | --- | --- | --- | --- | --- | --- | --- | --- | --- | --- |
|  | | ZFA-unknown-60 1741 a[TAGGAAAGG](http://bindr.gdcb.iastate.edu:8080/ZiFDB/controller/searchArray?site=AGGGAATAG)a 1751  1741 tATCCTTTCCt 1751 | |  |

|  |  | | | FINGER | HELIX | TRIPLET | REFERENCE NUMBER | SOURCE | | --- | --- | --- | --- | --- | | F1 | RMAHLHA | [AGG](http://bindr.gdcb.iastate.edu:8080/ZiFDB/controller/searchFinger?target=AGG) | - | CoDA | | F2 | QQTNLTR | [GAA](http://bindr.gdcb.iastate.edu:8080/ZiFDB/controller/searchFinger?target=GAA) | - | CoDA | | F3 | RRDHLSL | [TAG](http://bindr.gdcb.iastate.edu:8080/ZiFDB/controller/searchFinger?target=TAG) | - | CoDA |   [ZF DNA Sequence](javascript:CoDAPopupArrayWindow("ZFA-unknown-60","RMAHLHA","QQTNLTR","RRDHLSL")) | |
| --- | --- | --- | --- | --- | --- | --- | --- | --- | --- | --- | --- | --- | --- | --- | --- | --- | --- | --- | --- | --- | --- | --- | --- | --- |
|  | | ZFA-unknown-61 1765 t[GGGGACAGG](http://bindr.gdcb.iastate.edu:8080/ZiFDB/controller/searchArray?site=AGGGACGGG)t 1775  1765 aCCCCTGTCCa 1775 | |  |

|  |  | | | FINGER | HELIX | TRIPLET | REFERENCE NUMBER | SOURCE | | --- | --- | --- | --- | --- | | F1 | RRAHLLN | [AGG](http://bindr.gdcb.iastate.edu:8080/ZiFDB/controller/searchFinger?target=AGG) | - | CoDA | | F2 | DRGNLTR | [GAC](http://bindr.gdcb.iastate.edu:8080/ZiFDB/controller/searchFinger?target=GAC) | - | CoDA | | F3 | RNHGLVR | [GGG](http://bindr.gdcb.iastate.edu:8080/ZiFDB/controller/searchFinger?target=GGG) | - | CoDA |   [ZF DNA Sequence](javascript:CoDAPopupArrayWindow("ZFA-unknown-61","RRAHLLN","DRGNLTR","RNHGLVR")) | |
| --- | --- | --- | --- | --- | --- | --- | --- | --- | --- | --- | --- | --- | --- | --- | --- | --- | --- | --- | --- | --- | --- | --- | --- | --- |
|  | | ZFA-unknown-62 1792 g[TAAGTGGAA](http://bindr.gdcb.iastate.edu:8080/ZiFDB/controller/searchArray?site=GAAGTGTAA)t 1782  1792 cATTCACCTTa 1782 | |  |

|  |  | | | FINGER | HELIX | TRIPLET | REFERENCE NUMBER | SOURCE | | --- | --- | --- | --- | --- | | F1 | HKPNLHR | [GAA](http://bindr.gdcb.iastate.edu:8080/ZiFDB/controller/searchFinger?target=GAA) | - | CoDA | | F2 | RREVLEN | [GTG](http://bindr.gdcb.iastate.edu:8080/ZiFDB/controller/searchFinger?target=GTG) | - | CoDA | | F3 | QRGNLNM | [TAA](http://bindr.gdcb.iastate.edu:8080/ZiFDB/controller/searchFinger?target=TAA) | - | CoDA |   [ZF DNA Sequence](javascript:CoDAPopupArrayWindow("ZFA-unknown-62","HKPNLHR","RREVLEN","QRGNLNM")) | |
| --- | --- | --- | --- | --- | --- | --- | --- | --- | --- | --- | --- | --- | --- | --- | --- | --- | --- | --- | --- | --- | --- | --- | --- | --- |
|  | | ZFA-unknown-63 1799 a[GAAGGATGT](http://bindr.gdcb.iastate.edu:8080/ZiFDB/controller/searchArray?site=TGTGGAGAA)a 1809  1799 tCTTCCTACAt 1809 | |  |

|  |  | | | FINGER | HELIX | TRIPLET | REFERENCE NUMBER | SOURCE | | --- | --- | --- | --- | --- | | F1 | RRQHLQY | [TGT](http://bindr.gdcb.iastate.edu:8080/ZiFDB/controller/searchFinger?target=TGT) | - | CoDA | | F2 | QSAHLKR | [GGA](http://bindr.gdcb.iastate.edu:8080/ZiFDB/controller/searchFinger?target=GGA) | - | CoDA | | F3 | LGENLRR | [GAA](http://bindr.gdcb.iastate.edu:8080/ZiFDB/controller/searchFinger?target=GAA) | - | CoDA |   [ZF DNA Sequence](javascript:CoDAPopupArrayWindow("ZFA-unknown-63","RRQHLQY","QSAHLKR","LGENLRR")) | |
| --- | --- | --- | --- | --- | --- | --- | --- | --- | --- | --- | --- | --- | --- | --- | --- | --- | --- | --- | --- | --- | --- | --- | --- | --- |
|  | | ZFA-unknown-64 1817 a[GTGGTGGAC](http://bindr.gdcb.iastate.edu:8080/ZiFDB/controller/searchArray?site=GACGTGGTG)a 1827  1817 tCACCACCTGt 1827 | |  |

|  |  | | | FINGER | HELIX | TRIPLET | REFERENCE NUMBER | SOURCE | | --- | --- | --- | --- | --- | | F1 | DEANLRR | [GAC](http://bindr.gdcb.iastate.edu:8080/ZiFDB/controller/searchFinger?target=GAC) | - | CoDA | | F2 | RREVLEN | [GTG](http://bindr.gdcb.iastate.edu:8080/ZiFDB/controller/searchFinger?target=GTG) | - | CoDA | | F3 | RKDALHV | [GTG](http://bindr.gdcb.iastate.edu:8080/ZiFDB/controller/searchFinger?target=GTG) | - | CoDA |   [ZF DNA Sequence](javascript:CoDAPopupArrayWindow("ZFA-unknown-64","DEANLRR","RREVLEN","RKDALHV")) | |
| --- | --- | --- | --- | --- | --- | --- | --- | --- | --- | --- | --- | --- | --- | --- | --- | --- | --- | --- | --- | --- | --- | --- | --- | --- |
|  | | ZFA-unknown-65 1835 g[GCAGGTAAC](http://bindr.gdcb.iastate.edu:8080/ZiFDB/controller/searchArray?site=AACGGTGCA)c 1845  1835 cCGTCCATTGg 1845 | |  |

|  |  | | | FINGER | HELIX | TRIPLET | REFERENCE NUMBER | SOURCE | | --- | --- | --- | --- | --- | | F1 | GASALRS | [AAC](http://bindr.gdcb.iastate.edu:8080/ZiFDB/controller/searchFinger?target=AAC) | - | CoDA | | F2 | EAHHLSR | [GGT](http://bindr.gdcb.iastate.edu:8080/ZiFDB/controller/searchFinger?target=GGT) | - | CoDA | | F3 | QNGTLTR | [GCA](http://bindr.gdcb.iastate.edu:8080/ZiFDB/controller/searchFinger?target=GCA) | - | CoDA |   [ZF DNA Sequence](javascript:CoDAPopupArrayWindow("ZFA-unknown-65","GASALRS","EAHHLSR","QNGTLTR")) | |
| --- | --- | --- | --- | --- | --- | --- | --- | --- | --- | --- | --- | --- | --- | --- | --- | --- | --- | --- | --- | --- | --- | --- | --- | --- |
|  | | ZFA-unknown-66 1878 t[GGTGTTGAG](http://bindr.gdcb.iastate.edu:8080/ZiFDB/controller/searchArray?site=GAGGTTGGT)a 1868  1878 aCCACAACTCt 1868 | |  |

|  |  | | | FINGER | HELIX | TRIPLET | REFERENCE NUMBER | SOURCE | | --- | --- | --- | --- | --- | | F1 | RPHNLLR | [GAG](http://bindr.gdcb.iastate.edu:8080/ZiFDB/controller/searchFinger?target=GAG) | - | CoDA | | F2 | HKSSLTR | [GTT](http://bindr.gdcb.iastate.edu:8080/ZiFDB/controller/searchFinger?target=GTT) | - | CoDA | | F3 | HGHRLKT | [GGT](http://bindr.gdcb.iastate.edu:8080/ZiFDB/controller/searchFinger?target=GGT) | - | CoDA |   [ZF DNA Sequence](javascript:CoDAPopupArrayWindow("ZFA-unknown-66","RPHNLLR","HKSSLTR","HGHRLKT")) | |
| --- | --- | --- | --- | --- | --- | --- | --- | --- | --- | --- | --- | --- | --- | --- | --- | --- | --- | --- | --- | --- | --- | --- | --- | --- |
|  | | ZFA-unknown-67 1881 c[GGTGGTGTT](http://bindr.gdcb.iastate.edu:8080/ZiFDB/controller/searchArray?site=GTTGGTGGT)g 1871  1881 gCCACCACAAc 1871 | |  |

|  |  | | | FINGER | HELIX | TRIPLET | REFERENCE NUMBER | SOURCE | | --- | --- | --- | --- | --- | | F1 | AATALRR | [GTT](http://bindr.gdcb.iastate.edu:8080/ZiFDB/controller/searchFinger?target=GTT) | - | CoDA | | F2 | EAHHLSR | [GGT](http://bindr.gdcb.iastate.edu:8080/ZiFDB/controller/searchFinger?target=GGT) | - | CoDA | | F3 | IRHHLKR | [GGT](http://bindr.gdcb.iastate.edu:8080/ZiFDB/controller/searchFinger?target=GGT) | - | CoDA |   [ZF DNA Sequence](javascript:CoDAPopupArrayWindow("ZFA-unknown-67","AATALRR","EAHHLSR","IRHHLKR")) | |
| --- | --- | --- | --- | --- | --- | --- | --- | --- | --- | --- | --- | --- | --- | --- | --- | --- | --- | --- | --- | --- | --- | --- | --- | --- |
|  | | ZFA-unknown-68 1883 a[GCGGTGGTG](http://bindr.gdcb.iastate.edu:8080/ZiFDB/controller/searchArray?site=GTGGTGGCG)t 1873  1883 tCGCCACCACa 1873 | |  |

|  |  | | | FINGER | HELIX | TRIPLET | REFERENCE NUMBER | SOURCE | | --- | --- | --- | --- | --- | | F1 | RNFVLAR | [GTG](http://bindr.gdcb.iastate.edu:8080/ZiFDB/controller/searchFinger?target=GTG) | - | CoDA | | F2 | RREVLEN | [GTG](http://bindr.gdcb.iastate.edu:8080/ZiFDB/controller/searchFinger?target=GTG) | - | CoDA | | F3 | RPDGLAR | [GCG](http://bindr.gdcb.iastate.edu:8080/ZiFDB/controller/searchFinger?target=GCG) | - | CoDA |   [ZF DNA Sequence](javascript:CoDAPopupArrayWindow("ZFA-unknown-68","RNFVLAR","RREVLEN","RPDGLAR")) | |
| --- | --- | --- | --- | --- | --- | --- | --- | --- | --- | --- | --- | --- | --- | --- | --- | --- | --- | --- | --- | --- | --- | --- | --- | --- |
|  | | ZFA-unknown-69 1885 g[TAAGATTAG](http://bindr.gdcb.iastate.edu:8080/ZiFDB/controller/searchArray?site=TAGGATTAA)t 1895  1885 cATTCTAATCa 1895 | |  |

|  |  | | | FINGER | HELIX | TRIPLET | REFERENCE NUMBER | SOURCE | | --- | --- | --- | --- | --- | | F1 | RSHNLRL | [TAG](http://bindr.gdcb.iastate.edu:8080/ZiFDB/controller/searchFinger?target=TAG) | - | CoDA | | F2 | VRHNLTR | [GAT](http://bindr.gdcb.iastate.edu:8080/ZiFDB/controller/searchFinger?target=GAT) | - | CoDA | | F3 | QQGNLQL | [TAA](http://bindr.gdcb.iastate.edu:8080/ZiFDB/controller/searchFinger?target=TAA) | - | CoDA |   [ZF DNA Sequence](javascript:CoDAPopupArrayWindow("ZFA-unknown-69","RSHNLRL","VRHNLTR","QQGNLQL")) | |
| --- | --- | --- | --- | --- | --- | --- | --- | --- | --- | --- | --- | --- | --- | --- | --- | --- | --- | --- | --- | --- | --- | --- | --- | --- |
|  | | ZFA-unknown-70 1920 a[GAGGGAGAA](http://bindr.gdcb.iastate.edu:8080/ZiFDB/controller/searchArray?site=GAAGGAGAG)g 1930  1920 tCTCCCTCTTc 1930 | |  |

|  |  | | | FINGER | HELIX | TRIPLET | REFERENCE NUMBER | SOURCE | | --- | --- | --- | --- | --- | | F1 | QRSNLAR | [GAA](http://bindr.gdcb.iastate.edu:8080/ZiFDB/controller/searchFinger?target=GAA) | - | CoDA | | F2 | QSAHLKR | [GGA](http://bindr.gdcb.iastate.edu:8080/ZiFDB/controller/searchFinger?target=GGA) | - | CoDA | | F3 | VHWNLMR | [GAG](http://bindr.gdcb.iastate.edu:8080/ZiFDB/controller/searchFinger?target=GAG) | - | CoDA |   [ZF DNA Sequence](javascript:CoDAPopupArrayWindow("ZFA-unknown-70","QRSNLAR","QSAHLKR","VHWNLMR")) | |
| --- | --- | --- | --- | --- | --- | --- | --- | --- | --- | --- | --- | --- | --- | --- | --- | --- | --- | --- | --- | --- | --- | --- | --- | --- |
|  | | ZFA-unknown-71 1923 g[GGAGAAGAA](http://bindr.gdcb.iastate.edu:8080/ZiFDB/controller/searchArray?site=GAAGAAGGA)a 1933  1923 cCCTCTTCTTt 1933 | |  |

|  |  | | | FINGER | HELIX | TRIPLET | REFERENCE NUMBER | SOURCE | | --- | --- | --- | --- | --- | | F1 | QASNLTR | [GAA](http://bindr.gdcb.iastate.edu:8080/ZiFDB/controller/searchFinger?target=GAA) | - | CoDA | | F2 | QQTNLTR | [GAA](http://bindr.gdcb.iastate.edu:8080/ZiFDB/controller/searchFinger?target=GAA) | - | CoDA | | F3 | QTTHLSR | [GGA](http://bindr.gdcb.iastate.edu:8080/ZiFDB/controller/searchFinger?target=GGA) | - | CoDA |   [ZF DNA Sequence](javascript:CoDAPopupArrayWindow("ZFA-unknown-71","QASNLTR","QQTNLTR","QTTHLSR")) | |
| --- | --- | --- | --- | --- | --- | --- | --- | --- | --- | --- | --- | --- | --- | --- | --- | --- | --- | --- | --- | --- | --- | --- | --- | --- |
|  | | ZFA-unknown-72 2037 a[GTAGTCTGT](http://bindr.gdcb.iastate.edu:8080/ZiFDB/controller/searchArray?site=TGTGTCGTA)t 2027  2037 tCATCAGACAa 2027 | |  |

|  |  | | | FINGER | HELIX | TRIPLET | REFERENCE NUMBER | SOURCE | | --- | --- | --- | --- | --- | | F1 | RKQHLVL | [TGT](http://bindr.gdcb.iastate.edu:8080/ZiFDB/controller/searchFinger?target=TGT) | - | CoDA | | F2 | DHSSLKR | [GTC](http://bindr.gdcb.iastate.edu:8080/ZiFDB/controller/searchFinger?target=GTC) | - | CoDA | | F3 | QSTSLQR | [GTA](http://bindr.gdcb.iastate.edu:8080/ZiFDB/controller/searchFinger?target=GTA) | - | CoDA |   [ZF DNA Sequence](javascript:CoDAPopupArrayWindow("ZFA-unknown-72","RKQHLVL","DHSSLKR","QSTSLQR")) | |
| --- | --- | --- | --- | --- | --- | --- | --- | --- | --- | --- | --- | --- | --- | --- | --- | --- | --- | --- | --- | --- | --- | --- | --- | --- |
|  | | ZFA-unknown-73 2045 t[GTTGATTAG](http://bindr.gdcb.iastate.edu:8080/ZiFDB/controller/searchArray?site=TAGGATGTT)t 2035  2045 aCAACTAATCa 2035 | |  |

|  |  | | | FINGER | HELIX | TRIPLET | REFERENCE NUMBER | SOURCE | | --- | --- | --- | --- | --- | | F1 | RSHNLRL | [TAG](http://bindr.gdcb.iastate.edu:8080/ZiFDB/controller/searchFinger?target=TAG) | - | CoDA | | F2 | VRHNLTR | [GAT](http://bindr.gdcb.iastate.edu:8080/ZiFDB/controller/searchFinger?target=GAT) | - | CoDA | | F3 | HHNSLTR | [GTT](http://bindr.gdcb.iastate.edu:8080/ZiFDB/controller/searchFinger?target=GTT) | - | CoDA |   [ZF DNA Sequence](javascript:CoDAPopupArrayWindow("ZFA-unknown-73","RSHNLRL","VRHNLTR","HHNSLTR")) | |
| --- | --- | --- | --- | --- | --- | --- | --- | --- | --- | --- | --- | --- | --- | --- | --- | --- | --- | --- | --- | --- | --- | --- | --- | --- |
|  | | ZFA-unknown-74 2052 t[TCTGCTTGT](http://bindr.gdcb.iastate.edu:8080/ZiFDB/controller/searchArray?site=TGTGCTTCT)t 2042  2052 aAGACGAACAa 2042 | |  |

|  |  | | | FINGER | HELIX | TRIPLET | REFERENCE NUMBER | SOURCE | | --- | --- | --- | --- | --- | | F1 | KRQHLEY | [TGT](http://bindr.gdcb.iastate.edu:8080/ZiFDB/controller/searchFinger?target=TGT) | - | CoDA | | F2 | QRSDLTR | [GCT](http://bindr.gdcb.iastate.edu:8080/ZiFDB/controller/searchFinger?target=GCT) | - | CoDA | | F3 | QRNTLKG | [TCT](http://bindr.gdcb.iastate.edu:8080/ZiFDB/controller/searchFinger?target=TCT) | - | CoDA |   [ZF DNA Sequence](javascript:CoDAPopupArrayWindow("ZFA-unknown-74","KRQHLEY","QRSDLTR","QRNTLKG")) | |
| --- | --- | --- | --- | --- | --- | --- | --- | --- | --- | --- | --- | --- | --- | --- | --- | --- | --- | --- | --- | --- | --- | --- | --- | --- |
|  | | ZFA-unknown-75 2235 g[TGTGCTGGT](http://bindr.gdcb.iastate.edu:8080/ZiFDB/controller/searchArray?site=GGTGCTTGT)a 2225  2235 cACACGACCAt 2225 | |  |

|  |  | | | FINGER | HELIX | TRIPLET | REFERENCE NUMBER | SOURCE | | --- | --- | --- | --- | --- | | F1 | RRQKLTI | [GGT](http://bindr.gdcb.iastate.edu:8080/ZiFDB/controller/searchFinger?target=GGT) | - | CoDA | | F2 | QRSDLTR | [GCT](http://bindr.gdcb.iastate.edu:8080/ZiFDB/controller/searchFinger?target=GCT) | - | CoDA | | F3 | QPHGLRH | [TGT](http://bindr.gdcb.iastate.edu:8080/ZiFDB/controller/searchFinger?target=TGT) | - | CoDA |   [ZF DNA Sequence](javascript:CoDAPopupArrayWindow("ZFA-unknown-75","RRQKLTI","QRSDLTR","QPHGLRH")) | |
| --- | --- | --- | --- | --- | --- | --- | --- | --- | --- | --- | --- | --- | --- | --- | --- | --- | --- | --- | --- | --- | --- | --- | --- | --- |
|  | | ZFA-unknown-76 2242 a[TAGGAGGAA](http://bindr.gdcb.iastate.edu:8080/ZiFDB/controller/searchArray?site=GAAGAGTAG)a 2252  2242 tATCCTCCTTt 2252 | |  |

|  |  | | | FINGER | HELIX | TRIPLET | REFERENCE NUMBER | SOURCE | | --- | --- | --- | --- | --- | | F1 | QASNLLR | [GAA](http://bindr.gdcb.iastate.edu:8080/ZiFDB/controller/searchFinger?target=GAA) | - | CoDA | | F2 | RQDNLGR | [GAG](http://bindr.gdcb.iastate.edu:8080/ZiFDB/controller/searchFinger?target=GAG) | - | CoDA | | F3 | RPESLRP | [TAG](http://bindr.gdcb.iastate.edu:8080/ZiFDB/controller/searchFinger?target=TAG) | - | CoDA |   [ZF DNA Sequence](javascript:CoDAPopupArrayWindow("ZFA-unknown-76","QASNLLR","RQDNLGR","RPESLRP")) | |
| --- | --- | --- | --- | --- | --- | --- | --- | --- | --- | --- | --- | --- | --- | --- | --- | --- | --- | --- | --- | --- | --- | --- | --- | --- |
|  | | ZFA-unknown-77 2244 a[GGAGGAAAC](http://bindr.gdcb.iastate.edu:8080/ZiFDB/controller/searchArray?site=AACGGAGGA)c 2254  2244 tCCTCCTTTGg 2254 | |  |

|  |  | | | FINGER | HELIX | TRIPLET | REFERENCE NUMBER | SOURCE | | --- | --- | --- | --- | --- | | F1 | GGTALVM | [AAC](http://bindr.gdcb.iastate.edu:8080/ZiFDB/controller/searchFinger?target=AAC) | - | CoDA | | F2 | QSAHLKR | [GGA](http://bindr.gdcb.iastate.edu:8080/ZiFDB/controller/searchFinger?target=GGA) | - | CoDA | | F3 | QMSHLKR | [GGA](http://bindr.gdcb.iastate.edu:8080/ZiFDB/controller/searchFinger?target=GGA) | - | CoDA |   [ZF DNA Sequence](javascript:CoDAPopupArrayWindow("ZFA-unknown-77","GGTALVM","QSAHLKR","QMSHLKR")) | |
| --- | --- | --- | --- | --- | --- | --- | --- | --- | --- | --- | --- | --- | --- | --- | --- | --- | --- | --- | --- | --- | --- | --- | --- | --- |
|  | | ZFA-unknown-78 2302 a[GAAGAGAAC](http://bindr.gdcb.iastate.edu:8080/ZiFDB/controller/searchArray?site=AACGAGGAA)t 2292  2302 tCTTCTCTTGa 2292 | |  |

|  |  | | | FINGER | HELIX | TRIPLET | REFERENCE NUMBER | SOURCE | | --- | --- | --- | --- | --- | | F1 | GHTALRN | [AAC](http://bindr.gdcb.iastate.edu:8080/ZiFDB/controller/searchFinger?target=AAC) | - | CoDA | | F2 | RQDNLGR | [GAG](http://bindr.gdcb.iastate.edu:8080/ZiFDB/controller/searchFinger?target=GAG) | - | CoDA | | F3 | QRNNLGR | [GAA](http://bindr.gdcb.iastate.edu:8080/ZiFDB/controller/searchFinger?target=GAA) | - | CoDA |   [ZF DNA Sequence](javascript:CoDAPopupArrayWindow("ZFA-unknown-78","GHTALRN","RQDNLGR","QRNNLGR")) | |
| --- | --- | --- | --- | --- | --- | --- | --- | --- | --- | --- | --- | --- | --- | --- | --- | --- | --- | --- | --- | --- | --- | --- | --- | --- |
|  | | ZFA-unknown-79 2325 t[TGTGCTGGC](http://bindr.gdcb.iastate.edu:8080/ZiFDB/controller/searchArray?site=GGCGCTTGT)t 2315  2325 aACACGACCGa 2315 | |  |

|  |  | | | FINGER | HELIX | TRIPLET | REFERENCE NUMBER | SOURCE | | --- | --- | --- | --- | --- | | F1 | APSKLAR | [GGC](http://bindr.gdcb.iastate.edu:8080/ZiFDB/controller/searchFinger?target=GGC) | - | CoDA | | F2 | QRSDLTR | [GCT](http://bindr.gdcb.iastate.edu:8080/ZiFDB/controller/searchFinger?target=GCT) | - | CoDA | | F3 | QPHGLRH | [TGT](http://bindr.gdcb.iastate.edu:8080/ZiFDB/controller/searchFinger?target=TGT) | - | CoDA |   [ZF DNA Sequence](javascript:CoDAPopupArrayWindow("ZFA-unknown-79","APSKLAR","QRSDLTR","QPHGLRH")) | |
| --- | --- | --- | --- | --- | --- | --- | --- | --- | --- | --- | --- | --- | --- | --- | --- | --- | --- | --- | --- | --- | --- | --- | --- | --- |
|  | | ZFA-unknown-80 2436 a[GGAGAGGCT](http://bindr.gdcb.iastate.edu:8080/ZiFDB/controller/searchArray?site=GCTGAGGGA)a 2446  2436 tCCTCTCCGAt 2446 | |  |

|  |  | | | FINGER | HELIX | TRIPLET | REFERENCE NUMBER | SOURCE | | --- | --- | --- | --- | --- | | F1 | NKQALDR | [GCT](http://bindr.gdcb.iastate.edu:8080/ZiFDB/controller/searchFinger?target=GCT) | - | CoDA | | F2 | RQDNLGR | [GAG](http://bindr.gdcb.iastate.edu:8080/ZiFDB/controller/searchFinger?target=GAG) | - | CoDA | | F3 | QANHLSR | [GGA](http://bindr.gdcb.iastate.edu:8080/ZiFDB/controller/searchFinger?target=GGA) | - | CoDA |   [ZF DNA Sequence](javascript:CoDAPopupArrayWindow("ZFA-unknown-80","NKQALDR","RQDNLGR","QANHLSR")) | |
| --- | --- | --- | --- | --- | --- | --- | --- | --- | --- | --- | --- | --- | --- | --- | --- | --- | --- | --- | --- | --- | --- | --- | --- | --- |
|  | | ZFA-unknown-81 2450 a[TGGGCAGGC](http://bindr.gdcb.iastate.edu:8080/ZiFDB/controller/searchArray?site=GGCGCATGG)a 2460  2450 tACCCGTCCGt 2460 | |  |

|  |  | | | FINGER | HELIX | TRIPLET | REFERENCE NUMBER | SOURCE | | --- | --- | --- | --- | --- | | F1 | VPSKLLR | [GGC](http://bindr.gdcb.iastate.edu:8080/ZiFDB/controller/searchFinger?target=GGC) | - | CoDA | | F2 | QSTTLKR | [GCA](http://bindr.gdcb.iastate.edu:8080/ZiFDB/controller/searchFinger?target=GCA) | - | CoDA | | F3 | RSDHLSL | [TGG](http://bindr.gdcb.iastate.edu:8080/ZiFDB/controller/searchFinger?target=TGG) | - | CoDA |   [ZF DNA Sequence](javascript:CoDAPopupArrayWindow("ZFA-unknown-81","VPSKLLR","QSTTLKR","RSDHLSL")) | |
| --- | --- | --- | --- | --- | --- | --- | --- | --- | --- | --- | --- | --- | --- | --- | --- | --- | --- | --- | --- | --- | --- | --- | --- | --- |
|  | | ZFA-unknown-82 2520 a[GTTGCAGTA](http://bindr.gdcb.iastate.edu:8080/ZiFDB/controller/searchArray?site=GTAGCAGTT)c 2530  2520 tCAACGTCATg 2530 | |  |

|  |  | | | FINGER | HELIX | TRIPLET | REFERENCE NUMBER | SOURCE | | --- | --- | --- | --- | --- | | F1 | QKQALTR | [GTA](http://bindr.gdcb.iastate.edu:8080/ZiFDB/controller/searchFinger?target=GTA) | - | CoDA | | F2 | QSTTLKR | [GCA](http://bindr.gdcb.iastate.edu:8080/ZiFDB/controller/searchFinger?target=GCA) | - | CoDA | | F3 | TRHSLGR | [GTT](http://bindr.gdcb.iastate.edu:8080/ZiFDB/controller/searchFinger?target=GTT) | - | CoDA |   [ZF DNA Sequence](javascript:CoDAPopupArrayWindow("ZFA-unknown-82","QKQALTR","QSTTLKR","TRHSLGR")) | |
| --- | --- | --- | --- | --- | --- | --- | --- | --- | --- | --- | --- | --- | --- | --- | --- | --- | --- | --- | --- | --- | --- | --- | --- | --- |
|  | | ZFA-unknown-83 2550 a[GAAGCAGAG](http://bindr.gdcb.iastate.edu:8080/ZiFDB/controller/searchArray?site=GAGGCAGAA)g 2560  2550 tCTTCGTCTCc 2560 | |  |

|  |  | | | FINGER | HELIX | TRIPLET | REFERENCE NUMBER | SOURCE | | --- | --- | --- | --- | --- | | F1 | KHSNLAR | [GAG](http://bindr.gdcb.iastate.edu:8080/ZiFDB/controller/searchFinger?target=GAG) | - | CoDA | | F2 | QSTTLKR | [GCA](http://bindr.gdcb.iastate.edu:8080/ZiFDB/controller/searchFinger?target=GCA) | - | CoDA | | F3 | QRNNLGR | [GAA](http://bindr.gdcb.iastate.edu:8080/ZiFDB/controller/searchFinger?target=GAA) | - | CoDA |   [ZF DNA Sequence](javascript:CoDAPopupArrayWindow("ZFA-unknown-83","KHSNLAR","QSTTLKR","QRNNLGR")) | |
| --- | --- | --- | --- | --- | --- | --- | --- | --- | --- | --- | --- | --- | --- | --- | --- | --- | --- | --- | --- | --- | --- | --- | --- | --- |
|  | | ZFA-unknown-84 2553 a[GCAGAGGTA](http://bindr.gdcb.iastate.edu:8080/ZiFDB/controller/searchArray?site=GTAGAGGCA)a 2563  2553 tCGTCTCCATt 2563 | |  |

|  |  | | | FINGER | HELIX | TRIPLET | REFERENCE NUMBER | SOURCE | | --- | --- | --- | --- | --- | | F1 | QQQALVR | [GTA](http://bindr.gdcb.iastate.edu:8080/ZiFDB/controller/searchFinger?target=GTA) | - | CoDA | | F2 | RQDNLGR | [GAG](http://bindr.gdcb.iastate.edu:8080/ZiFDB/controller/searchFinger?target=GAG) | - | CoDA | | F3 | QSNVLSR | [GCA](http://bindr.gdcb.iastate.edu:8080/ZiFDB/controller/searchFinger?target=GCA) | - | CoDA |   [ZF DNA Sequence](javascript:CoDAPopupArrayWindow("ZFA-unknown-84","QQQALVR","RQDNLGR","QSNVLSR")) | |
| --- | --- | --- | --- | --- | --- | --- | --- | --- | --- | --- | --- | --- | --- | --- | --- | --- | --- | --- | --- | --- | --- | --- | --- | --- |
|  | | ZFA-unknown-85 2592 t[GCTGTCTGT](http://bindr.gdcb.iastate.edu:8080/ZiFDB/controller/searchArray?site=TGTGTCGCT)c 2582  2592 aCGACAGACAg 2582 | |  |

|  |  | | | FINGER | HELIX | TRIPLET | REFERENCE NUMBER | SOURCE | | --- | --- | --- | --- | --- | | F1 | RKQHLVL | [TGT](http://bindr.gdcb.iastate.edu:8080/ZiFDB/controller/searchFinger?target=TGT) | - | CoDA | | F2 | DHSSLKR | [GTC](http://bindr.gdcb.iastate.edu:8080/ZiFDB/controller/searchFinger?target=GTC) | - | CoDA | | F3 | VSNSLAR | [GCT](http://bindr.gdcb.iastate.edu:8080/ZiFDB/controller/searchFinger?target=GCT) | - | CoDA |   [ZF DNA Sequence](javascript:CoDAPopupArrayWindow("ZFA-unknown-85","RKQHLVL","DHSSLKR","VSNSLAR")) | |
| --- | --- | --- | --- | --- | --- | --- | --- | --- | --- | --- | --- | --- | --- | --- | --- | --- | --- | --- | --- | --- | --- | --- | --- | --- |
|  | | ZFA-unknown-86 2614 g[GCAGATGGC](http://bindr.gdcb.iastate.edu:8080/ZiFDB/controller/searchArray?site=GGCGATGCA)c 2624  2614 cCGTCTACCGg 2624 | |  |

|  |  | | | FINGER | HELIX | TRIPLET | REFERENCE NUMBER | SOURCE | | --- | --- | --- | --- | --- | | F1 | SPSKLAR | [GGC](http://bindr.gdcb.iastate.edu:8080/ZiFDB/controller/searchFinger?target=GGC) | - | CoDA | | F2 | VRHNLTR | [GAT](http://bindr.gdcb.iastate.edu:8080/ZiFDB/controller/searchFinger?target=GAT) | - | CoDA | | F3 | QGNTLTR | [GCA](http://bindr.gdcb.iastate.edu:8080/ZiFDB/controller/searchFinger?target=GCA) | - | CoDA |   [ZF DNA Sequence](javascript:CoDAPopupArrayWindow("ZFA-unknown-86","SPSKLAR","VRHNLTR","QGNTLTR")) | |
| --- | --- | --- | --- | --- | --- | --- | --- | --- | --- | --- | --- | --- | --- | --- | --- | --- | --- | --- | --- | --- | --- | --- | --- | --- |
|  | | ZFA-unknown-87 2639 t[GTAGATGTG](http://bindr.gdcb.iastate.edu:8080/ZiFDB/controller/searchArray?site=GTGGATGTA)t 2629  2639 aCATCTACACa 2629 | |  |

|  |  | | | FINGER | HELIX | TRIPLET | REFERENCE NUMBER | SOURCE | | --- | --- | --- | --- | --- | | F1 | RKHILIH | [GTG](http://bindr.gdcb.iastate.edu:8080/ZiFDB/controller/searchFinger?target=GTG) | - | CoDA | | F2 | VRHNLTR | [GAT](http://bindr.gdcb.iastate.edu:8080/ZiFDB/controller/searchFinger?target=GAT) | - | CoDA | | F3 | QSTSLQR | [GTA](http://bindr.gdcb.iastate.edu:8080/ZiFDB/controller/searchFinger?target=GTA) | - | CoDA |   [ZF DNA Sequence](javascript:CoDAPopupArrayWindow("ZFA-unknown-87","RKHILIH","VRHNLTR","QSTSLQR")) | |
| --- | --- | --- | --- | --- | --- | --- | --- | --- | --- | --- | --- | --- | --- | --- | --- | --- | --- | --- | --- | --- | --- | --- | --- | --- |
|  | | ZFA-unknown-88 2642 g[TGTGTAGAT](http://bindr.gdcb.iastate.edu:8080/ZiFDB/controller/searchArray?site=GATGTATGT)g 2632  2642 cACACATCTAc 2632 | |  |

|  |  | | | FINGER | HELIX | TRIPLET | REFERENCE NUMBER | SOURCE | | --- | --- | --- | --- | --- | | F1 | TRQRLAI | [GAT](http://bindr.gdcb.iastate.edu:8080/ZiFDB/controller/searchFinger?target=GAT) | - | CoDA | | F2 | QRSSLVR | [GTA](http://bindr.gdcb.iastate.edu:8080/ZiFDB/controller/searchFinger?target=GTA) | - | CoDA | | F3 | QPHGLAH | [TGT](http://bindr.gdcb.iastate.edu:8080/ZiFDB/controller/searchFinger?target=TGT) | - | CoDA |   [ZF DNA Sequence](javascript:CoDAPopupArrayWindow("ZFA-unknown-88","TRQRLAI","QRSSLVR","QPHGLAH")) | |
| --- | --- | --- | --- | --- | --- | --- | --- | --- | --- | --- | --- | --- | --- | --- | --- | --- | --- | --- | --- | --- | --- | --- | --- | --- |
|  | | ZFA-unknown-89 2670 t[TGCGAAGCA](http://bindr.gdcb.iastate.edu:8080/ZiFDB/controller/searchArray?site=GCAGAATGC)a 2660  2670 aACGCTTCGTt 2660 | |  |

|  |  | | | FINGER | HELIX | TRIPLET | REFERENCE NUMBER | SOURCE | | --- | --- | --- | --- | --- | | F1 | RGQELRR | [GCA](http://bindr.gdcb.iastate.edu:8080/ZiFDB/controller/searchFinger?target=GCA) | - | CoDA | | F2 | QQTNLTR | [GAA](http://bindr.gdcb.iastate.edu:8080/ZiFDB/controller/searchFinger?target=GAA) | - | CoDA | | F3 | ANRTLVH | [TGC](http://bindr.gdcb.iastate.edu:8080/ZiFDB/controller/searchFinger?target=TGC) | - | CoDA |   [ZF DNA Sequence](javascript:CoDAPopupArrayWindow("ZFA-unknown-89","RGQELRR","QQTNLTR","ANRTLVH")) | |
| --- | --- | --- | --- | --- | --- | --- | --- | --- | --- | --- | --- | --- | --- | --- | --- | --- | --- | --- | --- | --- | --- | --- | --- | --- |
|  | | ZFA-unknown-90 2691 g[TGGGCAGGG](http://bindr.gdcb.iastate.edu:8080/ZiFDB/controller/searchArray?site=GGGGCATGG)a 2701  2691 cACCCGTCCCt 2701 | |  |

|  |  | | | FINGER | HELIX | TRIPLET | REFERENCE NUMBER | SOURCE | | --- | --- | --- | --- | --- | | F1 | RRAHLQN | [GGG](http://bindr.gdcb.iastate.edu:8080/ZiFDB/controller/searchFinger?target=GGG) | - | CoDA | | F2 | QSTTLKR | [GCA](http://bindr.gdcb.iastate.edu:8080/ZiFDB/controller/searchFinger?target=GCA) | - | CoDA | | F3 | RSDHLSL | [TGG](http://bindr.gdcb.iastate.edu:8080/ZiFDB/controller/searchFinger?target=TGG) | - | CoDA |   [ZF DNA Sequence](javascript:CoDAPopupArrayWindow("ZFA-unknown-90","RRAHLQN","QSTTLKR","RSDHLSL")) | |
| --- | --- | --- | --- | --- | --- | --- | --- | --- | --- | --- | --- | --- | --- | --- | --- | --- | --- | --- | --- | --- | --- | --- | --- | --- |
|  | | ZFA-unknown-91 2735 t[GTGGATTGT](http://bindr.gdcb.iastate.edu:8080/ZiFDB/controller/searchArray?site=TGTGATGTG)a 2725  2735 aCACCTAACAt 2725 | |  |

|  |  | | | FINGER | HELIX | TRIPLET | REFERENCE NUMBER | SOURCE | | --- | --- | --- | --- | --- | | F1 | KRQHLEY | [TGT](http://bindr.gdcb.iastate.edu:8080/ZiFDB/controller/searchFinger?target=TGT) | - | CoDA | | F2 | VRHNLTR | [GAT](http://bindr.gdcb.iastate.edu:8080/ZiFDB/controller/searchFinger?target=GAT) | - | CoDA | | F3 | RRAALGP | [GTG](http://bindr.gdcb.iastate.edu:8080/ZiFDB/controller/searchFinger?target=GTG) | - | CoDA |   [ZF DNA Sequence](javascript:CoDAPopupArrayWindow("ZFA-unknown-91","KRQHLEY","VRHNLTR","RRAALGP")) | |
| --- | --- | --- | --- | --- | --- | --- | --- | --- | --- | --- | --- | --- | --- | --- | --- | --- | --- | --- | --- | --- | --- | --- | --- | --- |
|  | | ZFA-unknown-92 2742 g[GGAGTAGTG](http://bindr.gdcb.iastate.edu:8080/ZiFDB/controller/searchArray?site=GTGGTAGGA)g 2752  2742 cCCTCATCACc 2752 | |  |

|  |  | | | FINGER | HELIX | TRIPLET | REFERENCE NUMBER | SOURCE | | --- | --- | --- | --- | --- | | F1 | RKHILIH | [GTG](http://bindr.gdcb.iastate.edu:8080/ZiFDB/controller/searchFinger?target=GTG) | - | CoDA | | F2 | QRSSLVR | [GTA](http://bindr.gdcb.iastate.edu:8080/ZiFDB/controller/searchFinger?target=GTA) | - | CoDA | | F3 | QTTHLSR | [GGA](http://bindr.gdcb.iastate.edu:8080/ZiFDB/controller/searchFinger?target=GGA) | - | CoDA |   [ZF DNA Sequence](javascript:CoDAPopupArrayWindow("ZFA-unknown-92","RKHILIH","QRSSLVR","QTTHLSR")) | |
| --- | --- | --- | --- | --- | --- | --- | --- | --- | --- | --- | --- | --- | --- | --- | --- | --- | --- | --- | --- | --- | --- | --- | --- | --- |
|  | | ZFA-unknown-93 2745 a[GTAGTGGAA](http://bindr.gdcb.iastate.edu:8080/ZiFDB/controller/searchArray?site=GAAGTGGTA)g 2755  2745 tCATCACCTTc 2755 | |  |

|  |  | | | FINGER | HELIX | TRIPLET | REFERENCE NUMBER | SOURCE | | --- | --- | --- | --- | --- | | F1 | HKPNLHR | [GAA](http://bindr.gdcb.iastate.edu:8080/ZiFDB/controller/searchFinger?target=GAA) | - | CoDA | | F2 | RREVLEN | [GTG](http://bindr.gdcb.iastate.edu:8080/ZiFDB/controller/searchFinger?target=GTG) | - | CoDA | | F3 | QSTSLQR | [GTA](http://bindr.gdcb.iastate.edu:8080/ZiFDB/controller/searchFinger?target=GTA) | - | CoDA |   [ZF DNA Sequence](javascript:CoDAPopupArrayWindow("ZFA-unknown-93","HKPNLHR","RREVLEN","QSTSLQR")) | |
| --- | --- | --- | --- | --- | --- | --- | --- | --- | --- | --- | --- | --- | --- | --- | --- | --- | --- | --- | --- | --- | --- | --- | --- | --- |
|  | | ZFA-unknown-94 2748 a[GTGGAAGCA](http://bindr.gdcb.iastate.edu:8080/ZiFDB/controller/searchArray?site=GCAGAAGTG)a 2758  2748 tCACCTTCGTt 2758 | |  |

|  |  | | | FINGER | HELIX | TRIPLET | REFERENCE NUMBER | SOURCE | | --- | --- | --- | --- | --- | | F1 | RGQELRR | [GCA](http://bindr.gdcb.iastate.edu:8080/ZiFDB/controller/searchFinger?target=GCA) | - | CoDA | | F2 | QQTNLTR | [GAA](http://bindr.gdcb.iastate.edu:8080/ZiFDB/controller/searchFinger?target=GAA) | - | CoDA | | F3 | RNVALGN | [GTG](http://bindr.gdcb.iastate.edu:8080/ZiFDB/controller/searchFinger?target=GTG) | - | CoDA |   [ZF DNA Sequence](javascript:CoDAPopupArrayWindow("ZFA-unknown-94","RGQELRR","QQTNLTR","RNVALGN")) | |
| --- | --- | --- | --- | --- | --- | --- | --- | --- | --- | --- | --- | --- | --- | --- | --- | --- | --- | --- | --- | --- | --- | --- | --- | --- |
|  | | ZFA-unknown-95 2857 a[GAAGGGGAG](http://bindr.gdcb.iastate.edu:8080/ZiFDB/controller/searchArray?site=GAGGGGGAA)g 2867  2857 tCTTCCCCTCc 2867 | |  |

|  |  | | | FINGER | HELIX | TRIPLET | REFERENCE NUMBER | SOURCE | | --- | --- | --- | --- | --- | | F1 | RNTNLTR | [GAG](http://bindr.gdcb.iastate.edu:8080/ZiFDB/controller/searchFinger?target=GAG) | - | CoDA | | F2 | RREHLVR | [GGG](http://bindr.gdcb.iastate.edu:8080/ZiFDB/controller/searchFinger?target=GGG) | - | CoDA | | F3 | QDGNLGR | [GAA](http://bindr.gdcb.iastate.edu:8080/ZiFDB/controller/searchFinger?target=GAA) | - | CoDA |   [ZF DNA Sequence](javascript:CoDAPopupArrayWindow("ZFA-unknown-95","RNTNLTR","RREHLVR","QDGNLGR")) | |
| --- | --- | --- | --- | --- | --- | --- | --- | --- | --- | --- | --- | --- | --- | --- | --- | --- | --- | --- | --- | --- | --- | --- | --- | --- |
|  | | ZFA-unknown-96 2860 a[GGGGAGGAA](http://bindr.gdcb.iastate.edu:8080/ZiFDB/controller/searchArray?site=GAAGAGGGG)t 2870  2860 tCCCCTCCTTa 2870 | |  |

|  |  | | | FINGER | HELIX | TRIPLET | REFERENCE NUMBER | SOURCE | | --- | --- | --- | --- | --- | | F1 | QASNLLR | [GAA](http://bindr.gdcb.iastate.edu:8080/ZiFDB/controller/searchFinger?target=GAA) | - | CoDA | | F2 | RQDNLGR | [GAG](http://bindr.gdcb.iastate.edu:8080/ZiFDB/controller/searchFinger?target=GAG) | - | CoDA | | F3 | RIDKLGG | [GGG](http://bindr.gdcb.iastate.edu:8080/ZiFDB/controller/searchFinger?target=GGG) | - | CoDA |   [ZF DNA Sequence](javascript:CoDAPopupArrayWindow("ZFA-unknown-96","QASNLLR","RQDNLGR","RIDKLGG")) | |
| --- | --- | --- | --- | --- | --- | --- | --- | --- | --- | --- | --- | --- | --- | --- | --- | --- | --- | --- | --- | --- | --- | --- | --- | --- |
|  | | ZFA-unknown-97 2863 g[GAGGAATAG](http://bindr.gdcb.iastate.edu:8080/ZiFDB/controller/searchArray?site=TAGGAAGAG)g 2873  2863 cCTCCTTATCc 2873 | |  |

|  |  | | | FINGER | HELIX | TRIPLET | REFERENCE NUMBER | SOURCE | | --- | --- | --- | --- | --- | | F1 | RRRNLQI | [TAG](http://bindr.gdcb.iastate.edu:8080/ZiFDB/controller/searchFinger?target=TAG) | - | CoDA | | F2 | QQTNLTR | [GAA](http://bindr.gdcb.iastate.edu:8080/ZiFDB/controller/searchFinger?target=GAA) | - | CoDA | | F3 | RRDNLNR | [GAG](http://bindr.gdcb.iastate.edu:8080/ZiFDB/controller/searchFinger?target=GAG) | - | CoDA |   [ZF DNA Sequence](javascript:CoDAPopupArrayWindow("ZFA-unknown-97","RRRNLQI","QQTNLTR","RRDNLNR")) | |
| --- | --- | --- | --- | --- | --- | --- | --- | --- | --- | --- | --- | --- | --- | --- | --- | --- | --- | --- | --- | --- | --- | --- | --- | --- |
|  | | ZFA-unknown-98 2889 t[GCTGGAGTC](http://bindr.gdcb.iastate.edu:8080/ZiFDB/controller/searchArray?site=GTCGGAGCT)a 2879  2889 aCGACCTCAGt 2879 | |  |

|  |  | | | FINGER | HELIX | TRIPLET | REFERENCE NUMBER | SOURCE | | --- | --- | --- | --- | --- | | F1 | TSTLLKR | [GTC](http://bindr.gdcb.iastate.edu:8080/ZiFDB/controller/searchFinger?target=GTC) | - | CoDA | | F2 | QSAHLKR | [GGA](http://bindr.gdcb.iastate.edu:8080/ZiFDB/controller/searchFinger?target=GGA) | - | CoDA | | F3 | LKHDLRR | [GCT](http://bindr.gdcb.iastate.edu:8080/ZiFDB/controller/searchFinger?target=GCT) | - | CoDA |   [ZF DNA Sequence](javascript:CoDAPopupArrayWindow("ZFA-unknown-98","TSTLLKR","QSAHLKR","LKHDLRR")) | |
| --- | --- | --- | --- | --- | --- | --- | --- | --- | --- | --- | --- | --- | --- | --- | --- | --- | --- | --- | --- | --- | --- | --- | --- | --- |
|  | | ZFA-unknown-99 2892 t[TCTGCTGGA](http://bindr.gdcb.iastate.edu:8080/ZiFDB/controller/searchArray?site=GGAGCTTCT)g 2882  2892 aAGACGACCTc 2882 | |  |

|  |  | | | FINGER | HELIX | TRIPLET | REFERENCE NUMBER | SOURCE | | --- | --- | --- | --- | --- | | F1 | RPAKLVL | [GGA](http://bindr.gdcb.iastate.edu:8080/ZiFDB/controller/searchFinger?target=GGA) | - | CoDA | | F2 | QRSDLTR | [GCT](http://bindr.gdcb.iastate.edu:8080/ZiFDB/controller/searchFinger?target=GCT) | - | CoDA | | F3 | QRNTLKG | [TCT](http://bindr.gdcb.iastate.edu:8080/ZiFDB/controller/searchFinger?target=TCT) | - | CoDA |   [ZF DNA Sequence](javascript:CoDAPopupArrayWindow("ZFA-unknown-99","RPAKLVL","QRSDLTR","QRNTLKG")) | |
| --- | --- | --- | --- | --- | --- | --- | --- | --- | --- | --- | --- | --- | --- | --- | --- | --- | --- | --- | --- | --- | --- | --- | --- | --- |
|  | | ZFA-unknown-100 2998 c[TGTGGAAGG](http://bindr.gdcb.iastate.edu:8080/ZiFDB/controller/searchArray?site=AGGGGATGT)g 3008  2998 gACACCTTCCc 3008 | |  |

|  |  | | | FINGER | HELIX | TRIPLET | REFERENCE NUMBER | SOURCE | | --- | --- | --- | --- | --- | | F1 | RPHHLDA | [AGG](http://bindr.gdcb.iastate.edu:8080/ZiFDB/controller/searchFinger?target=AGG) | - | CoDA | | F2 | QSAHLKR | [GGA](http://bindr.gdcb.iastate.edu:8080/ZiFDB/controller/searchFinger?target=GGA) | - | CoDA | | F3 | QPHGLAH | [TGT](http://bindr.gdcb.iastate.edu:8080/ZiFDB/controller/searchFinger?target=TGT) | - | CoDA |   [ZF DNA Sequence](javascript:CoDAPopupArrayWindow("ZFA-unknown-100","RPHHLDA","QSAHLKR","QPHGLAH")) | |
| --- | --- | --- | --- | --- | --- | --- | --- | --- | --- | --- | --- | --- | --- | --- | --- | --- | --- | --- | --- | --- | --- | --- | --- | --- |
|  | | ZFA-unknown-101 2999 t[GTGGAAGGG](http://bindr.gdcb.iastate.edu:8080/ZiFDB/controller/searchArray?site=GGGGAAGTG)a 3009  2999 aCACCTTCCCt 3009 | |  |

|  |  | | | FINGER | HELIX | TRIPLET | REFERENCE NUMBER | SOURCE | | --- | --- | --- | --- | --- | | F1 | KRERLDR | [GGG](http://bindr.gdcb.iastate.edu:8080/ZiFDB/controller/searchFinger?target=GGG) | - | CoDA | | F2 | QQTNLTR | [GAA](http://bindr.gdcb.iastate.edu:8080/ZiFDB/controller/searchFinger?target=GAA) | - | CoDA | | F3 | RNVALGN | [GTG](http://bindr.gdcb.iastate.edu:8080/ZiFDB/controller/searchFinger?target=GTG) | - | CoDA |   [ZF DNA Sequence](javascript:CoDAPopupArrayWindow("ZFA-unknown-101","KRERLDR","QQTNLTR","RNVALGN")) | |
| --- | --- | --- | --- | --- | --- | --- | --- | --- | --- | --- | --- | --- | --- | --- | --- | --- | --- | --- | --- | --- | --- | --- | --- | --- |
|  | | ZFA-unknown-102 3023 t[GTGGAAAGG](http://bindr.gdcb.iastate.edu:8080/ZiFDB/controller/searchArray?site=AGGGAAGTG)g 3033  3023 aCACCTTTCCc 3033 | |  |

|  |  | | | FINGER | HELIX | TRIPLET | REFERENCE NUMBER | SOURCE | | --- | --- | --- | --- | --- | | F1 | RMAHLHA | [AGG](http://bindr.gdcb.iastate.edu:8080/ZiFDB/controller/searchFinger?target=AGG) | - | CoDA | | F2 | QQTNLTR | [GAA](http://bindr.gdcb.iastate.edu:8080/ZiFDB/controller/searchFinger?target=GAA) | - | CoDA | | F3 | RNVALGN | [GTG](http://bindr.gdcb.iastate.edu:8080/ZiFDB/controller/searchFinger?target=GTG) | - | CoDA |   [ZF DNA Sequence](javascript:CoDAPopupArrayWindow("ZFA-unknown-102","RMAHLHA","QQTNLTR","RNVALGN")) | |
| --- | --- | --- | --- | --- | --- | --- | --- | --- | --- | --- | --- | --- | --- | --- | --- | --- | --- | --- | --- | --- | --- | --- | --- | --- |
|  | | ZFA-unknown-103 3030 a[GGGGAAGGA](http://bindr.gdcb.iastate.edu:8080/ZiFDB/controller/searchArray?site=GGAGAAGGG)g 3040  3030 tCCCCTTCCTc 3040 | |  |

|  |  | | | FINGER | HELIX | TRIPLET | REFERENCE NUMBER | SOURCE | | --- | --- | --- | --- | --- | | F1 | RMERLDR | [GGA](http://bindr.gdcb.iastate.edu:8080/ZiFDB/controller/searchFinger?target=GGA) | - | CoDA | | F2 | QQTNLTR | [GAA](http://bindr.gdcb.iastate.edu:8080/ZiFDB/controller/searchFinger?target=GAA) | - | CoDA | | F3 | RIDKLGG | [GGG](http://bindr.gdcb.iastate.edu:8080/ZiFDB/controller/searchFinger?target=GGG) | - | CoDA |   [ZF DNA Sequence](javascript:CoDAPopupArrayWindow("ZFA-unknown-103","RMERLDR","QQTNLTR","RIDKLGG")) | |
| --- | --- | --- | --- | --- | --- | --- | --- | --- | --- | --- | --- | --- | --- | --- | --- | --- | --- | --- | --- | --- | --- | --- | --- | --- |
|  | | ZFA-unknown-104 3033 g[GAAGGAGCA](http://bindr.gdcb.iastate.edu:8080/ZiFDB/controller/searchArray?site=GCAGGAGAA)g 3043  3033 cCTTCCTCGTc 3043 | |  |

|  |  | | | FINGER | HELIX | TRIPLET | REFERENCE NUMBER | SOURCE | | --- | --- | --- | --- | --- | | F1 | KNTRLSV | [GCA](http://bindr.gdcb.iastate.edu:8080/ZiFDB/controller/searchFinger?target=GCA) | - | CoDA | | F2 | QSAHLKR | [GGA](http://bindr.gdcb.iastate.edu:8080/ZiFDB/controller/searchFinger?target=GGA) | - | CoDA | | F3 | LGENLRR | [GAA](http://bindr.gdcb.iastate.edu:8080/ZiFDB/controller/searchFinger?target=GAA) | - | CoDA |   [ZF DNA Sequence](javascript:CoDAPopupArrayWindow("ZFA-unknown-104","KNTRLSV","QSAHLKR","LGENLRR")) | |
| --- | --- | --- | --- | --- | --- | --- | --- | --- | --- | --- | --- | --- | --- | --- | --- | --- | --- | --- | --- | --- | --- | --- | --- | --- |
|  | | ZFA-unknown-105 3036 a[GGAGCAGTC](http://bindr.gdcb.iastate.edu:8080/ZiFDB/controller/searchArray?site=GTCGCAGGA)a 3046  3036 tCCTCGTCAGt 3046 | |  |

|  |  | | | FINGER | HELIX | TRIPLET | REFERENCE NUMBER | SOURCE | | --- | --- | --- | --- | --- | | F1 | TGAVLTR | [GTC](http://bindr.gdcb.iastate.edu:8080/ZiFDB/controller/searchFinger?target=GTC) | - | CoDA | | F2 | QSTTLKR | [GCA](http://bindr.gdcb.iastate.edu:8080/ZiFDB/controller/searchFinger?target=GCA) | - | CoDA | | F3 | QKPHLSR | [GGA](http://bindr.gdcb.iastate.edu:8080/ZiFDB/controller/searchFinger?target=GGA) | - | CoDA |   [ZF DNA Sequence](javascript:CoDAPopupArrayWindow("ZFA-unknown-105","TGAVLTR","QSTTLKR","QKPHLSR")) | |
| --- | --- | --- | --- | --- | --- | --- | --- | --- | --- | --- | --- | --- | --- | --- | --- | --- | --- | --- | --- | --- | --- | --- | --- | --- |
|  | | ZFA-unknown-106 3049 a[GATGACTGC](http://bindr.gdcb.iastate.edu:8080/ZiFDB/controller/searchArray?site=TGCGACGAT)t 3039  3049 tCTACTGACGa 3039 | |  |

|  |  | | | FINGER | HELIX | TRIPLET | REFERENCE NUMBER | SOURCE | | --- | --- | --- | --- | --- | | F1 | RSRNLDI | [TGC](http://bindr.gdcb.iastate.edu:8080/ZiFDB/controller/searchFinger?target=TGC) | - | CoDA | | F2 | DRGNLTR | [GAC](http://bindr.gdcb.iastate.edu:8080/ZiFDB/controller/searchFinger?target=GAC) | - | CoDA | | F3 | LGNNLKR | [GAT](http://bindr.gdcb.iastate.edu:8080/ZiFDB/controller/searchFinger?target=GAT) | - | CoDA |   [ZF DNA Sequence](javascript:CoDAPopupArrayWindow("ZFA-unknown-106","RSRNLDI","DRGNLTR","LGNNLKR")) | |
| --- | --- | --- | --- | --- | --- | --- | --- | --- | --- | --- | --- | --- | --- | --- | --- | --- | --- | --- | --- | --- | --- | --- | --- | --- |
|  | | ZFA-unknown-107 3052 t[TAAGATGAC](http://bindr.gdcb.iastate.edu:8080/ZiFDB/controller/searchArray?site=GACGATTAA)t 3042  3052 aATTCTACTGa 3042 | |  |

|  |  | | | FINGER | HELIX | TRIPLET | REFERENCE NUMBER | SOURCE | | --- | --- | --- | --- | --- | | F1 | EEVNLRR | [GAC](http://bindr.gdcb.iastate.edu:8080/ZiFDB/controller/searchFinger?target=GAC) | - | CoDA | | F2 | VRHNLTR | [GAT](http://bindr.gdcb.iastate.edu:8080/ZiFDB/controller/searchFinger?target=GAT) | - | CoDA | | F3 | QQGNLQL | [TAA](http://bindr.gdcb.iastate.edu:8080/ZiFDB/controller/searchFinger?target=TAA) | - | CoDA |   [ZF DNA Sequence](javascript:CoDAPopupArrayWindow("ZFA-unknown-107","EEVNLRR","VRHNLTR","QQGNLQL")) | |
| --- | --- | --- | --- | --- | --- | --- | --- | --- | --- | --- | --- | --- | --- | --- | --- | --- | --- | --- | --- | --- | --- | --- | --- | --- |
|  | | ZFA-unknown-108 3082 a[GAAGAAAGG](http://bindr.gdcb.iastate.edu:8080/ZiFDB/controller/searchArray?site=AGGGAAGAA)c 3092  3082 tCTTCTTTCCg 3092 | |  |

|  |  | | | FINGER | HELIX | TRIPLET | REFERENCE NUMBER | SOURCE | | --- | --- | --- | --- | --- | | F1 | RMAHLHA | [AGG](http://bindr.gdcb.iastate.edu:8080/ZiFDB/controller/searchFinger?target=AGG) | - | CoDA | | F2 | QQTNLTR | [GAA](http://bindr.gdcb.iastate.edu:8080/ZiFDB/controller/searchFinger?target=GAA) | - | CoDA | | F3 | QTNNLNR | [GAA](http://bindr.gdcb.iastate.edu:8080/ZiFDB/controller/searchFinger?target=GAA) | - | CoDA |   [ZF DNA Sequence](javascript:CoDAPopupArrayWindow("ZFA-unknown-108","RMAHLHA","QQTNLTR","QTNNLNR")) | |
| --- | --- | --- | --- | --- | --- | --- | --- | --- | --- | --- | --- | --- | --- | --- | --- | --- | --- | --- | --- | --- | --- | --- | --- | --- |
|  | | ZFA-unknown-109 3111 t[GGAGGAGGA](http://bindr.gdcb.iastate.edu:8080/ZiFDB/controller/searchArray?site=GGAGGAGGA)a 3121  3111 aCCTCCTCCTt 3121 | |  |

|  |  | | | FINGER | HELIX | TRIPLET | REFERENCE NUMBER | SOURCE | | --- | --- | --- | --- | --- | | F1 | RTDRLIR | [GGA](http://bindr.gdcb.iastate.edu:8080/ZiFDB/controller/searchFinger?target=GGA) | - | CoDA | | F2 | QSAHLKR | [GGA](http://bindr.gdcb.iastate.edu:8080/ZiFDB/controller/searchFinger?target=GGA) | - | CoDA | | F3 | QMSHLKR | [GGA](http://bindr.gdcb.iastate.edu:8080/ZiFDB/controller/searchFinger?target=GGA) | - | CoDA |   [ZF DNA Sequence](javascript:CoDAPopupArrayWindow("ZFA-unknown-109","RTDRLIR","QSAHLKR","QMSHLKR")) | |
| --- | --- | --- | --- | --- | --- | --- | --- | --- | --- | --- | --- | --- | --- | --- | --- | --- | --- | --- | --- | --- | --- | --- | --- | --- |
|  | | ZFA-unknown-110 3112 g[GAGGAGGAA](http://bindr.gdcb.iastate.edu:8080/ZiFDB/controller/searchArray?site=GAAGAGGAG)a 3122  3112 cCTCCTCCTTt 3122 | |  |

|  |  | | | FINGER | HELIX | TRIPLET | REFERENCE NUMBER | SOURCE | | --- | --- | --- | --- | --- | | F1 | QASNLLR | [GAA](http://bindr.gdcb.iastate.edu:8080/ZiFDB/controller/searchFinger?target=GAA) | - | CoDA | | F2 | RQDNLGR | [GAG](http://bindr.gdcb.iastate.edu:8080/ZiFDB/controller/searchFinger?target=GAG) | - | CoDA | | F3 | RVDNLPR | [GAG](http://bindr.gdcb.iastate.edu:8080/ZiFDB/controller/searchFinger?target=GAG) | - | CoDA |   [ZF DNA Sequence](javascript:CoDAPopupArrayWindow("ZFA-unknown-110","QASNLLR","RQDNLGR","RVDNLPR")) | |
| --- | --- | --- | --- | --- | --- | --- | --- | --- | --- | --- | --- | --- | --- | --- | --- | --- | --- | --- | --- | --- | --- | --- | --- | --- |
|  | | ZFA-unknown-111 3125 a[GGTGGATAG](http://bindr.gdcb.iastate.edu:8080/ZiFDB/controller/searchArray?site=TAGGGAGGT)c 3135  3125 tCCACCTATCg 3135 | |  |

|  |  | | | FINGER | HELIX | TRIPLET | REFERENCE NUMBER | SOURCE | | --- | --- | --- | --- | --- | | F1 | RGHNLLV | [TAG](http://bindr.gdcb.iastate.edu:8080/ZiFDB/controller/searchFinger?target=TAG) | - | CoDA | | F2 | QSAHLKR | [GGA](http://bindr.gdcb.iastate.edu:8080/ZiFDB/controller/searchFinger?target=GGA) | - | CoDA | | F3 | IRHHLKR | [GGT](http://bindr.gdcb.iastate.edu:8080/ZiFDB/controller/searchFinger?target=GGT) | - | CoDA |   [ZF DNA Sequence](javascript:CoDAPopupArrayWindow("ZFA-unknown-111","RGHNLLV","QSAHLKR","IRHHLKR")) | |
| --- | --- | --- | --- | --- | --- | --- | --- | --- | --- | --- | --- | --- | --- | --- | --- | --- | --- | --- | --- | --- | --- | --- | --- | --- |
|  | | ZFA-unknown-112 3146 a[TGTGGGAAC](http://bindr.gdcb.iastate.edu:8080/ZiFDB/controller/searchArray?site=AACGGGTGT)t 3136  3146 tACACCCTTGa 3136 | |  |

|  |  | | | FINGER | HELIX | TRIPLET | REFERENCE NUMBER | SOURCE | | --- | --- | --- | --- | --- | | F1 | HRTNLIA | [AAC](http://bindr.gdcb.iastate.edu:8080/ZiFDB/controller/searchFinger?target=AAC) | - | CoDA | | F2 | RREHLVR | [GGG](http://bindr.gdcb.iastate.edu:8080/ZiFDB/controller/searchFinger?target=GGG) | - | CoDA | | F3 | QRHGLSS | [TGT](http://bindr.gdcb.iastate.edu:8080/ZiFDB/controller/searchFinger?target=TGT) | - | CoDA |   [ZF DNA Sequence](javascript:CoDAPopupArrayWindow("ZFA-unknown-112","HRTNLIA","RREHLVR","QRHGLSS")) | |
| --- | --- | --- | --- | --- | --- | --- | --- | --- | --- | --- | --- | --- | --- | --- | --- | --- | --- | --- | --- | --- | --- | --- | --- | --- |
|  | | ZFA-unknown-113 3156 c[GGAGAGGCT](http://bindr.gdcb.iastate.edu:8080/ZiFDB/controller/searchArray?site=GCTGAGGGA)a 3166  3156 gCCTCTCCGAt 3166 | |  |

|  |  | | | FINGER | HELIX | TRIPLET | REFERENCE NUMBER | SOURCE | | --- | --- | --- | --- | --- | | F1 | NKQALDR | [GCT](http://bindr.gdcb.iastate.edu:8080/ZiFDB/controller/searchFinger?target=GCT) | - | CoDA | | F2 | RQDNLGR | [GAG](http://bindr.gdcb.iastate.edu:8080/ZiFDB/controller/searchFinger?target=GAG) | - | CoDA | | F3 | QANHLSR | [GGA](http://bindr.gdcb.iastate.edu:8080/ZiFDB/controller/searchFinger?target=GGA) | - | CoDA |   [ZF DNA Sequence](javascript:CoDAPopupArrayWindow("ZFA-unknown-113","NKQALDR","RQDNLGR","QANHLSR")) | |
| --- | --- | --- | --- | --- | --- | --- | --- | --- | --- | --- | --- | --- | --- | --- | --- | --- | --- | --- | --- | --- | --- | --- | --- | --- |
|  | | ZFA-unknown-114 3171 g[GTGGCATAG](http://bindr.gdcb.iastate.edu:8080/ZiFDB/controller/searchArray?site=TAGGCAGTG)n 3181  3171 cCACCGTATCn 3181 | |  |
