# Supplementary material for: Proviral HIV-genome-wide and pol-gene specific Zinc Finger Nucleases: Usability for targeted HIV gene therapy
Source: Theor Biol Med Model. 2011 Jul 22;8:26. doi: 10.1186/1742-4682-8-26 (PMC3152896; doi:10.1186/1742-4682-8-26)
Supplement: Additional file 2 — Two ZFNs cleaving within the SIV/HIV-pol gene. This file offers a list of the two zinc finger nucleases that specifically target and cleave within DNA sequences of the SIV/HIV-pol gene, detailing their alpha-helical recognition sequences and target DNA sites. [file 1742-4682-8-26-S2.DOC]

**Zinc Finger Site Type:** Nuclease
**Zinc Finger Engineering Method:** CoDA
**Sequence Name** : SIV/HIV-pol
**Sequence Length**:3182
**Nucleotide Sequence** :nTTGGAATTGTGGGAAAGAGGGACACTCTGCAAGGCAATGCAGAGCCCCAAGAAGACAGGGATGCTGGAAATGTGGAAAAATGGACCATGTTATGGCCAAATGCCCAGACAGACAGGCGGGTTTTTTAGGCCTTGGTCCATGGGGAAAGAAGCCCCGCAATTTCCCCATGGCTCAAGTGCATCAGGGGCTGATGCCAACTGCTCCCCCAGAGGACCCAGCTGTGGATCTGCTAAAGAACTACATGCAGTTGGGCAAGCAGCAGAGAGAAAAGCAGAGAGAAAGCAGAGAGAAGCCTTACAAGGAGGTGACAGAGGATTTGCTGCACCTCAATTCTCTCTTTGGAGGAGACCAGTAGTCACTGCTCATATTGAAGGACAGCCTGTAGAAGTATTACTGGATACAGGGGCTGATGATTCTATTGTAACAGGAATAGAGTTAGGTCCACATTATACCCCAAAAATAGTAGGAGGAATAGGAGGTTTTATTAATACTAAAGAATACAAAAATGTAGAAATAGAAGTTTTAGGCAAAAGGATTAAAGGGACAATCATGACAGGGGACACCCCGATTAACATTTTTGGTAGAAATTTGCTAACAGCTCTGGGGATGTCTCTAAATTTTCCCATAGCTAAAGTAGAGCCTGTAAAAGTCGCCTTAAAGCCAGGAAAGGATGGACCAAAATTGAAGCAGTGGCCATTATCAAAAGAAAAGATAGTTGCATTAAGAGAAATCTGTGAAAAGATGGAAAAGGATGGTCAGTTGGAGGAAGCTCCCCCGACCAATCCATACAACACCCCCACATTTGCTATAAAGAAAAAGGATAAGAACAAATGGAGAATGCTGATAGATTTTAGGGAACTAAATAGGGTCACTCAGGACTTTACGGAAGTCCAATTAGGAATACCACACCCTGCAGGACTAGCAAAAAGGAAAAGAATTACAGTACTGGATATAGGTGATGCATATTTCTCCATACCTCTAGATGAAGAATTTAGGCAGTACACTGCCTTTACTTTACCATCAGTAAATAATGCAGAGCCAGGAAAACGATACATTTATAAGGTTCTGCCTCAGGGATGGAAGGGGTCACCAGCCATCTTCCAATACACTATGAGACATGTGCTAGAACCCTTCAGGAAGGCAAATCCAGATGTGACCTTAGTCCAGTATATGGATGACATCTTAATAGCTAGTGACAGGACAGACCTGGAACATGACAGGGTAGTTTTACAGTCAAAGGAACTCTTGAATAGCATAGGGTTTTCTACCCCAGAAGAGAAATTCCAAAAAGATCCCCCATTTCAATGGATGGGGTACGAATTGTGGCCAACAAAATGGAAGTTGCAAAAGATAGAGTTGCCACAAAGAGAGACCTGGACAGTGAATGATATACAGAAGTTAGTAGGAGTATTAAATTGGGCAGCTCAAATTTATCCAGGTATAAAAACCAAACATCTCTGTAGGTTAATTAGAGGAAAAATGACTCTAACAGAGGAAGTTCAGTGGACTGAGATGGCAGAAGCAGAATATGAGGAAAATAAAATAATTCTCAGTCAGGAACAAGAAGGATGTTATTACCAAGAAGGCAAGCCATTAGAAGCCACGGTAATAAAGAGTCAGGACAATCAGTGGTCTTATAAAATTCACCAAGAAGACAAAATACTGAAAGTAGGAAAATTTGCAAAGATAAAGAATACACATACCAATGGAGTGAGACTATTAGCACATGTAATACAGAAAATAGGAAAGGAAGCAATAGTGATCTGGGGACAGGTCCCAAAATTCCACTTACCAGTTGAGAAGGATGTATGGGAACAGTGGTGGACAGACTATTGGCAGGTAACCTGGATACCGGAATGGGATTTTATCTCAACACCACCGCTAGTAAGATTAGTCTTCAATCTAGTGAAGGACCCTATAGAGGGAGAAGAAACCTATTATACAGATGGATCATGTAATAAACAGTCAAAAGAAGGGAAAGCAGGATATATCACAGATAGGGGCAAAGACAAAGTAAAAGTGTTAGAACAGACTACTAATCAACAAGCAGAATTGGAAGCATTTCTCATGGCATTGACAGACTCAGGGCCAAAGGCAAATATTATAGTAGATTCACAATATGTTATGGGAATAATAACAGGATGCCCTACAGAATCAGAGAGCAGGCTAGTTAATCAAATAATAGAAGAAATGATTAAAAAGTCAGAAATTTATGTAGCATGGGTACCAGCACACAAAGGTATAGGAGGAAACCAAGAAATAGACCACCTAGTTAGTCAAGGGATTAGACAAGTTCTCTTCTTGGAAAAGATAGAGCCAGCACAAGAAGAACATGATAAATACCATAGTAATGTAAAAGAATTGGTATTCAAATTTGGATTACCCAGAATAGTGGCCAGACAGATAGTAGACACCTGTGATAAATGTCATCAGAAAGGAGAGGCTATACATGGGCAGGCAAATTCAGATCTAGGGACTTGGCAAATGGATTGTACCCATCTAGAGGGAAAAATAATCATAGTTGCAGTACATGTAGCTAGTGGATTCATAGAAGCAGAGGTAATTCCACAAGAGACAGGAAGACAGACAGCACTATTTCTGTTAAAATTGGCAGGCAGATGGCCTATTACACATCTACACACAGATAATGGTGCTAACTTTGCTTCGCAAGAAGTAAAGATGGTTGCATGGTGGGCAGGGATAGAGCACACCTTTGGGGTACCATACAATCCACAGAGTCAGGGAGTAGTGGAAGCAATGAATCACCACCTGAAAAATCAAATAGATAGAATCAGGGAACAAGCAAATTCAGTAGAAACCATAGTATTAATGGCAGTTCATTGCATGAATTTTAAAAGAAGGGGAGGAATAGGGGATATGACTCCAGCAGAAAGATTAATTAACATGATCACTACAGAACAAGAGATACAATTTCAACAATCAAAAAACTCAAAATTTAAAAATTTTCGGGTCTATTACAGAGAAGGCAGAGATCAACTGTGGAAGGGACCCGGTGAGCTATTGTGGAAAGGGGAAGGAGCAGTCATCTTAAAGGTAGGGACAGACATTAAGGTAGTACCCAGAAGAAAGGCTAAAATTATCAAAGATTATGGAGGAGGAAAAGAGGTGGATAGCAGTTCCCACATGGAGGATACCGGAGAGGCTAGAGAGGTGGCATAGn
**Selected Module Sets:**
**Left Module Count:** 3
**Spacer Nucleotide Count:** 5,6,7
**Right Module Count:** 3
**Ignore Asp Overlap:** False

The results below are zinc finger Nucleases that can be constructed using CoDA. Note that other methods (including modular assembly and OPEN) can also potentially be used to target the input sequence of interest.”

**Sort By: Hide intron splice sites**

**[

](http://zifit.partners.org/ZiFiT/CoDAZiFiTNuclease.aspx#ctl00_ContentPlaceHolder1_tree12_SkipLink)**

| ZFN-unknown-SP-7-1 1063 gTTCTGCCTCAGGGATG[GAAGGGGTC](http://bindr.gdcb.iastate.edu:8080/ZiFDB/controller/searchArray?site=GTCGGGGAA)a 1089  1063 c[AAGACGGAG](http://bindr.gdcb.iastate.edu:8080/ZiFDB/controller/searchArray?site=GAAGCAGAG)TCCCTACCTTCCCCAGt 1089 |
| --- |

|  | 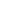 | | | FINGER | HELIX | TRIPLET | REFERENCE NUMBER | SOURCE | | --- | --- | --- | --- | --- | | Left F1 | QGSNLAR | [GAA](http://bindr.gdcb.iastate.edu:8080/ZiFDB/controller/searchFinger?target=GAA) | - | CoDA | | Left F2 | QSTTLKR | [GCA](http://bindr.gdcb.iastate.edu:8080/ZiFDB/controller/searchFinger?target=GCA) | - | CoDA | | Left F3 | RGDNLNR | [GAG](http://bindr.gdcb.iastate.edu:8080/ZiFDB/controller/searchFinger?target=GAG) | - | CoDA | | Right F1 | TKSLLAR | [GTC](http://bindr.gdcb.iastate.edu:8080/ZiFDB/controller/searchFinger?target=GTC) | - | CoDA | | Right F2 | RREHLVR | [GGG](http://bindr.gdcb.iastate.edu:8080/ZiFDB/controller/searchFinger?target=GGG) | - | CoDA | | Right F3 | QDGNLGR | [GAA](http://bindr.gdcb.iastate.edu:8080/ZiFDB/controller/searchFinger?target=GAA) | - | CoDA |   [ZF DNA Sequence](javascript:CoDAPopupNucleaseWindow("Left-ZFN-unknown-SP-7-1","QGSNLAR","QSTTLKR","RGDNLNR","Right-ZFN-unknown-SP-7-1","TKSLLAR","RREHLVR","QDGNLGR")) | |
| --- | --- | --- | --- | --- | --- | --- | --- | --- | --- | --- | --- | --- | --- | --- | --- | --- | --- | --- | --- | --- | --- | --- | --- | --- | --- | --- | --- | --- | --- | --- | --- | --- | --- | --- | --- | --- | --- | --- | --- |
| [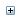](javascript:TreeView_ToggleNode(ctl00_ContentPlaceHolder1_tree12_Data,2,document.getElementById('ctl00_ContentPlaceHolder1_tree12n2'),'%20',document.getElementById('ctl00_ContentPlaceHolder1_tree12n2Nodes'))) | | ZFN-unknown-SP-5-1 1871 cAACACCACCGCTAG[TAAGATTAG](http://bindr.gdcb.iastate.edu:8080/ZiFDB/controller/searchArray?site=TAGGATTAA)t 1895  1871 g[TTGTGGTGG](http://bindr.gdcb.iastate.edu:8080/ZiFDB/controller/searchArray?site=GTTGGTGGT)CGATCATTCTAATCa 1895 | |  |

| FINGER | HELIX | TRIPLET | REFERENCE NUMBER | SOURCE |
| --- | --- | --- | --- | --- |
| Left F1 | AATALRR | [GTT](http://bindr.gdcb.iastate.edu:8080/ZiFDB/controller/searchFinger?target=GTT) | - | CoDA |
| Left F2 | EAHHLSR | [GGT](http://bindr.gdcb.iastate.edu:8080/ZiFDB/controller/searchFinger?target=GGT) | - | CoDA |
| Left F3 | IRHHLKR | [GGT](http://bindr.gdcb.iastate.edu:8080/ZiFDB/controller/searchFinger?target=GGT) | - | CoDA |
| Right F1 | RSHNLRL | [TAG](http://bindr.gdcb.iastate.edu:8080/ZiFDB/controller/searchFinger?target=TAG) | - | CoDA |
| Right F2 | VRHNLTR | [GAT](http://bindr.gdcb.iastate.edu:8080/ZiFDB/controller/searchFinger?target=GAT) | - | CoDA |
| Right F3 | QQGNLQL | [TAA](http://bindr.gdcb.iastate.edu:8080/ZiFDB/controller/searchFinger?target=TAA) | - | CoDA |

[ZF DNA Sequence](javascript:CoDAPopupNucleaseWindow("Left-ZFN-unknown-SP-5-1","AATALRR","EAHHLSR","IRHHLKR","Right-ZFN-unknown-SP-5-1","RSHNLRL","VRHNLTR","QQGNLQL"))

Blast AACACCACCNNNNNTAAGATTAG
