# Supplementary material for: Proviral HIV-genome-wide and pol-gene specific Zinc Finger Nucleases: Usability for targeted HIV gene therapy
Source: Theor Biol Med Model. 2011 Jul 22;8:26. doi: 10.1186/1742-4682-8-26 (PMC3152896; doi:10.1186/1742-4682-8-26)
Supplement: Additional file 3 — A detailed list of the 15 ZFN cleaving within the HIV-1 whole genome. This file offers a list of the 15 zinc finger nucleases that specifically target and cleave within >18 DNA- bp-sequences of the HIV-1 whole genome; detailing their alpha helical recognition sequences, and target-DNA sites. [file 1742-4682-8-26-S3.DOC]

**Zinc Finger Site Type:** Nuclease
**Zinc Finger Engineering Method:** CoDA
**Sequence Name** : HIV-1 Complete genome (dsDNA)
**Sequence Length**:9183
**Nucleotide Sequence** :nGGTCTCTCTGGTTAGACCAGATCTGAGCCTGGGAGCTCTCTGGCTAACTAGGGAACCCACTGCTTAAGCCTCAATAAAGCTTGCCTTGAGTGCTTCAAGTAGTGTGTGCCCGTCTGTTGTGTGACTCTGGTAACTAGAGATCCCTCAGACCCTTTTAGTCAGTGTGGAAAATCTCTAGCAGTGGCGCCCGAACAGGGACCTGAAAGCGAAAGGGAAACCAGAGGAGCTCTCTCGACGCAGGACTCGGCTTGCTGAAGCGCGCACGGCAAGAGGCGAGGGGCGGCGACTGGTGAGTACGCCAAAAATTTTGACTAGCGGAGGCTAGAAGGAGAGAGATGGGTGCGAGAGCGTCAGTATTAAGCGGGGGAGAATTAGATCGATGGGAAAAAATTCGGTTAAGGCCAGGGGGAAAGAAAAAATATAAATTAAAACATATAGTATGGGCAAGCAGGGAGCTAGAACGATTCGCAGTTAATCCTGGCCTGTTAGAAACATCAGAAGGCTGTAGACAAATACTGGGACAGCTACAACCATCCCTTCAGACAGGATCAGAAGAACTTAGATCATTATATAATACAGTAGCAACCCTCTATTGTGTGCATCAAAGGATAGAGATAAAAGACACCAAGGAAGCTTTAGACAAGATAGAGGAAGAGCAAAACAAAAGTAAGAAAAAAGCACAGCAAGCAGCAGCTGACACAGGACACAGCAATCAGGTCAGCCAAAATTACCCTATAGTGCAGAACATCCAGGGGCAAATGGTACATCAGGCCATATCACCTAGAACTTTAAATGCATGGGTAAAAGTAGTAGAAGAGAAGGCTTTCAGCCCAGAAGTGATACCCATGTTTTCAGCATTATCAGAAGGAGCCACCCCACAAGATTTAAACACCATGCTAAACACAGTGGGGGGACATCAAGCAGCCATGCAAATGTTAAAAGAGACCATCAATGAGGAAGCTGCAGAATGGGATAGAGTGCATCCAGTGCATGCAGGGCCTATTGCACCAGGCCAGATGAGAGAACCAAGGGGAAGTGACATAGCAGGAACTACTAGTACCCTTCAGGAACAAATAGGATGGATGACAAATAATCCACCTATCCCAGTAGGAGAAATTTATAAAAGATGGATAATCCTGGGATTAAATAAAATAGTAAGAATGTATAGCCCTACCAGCATTCTGGACATAAGACAAGGACCAAAGGAACCCTTTAGAGACTATGTAGACCGGTTCTATAAAACTCTAAGAGCCGAGCAAGCTTCACAGGAGGTAAAAAATTGGATGACAGAAACCTTGTTGGTCCAAAATGCGAACCCAGATTGTAAGACTATTTTAAAAGCATTGGGACCAGCGGCTACACTAGAAGAAATGATGACAGCATGTCAGGGAGTAGGAGGACCCGGCCATAAGGCAAGAGTTTTGGCTGAAGCAATGAGCCAAGTAACAAATTCAGCTACCATAATGATGCAGAGAGGCAATTTTAGGAACCAAAGAAAGATTGTTAAGTGTTTCAATTGTGGCAAAGAAGGGCACACAGCCAGAAATTGCAGGGCCCCTAGGAAAAAGGGCTGTTGGAAATGTGGAAAGGAAGGACACCAAATGAAAGATTGTACTGAGAGACAGGCTAATTTTTTAGGGAAGATCTGGCCTTCCTACAAGGGAAGGCCAGGGAATTTTCTTCAGAGCAGACCAGAGCCAACAGCCCCACCAGAAGAGAGCTTCAGGTCTGGGGTAGAGACAACAACTCCCCCTCAGAAGCAGGAGCCGATAGACAAGGAACTGTATCCTTTAACTTCCCTCAGGTCACTCTTTGGCAACGACCCCTCGTCACAATAAAGATAGGGGGGCAACTAAAGGAAGCTCTATTAGATACAGGAGCAGATGATACAGTATTAGAAGAAATGAGTTTGCCAGGAAGATGGAAACCAAAAATGATAGGGGGAATTGGAGGTTTTATCAAAGTAAGACAGTATGATCAGATACTCATAGAAATCTGTGGACATAAAGCTATAGGTACAGTATTAGTAGGACCTACACCTGTCAACATAATTGGAAGAAATCTGTTGACTCAGATTGGTTGCACTTTAAATTTTCCCATTAGCCCTATTGAGACTGTACCAGTAAAATTAAAGCCAGGAATGGATGGCCCAAAAGTTAAACAATGGCCATTGACAGAAGAAAAAATAAAAGCATTAGTAGAAATTTGTACAGAGATGGAAAAGGAAGGGAAAATTTCAAAAATTGGGCCTGAAAATCCATACAATACTCCAGTATTTGCCATAAAGAAAAAAGACAGTACTAAATGGAGAAAATTAGTAGATTTCAGAGAACTTAATAAGAGAACTCAAGACTTCTGGGAAGTTCAATTAGGAATACCACATCCCGCAGGGTTAAAAAAGAAAAAATCAGTAACAGTACTGGATGTGGGTGATGCATATTTTTCAGTTCCCTTAGATGAAGACTTCAGGAAGTATACTGCATTTACCATACCTAGTATAAACAATGAGACACCAGGGATTAGATATCAGTACAATGTGCTTCCACAGGGATGGAAAGGATCACCAGCAATATTCCAAAGTAGCATGACAAAAATCTTAGAGCCTTTTAGAAAACAAAATCCAGACATAGTTATCTATCAATACATGGATGATTTGTATGTAGGATCTGACTTAGAAATAGGGCAGCATAGAACAAAAATAGAGGAGCTGAGACAACATCTGTTGAGGTGGGGACTTACCACACCAGACAAAAAACATCAGAAAGAACCTCCATTCCTTTGGATGGGTTATGAACTCCATCCTGATAAATGGACAGTACAGCCTATAGTGCTGCCAGAAAAAGACAGCTGGACTGTCAATGACATACAGAAGTTAGTGGGGAAATTGAATTGGGCAAGTCAGATTTACCCAGGGATTAAAGTAAGGCAATTATGTAAACTCCTTAGAGGAACCAAAGCACTAACAGAAGTAATACCACTAACAGAAGAAGCAGAGCTAGAACTGGCAGAAAACAGAGAGATTCTAAAAGAACCAGTACATGGAGTGTATTATGACCCATCAAAAGACTTAATAGCAGAAATACAGAAGCAGGGGCAAGGCCAATGGACATATCAAATTTATCAAGAGCCATTTAAAAATCTGAAAACAGGAAAATATGCAAGAATGAGGGGTGCCCACACTAATGATGTAAAACAATTAACAGAGGCAGTGCAAAAAATAACCACAGAAAGCATAGTAATATGGGGAAAGACTCCTAAATTTAAACTGCCCATACAAAAGGAAACATGGGAAACATGGTGGACAGAGTATTGGCAAGCCACCTGGATTCCTGAGTGGGAGTTTGTTAATACCCCTCCCTTAGTGAAATTATGGTACCAGTTAGAGAAAGAACCCATAGTAGGAGCAGAAACCTTCTATGTAGATGGGGCAGCTAACAGGGAGACTAAATTAGGAAAAGCAGGATATGTTACTAATAGAGGAAGACAAAAAGTTGTCACCCTAACTGACACAACAAATCAGAAGACTGAGTTACAAGCAATTTATCTAGCTTTGCAGGATTCGGGATTAGAAGTAAACATAGTAACAGACTCACAATATGCATTAGGAATCATTCAAGCACAACCAGATCAAAGTGAATCAGAGTTAGTCAATCAAATAATAGAGCAGTTAATAAAAAAGGAAAAGGTCTATCTGGCATGGGTACCAGCACACAAAGGAATTGGAGGAAATGAACAAGTAGATAAATTAGTCAGTGCTGGAATCAGGAAAGTACTATTTTTAGATGGAATAGATAAGGCCCAAGATGAACATGAGAAATATCACAGTAATTGGAGAGCAATGGCTAGTGATTTTAACCTGCCACCTGTAGTAGCAAAAGAAATAGTAGCCAGCTGTGATAAATGTCAGCTAAAAGGAGAAGCCATGCATGGACAAGTAGACTGTAGTCCAGGAATATGGCAACTAGATTGTACACATTTAGAAGGAAAAGTTATCCTGGTAGCAGTTCATGTAGCCAGTGGATATATAGAAGCAGAAGTTATTCCAGCAGAAACAGGGCAGGAAACAGCATATTTTCTTTTAAAATTAGCAGGAAGATGGCCAGTAAAAACAATACATACTGACAATGGCAGCAATTTCACCGGTGCTACGGTTAGGGCCGCCTGTTGGTGGGCGGGAATCAAGCAGGAATTTGGAATTCCCTACAATCCCCAAAGTCAAGGAGTAGTAGAATCTATGAATAAAGAATTAAAGAAAATTATAGGACAGGTAAGAGATCAGGCTGAACATCTTAAGACAGCAGTACAAATGGCAGTATTCATCCACAATTTTAAAAGAAAAGGGGGGATTGGGGGGTACAGTGCAGGGGAAAGAATAGTAGACATAATAGCAACAGACATACAAACTAAAGAATTACAAAAACAAATTACAAAAATTCAAAATTTTCGGGTTTATTACAGGGACAGCAGAAATCCACTTTGGAAAGGACCAGCAAAGCTCCTCTGGAAAGGTGAAGGGGCAGTAGTAATACAAGATAATAGTGACATAAAAGTAGTGCCAAGAAGAAAAGCAAAGATCATTAGGGATTATGGAAAACAGATGGCAGGTGATGATTGTGTGGCAAGTAGACAGGATGAGGATTAGAACATGGAAAAGTTTAGTAAAACACCATATGTATGTTTCAGGGAAAGCTAGGGGATGGTTTTATAGACATCACTATGAAAGCCCTCATCCAAGAATAAGTTCAGAAGTACACATCCCACTAGGGGATGCTAGATTGGTAATAACAACATATTGGGGTCTGCATACAGGAGAAAGAGACTGGCATTTGGGTCAGGGAGTCTCCATAGAATGGAGGAAAAAGAGATATAGCACACAAGTAGACCCTGAACTAGCAGACCAACTAATTCATCTGTATTACTTTGACTGTTTTTCAGACTCTGCTATAAGAAAGGCCTTATTAGGACACATAGTTAGCCCTAGGTGTGAATATCAAGCAGGACATAACAAGGTAGGATCTCTACAATACTTGGCACTAGCAGCATTAATAACACCAAAAAAGATAAAGCCACCTTTGCCTAGTGTTACGAAACTGACAGAGGATAGATGGAACAAGCCCCAGAAGACCAAGGGCCACAGAGGGAGCCACACAATGAATGGACACTAGAGCTTTTAGAGGAGCTTAAGAATGAAGCTGTTAGACATTTTCCTAGGATTTGGCTCCATGGCTTAGGGCAACATATCTATGAAACTTATGGGGATACTTGGGCAGGAGTGGAAGCCATAATAAGAATTCTGCAACAACTGCTGTTTATCCATTTTCAGAATTGGGTGTCGACATAGCAGAATAGGCGTTACTCGACAGAGGAGAGCAAGAAATGGAGCCAGTAGATCCTAGACTAGAGCCCTGGAAGCATCCAGGAAGTCAGCCTAAAACTGCTTGTACCAATTGCTATTGTAAAAAGTGTTGCTTTCATTGCCAAGTTTGTTTCATAACAAAAGCCTTAGGCATCTCCTATGGCAGGAAGAAGCGGAGACAGCGACGAAGAGCTCATCAGAACAGTCAGACTCATCAAGCTTCTCTATCAAAGCAGTAAGTAGTACATGTAATGCAACCTATACCAATAGTAGCAATAGTAGCATTAGTAGTAGCAATAATAATAGCAATAGTTGTGTGGTCCATAGTAATCATAGAATATAGGAAAATATTAAGACAAAGAAAAATAGACAGGTTAATTGATAGACTAATAGAAAGAGCAGAAGACAGTGGCAATGAGAGTGAAGGAGAAATATCAGCACTTGTGGAGATGGGGGTGGAGATGGGGCACCATGCTCCTTGGGATGTTGATGATCTGTAGTGCTACAGAAAAATTGTGGGTCACAGTCTATTATGGGGTACCTGTGTGGAAGGAAGCAACCACCACTCTATTTTGTGCATCAGATGCTAAAGCATATGATACAGAGGTACATAATGTTTGGGCCACACATGCCTGTGTACCCACAGACCCCAACCCACAAGAAGTAGTATTGGTAAATGTGACAGAAAATTTTAACATGTGGAAAAATGACATGGTAGAACAGATGCATGAGGATATAATCAGTTTATGGGATCAAAGCCTAAAGCCATGTGTAAAATTAACCCCACTCTGTGTTAGTTTAAAGTGCACTGATTTGAAGAATGATACTAATACCAATAGTAGTAGCGGGAGAATGATAATGGAGAAAGGAGAGATAAAAAACTGCTCTTTCAATATCAGCACAAGCATAAGAGGTAAGGTGCAGAAAGAATATGCATTTTTTTATAAACTTGATATAATACCAATAGATAATGATACTACCAGCTATAAGTTGACAAGTTGTAACACCTCAGTCATTACACAGGCCTGTCCAAAGGTATCCTTTGAGCCAATTCCCATACATTATTGTGCCCCGGCTGGTTTTGCGATTCTAAAATGTAATAATAAGACGTTCAATGGAACAGGACCATGTACAAATGTCAGCACAGTACAATGTACACATGGAATTAGGCCAGTAGTATCAACTCAACTGCTGTTAAATGGCAGTCTAGCAGAAGAAGAGGTAGTAATTAGATCTGTCAATTTCACGGACAATGCTAAAACCATAATAGTACAGCTGAACACATCTGTAGAAATTAATTGTACAAGACCCAACAACAATACAAGAAAAAGAATCCGTATCCAGAGAGGACCAGGGAGAGCATTTGTTACAATAGGAAAAATAGGAAATATGAGACAAGCACATTGTAACATTAGTAGAGCAAAATGGAATAACACTTTAAAACAGATAGCTAGCAAATTAAGAGAACAATTTGGAAATAATAAAACAATAATCTTTAAGCAATCCTCAGGAGGGGACCCAGAAATTGTAACGCACAGTTTTAATTGTGGAGGGGAATTTTTCTACTGTAATTCAACACAACTGTTTAATAGTACTTGGTTTAATAGTACTTGGAGTACTGAAGGGTCAAATAACACTGAAGGAAGTGACACAATCACCCTCCCATGCAGAATAAAACAAATTATAAACATGTGGCAGAAAGTAGGAAAAGCAATGTATGCCCCTCCCATCAGTGGACAAATTAGATGTTCATCAAATATTACAGGGCTGCTATTAACAAGAGATGGTGGTAATAGCAACAATGAGTCCGAGATCTTCAGACCTGGAGGAGGAGATATGAGGGACAATTGGAGAAGTGAATTATATAAATATAAAGTAGTAAAAATTGAACCATTAGGAGTAGCACCCACCAAGGCAAAGAGAAGAGTGGTGCAGAGAGAAAAAAGAGCAGTGGGAATAGGAGCTTTGTTCCTTGGGTTCTTGGGAGCAGCAGGAAGCACTATGGGCGCAGCCTCAATGACGCTGACGGTACAGGCCAGACAATTATTGTCTGGTATAGTGCAGCAGCAGAACAATTTGCTGAGGGCTATTGAGGCGCAACAGCATCTGTTGCAACTCACAGTCTGGGGCATCAAGCAGCTCCAGGCAAGAATCCTGGCTGTGGAAAGATACCTAAAGGATCAACAGCTCCTGGGGATTTGGGGTTGCTCTGGAAAACTCATTTGCACCACTGCTGTGCCTTGGAATGCTAGTTGGAGTAATAAATCTCTGGAACAGATTTGGAATCACACGACCTGGATGGAGTGGGACAGAGAAATTAACAATTACACAAGCTTAATACACTCCTTAATTGAAGAATCGCAAAACCAGCAAGAAAAGAATGAACAAGAATTATTGGAATTAGATAAATGGGCAAGTTTGTGGAATTGGTTTAACATAACAAATTGGCTGTGGTATATAAAATTATTCATAATGATAGTAGGAGGCTTGGTAGGTTTAAGAATAGTTTTTGCTGTACTTTCTATAGTGAATAGAGTTAGGCAGGGATATTCACCATTATCGTTTCAGACCCACCTCCCAACCCCGAGGGGACCCGACAGGCCCGAAGGAATAGAAGAAGAAGGTGGAGAGAGAGACAGAGACAGATCCATTCGATTAGTGAACGGATCCTTGGCACTTATCTGGGACGATCTGCGGAGCCTGTGCCTCTTCAGCTACCACCGCTTGAGAGACTTACTCTTGATTGTAACGAGGATTGTGGAACTTCTGGGACGCAGGGGGTGGGAAGCCCTCAAATATTGGTGGAATCTCCTACAGTATTGGAGTCAGGAACTAAAGAATAGTGCTGTTAGCTTGCTCAATGCCACAGCCATAGCAGTAGCTGAGGGGACAGATAGGGTTATAGAAGTAGTACAAGGAGCTTGTAGAGCTATTCGCCACATACCTAGAAGAATAAGACAGGGCTTGGAAAGGATTTTGCTATAAGATGGGTGGCAAGTGGTCAAAAAGTAGTGTGATTGGATGGCCTACTGTAAGGGAAAGAATGAGACGAGCTGAGCCAGCAGCAGATAGGGTGGGAGCAGCATCTCGAGACCTGGAAAAACATGGAGCAATCACAAGTAGCAATACAGCAGCTACCAATGCTGCTTGTGCCTGGCTAGAAGCACAAGAGGAGGAGGAGGTGGGTTTTCCAGTCACACCTCAGGTACCTTTAAGACCAATGACTTACAAGGCAGCTGTAGATCTTAGCCACTTTTTAAAAGAAAAGGGGGGACTGGAAGGGCTAATTCACTCCCAAAGAAGACAAGATATCCTTGATCTGTGGATCTACCACACACAAGGCTACTTCCCTGATTAGCAGAACTACACACCAGGGCCAGGGGTCAGATATCCACTGACCTTTGGATGGTGCTACAAGCTAGTACCAGTTGAGCCAGATAAGATAGAAGAGGCCAATAAAGGAGAGAACACCAGCTTGTTACACCCTGTGAGCCTGCATGGGATGGATGACCCGGAGAGAGAAGTGTTAGAGTGGAGGTTTGACAGCCGCCTAGCATTTCATCACGTGGCCCGAGAGCTGCATCCGGAGTACTTCAAGAACTGCTGACATCGAGCTTGCTACAAGGGACTTTCCGCTGGGGACTTTCCAGGGAGGCGTGGCCTGGGCGGGACTGGGGAGTGGCGAGCCCTCAGATCCTGCATATAAGCAGCTGCTTTTTGCCTGTACTGGGTCTCTCTGGTTAGACCAGATCTGAGCCTGGGAGCTCTCTGGCTAACTAGGGAACCCACTGCTTAAGCCTCAATAAAGCTTGCCTTGAGTGCTTCn
**Selected Module Sets:**
**Left Module Count:** 3
**Spacer Nucleotide Count:** 5,6,7
**Right Module Count:** 3
**Ignore Asp Overlap:**False

The results below are zinc finger arrays that can be constructed using CoDA. Note that other methods (including modular assembly and OPEN) can also potentially be used to target the input sequence of interest.”

**Sort By: Hide intron splice sites**

**[

](http://zifit.partners.org/ZiFiT/CoDAZiFiTNuclease.aspx#ctl00_ContentPlaceHolder1_tree12_SkipLink)**

| [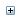](javascript:TreeView_ToggleNode(ctl00_ContentPlaceHolder1_tree12_Data,0,document.getElementById('ctl00_ContentPlaceHolder1_tree12n0'),'%20',document.getElementById('ctl00_ContentPlaceHolder1_tree12n0Nodes'))) | ZFN-unknown-SP-7-1 847 tGTTTTCAGCATTATCA[GAAGGAGCC](http://bindr.gdcb.iastate.edu:8080/ZiFDB/controller/searchArray?site=GCCGGAGAA)a 873  847 a[CAAAAGTCG](http://bindr.gdcb.iastate.edu:8080/ZiFDB/controller/searchArray?site=AACGAAGCT)TAATAGTCTTCCTCGGt 873 |
| --- | --- |

|  | 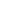 | | | FINGER | HELIX | TRIPLET | REFERENCE NUMBER | SOURCE | | --- | --- | --- | --- | --- | | Left F1 | GASALRQ | [AAC](http://bindr.gdcb.iastate.edu:8080/ZiFDB/controller/searchFinger?target=AAC) | - | CoDA | | Left F2 | QQTNLTR | [GAA](http://bindr.gdcb.iastate.edu:8080/ZiFDB/controller/searchFinger?target=GAA) | - | CoDA | | Left F3 | VGNSLTR | [GCT](http://bindr.gdcb.iastate.edu:8080/ZiFDB/controller/searchFinger?target=GCT) | - | CoDA | | Right F1 | DSPTLRR | [GCC](http://bindr.gdcb.iastate.edu:8080/ZiFDB/controller/searchFinger?target=GCC) | - | CoDA | | Right F2 | QSAHLKR | [GGA](http://bindr.gdcb.iastate.edu:8080/ZiFDB/controller/searchFinger?target=GGA) | - | CoDA | | Right F3 | LGENLRR | [GAA](http://bindr.gdcb.iastate.edu:8080/ZiFDB/controller/searchFinger?target=GAA) | - | CoDA |   [ZF DNA Sequence](javascript:CoDAPopupNucleaseWindow("Left-ZFN-unknown-SP-7-1","GASALRQ","QQTNLTR","VGNSLTR","Right-ZFN-unknown-SP-7-1","DSPTLRR","QSAHLKR","LGENLRR")) | |
| --- | --- | --- | --- | --- | --- | --- | --- | --- | --- | --- | --- | --- | --- | --- | --- | --- | --- | --- | --- | --- | --- | --- | --- | --- | --- | --- | --- | --- | --- | --- | --- | --- | --- | --- | --- | --- | --- | --- | --- |
| [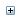](javascript:TreeView_ToggleNode(ctl00_ContentPlaceHolder1_tree12_Data,2,document.getElementById('ctl00_ContentPlaceHolder1_tree12n2'),'%20',document.getElementById('ctl00_ContentPlaceHolder1_tree12n2Nodes'))) | | ZFN-unknown-SP-6-1 1450 aTTCAGCTACCATAAT[GATGCAGAG](http://bindr.gdcb.iastate.edu:8080/ZiFDB/controller/searchArray?site=GAGGCAGAT)a 1475  1450 t[AAGTCGATG](http://bindr.gdcb.iastate.edu:8080/ZiFDB/controller/searchArray?site=GAAGCTGTA)GTATTACTACGTCTCt 1475 | |  |

|  | 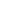 | | | FINGER | HELIX | TRIPLET | REFERENCE NUMBER | SOURCE | | --- | --- | --- | --- | --- | | Left F1 | TTTNLRR | [GAA](http://bindr.gdcb.iastate.edu:8080/ZiFDB/controller/searchFinger?target=GAA) | - | CoDA | | Left F2 | QRSDLTR | [GCT](http://bindr.gdcb.iastate.edu:8080/ZiFDB/controller/searchFinger?target=GCT) | - | CoDA | | Left F3 | QSGTLTR | [GTA](http://bindr.gdcb.iastate.edu:8080/ZiFDB/controller/searchFinger?target=GTA) | - | CoDA | | Right F1 | KHSNLAR | [GAG](http://bindr.gdcb.iastate.edu:8080/ZiFDB/controller/searchFinger?target=GAG) | - | CoDA | | Right F2 | QSTTLKR | [GCA](http://bindr.gdcb.iastate.edu:8080/ZiFDB/controller/searchFinger?target=GCA) | - | CoDA | | Right F3 | LNSNLAR | [GAT](http://bindr.gdcb.iastate.edu:8080/ZiFDB/controller/searchFinger?target=GAT) | - | CoDA |   [ZF DNA Sequence](javascript:CoDAPopupNucleaseWindow("Left-ZFN-unknown-SP-6-1","TTTNLRR","QRSDLTR","QSGTLTR","Right-ZFN-unknown-SP-6-1","KHSNLAR","QSTTLKR","LNSNLAR")) | |
| --- | --- | --- | --- | --- | --- | --- | --- | --- | --- | --- | --- | --- | --- | --- | --- | --- | --- | --- | --- | --- | --- | --- | --- | --- | --- | --- | --- | --- | --- | --- | --- | --- | --- | --- | --- | --- | --- | --- | --- |
| [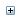](javascript:TreeView_ToggleNode(ctl00_ContentPlaceHolder1_tree12_Data,4,document.getElementById('ctl00_ContentPlaceHolder1_tree12n4'),'%20',document.getElementById('ctl00_ContentPlaceHolder1_tree12n4Nodes'))) | | ZFN-unknown-SP-6-2 1741 cAACAACTCCCCCTCA[GAAGCAGGA](http://bindr.gdcb.iastate.edu:8080/ZiFDB/controller/searchArray?site=GGAGCAGAA)g 1766  1741 g[TTGTTGAGG](http://bindr.gdcb.iastate.edu:8080/ZiFDB/controller/searchArray?site=GTTGTTGGA)GGGAGTCTTCGTCCTc 1766 | |  |

|  | 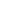 | | | FINGER | HELIX | TRIPLET | REFERENCE NUMBER | SOURCE | | --- | --- | --- | --- | --- | | Left F1 | TKPVLKI | [GTT](http://bindr.gdcb.iastate.edu:8080/ZiFDB/controller/searchFinger?target=GTT) | - | CoDA | | Left F2 | HKSSLTR | [GTT](http://bindr.gdcb.iastate.edu:8080/ZiFDB/controller/searchFinger?target=GTT) | - | CoDA | | Left F3 | QTTHLSR | [GGA](http://bindr.gdcb.iastate.edu:8080/ZiFDB/controller/searchFinger?target=GGA) | - | CoDA | | Right F1 | RTDRLIR | [GGA](http://bindr.gdcb.iastate.edu:8080/ZiFDB/controller/searchFinger?target=GGA) | - | CoDA | | Right F2 | QSTTLKR | [GCA](http://bindr.gdcb.iastate.edu:8080/ZiFDB/controller/searchFinger?target=GCA) | - | CoDA | | Right F3 | QRNNLGR | [GAA](http://bindr.gdcb.iastate.edu:8080/ZiFDB/controller/searchFinger?target=GAA) | - | CoDA |   [ZF DNA Sequence](javascript:CoDAPopupNucleaseWindow("Left-ZFN-unknown-SP-6-2","TKPVLKI","HKSSLTR","QTTHLSR","Right-ZFN-unknown-SP-6-2","RTDRLIR","QSTTLKR","QRNNLGR")) | |
| --- | --- | --- | --- | --- | --- | --- | --- | --- | --- | --- | --- | --- | --- | --- | --- | --- | --- | --- | --- | --- | --- | --- | --- | --- | --- | --- | --- | --- | --- | --- | --- | --- | --- | --- | --- | --- | --- | --- | --- |
| [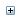](javascript:TreeView_ToggleNode(ctl00_ContentPlaceHolder1_tree12_Data,6,document.getElementById('ctl00_ContentPlaceHolder1_tree12n6'),'%20',document.getElementById('ctl00_ContentPlaceHolder1_tree12n6Nodes'))) | | ZFN-unknown-SP-6-3 1744 cAACTCCCCCTCAGAA[GCAGGAGCC](http://bindr.gdcb.iastate.edu:8080/ZiFDB/controller/searchArray?site=GCCGGAGCA)g 1769  1744 g[TTGAGGGGG](http://bindr.gdcb.iastate.edu:8080/ZiFDB/controller/searchArray?site=GTTGGAGGG)AGTCTTCGTCCTCGGc 1769 | |  |

|  | 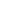 | | | FINGER | HELIX | TRIPLET | REFERENCE NUMBER | SOURCE | | --- | --- | --- | --- | --- | | Left F1 | TTALLKR | [GTT](http://bindr.gdcb.iastate.edu:8080/ZiFDB/controller/searchFinger?target=GTT) | - | CoDA | | Left F2 | QSAHLKR | [GGA](http://bindr.gdcb.iastate.edu:8080/ZiFDB/controller/searchFinger?target=GGA) | - | CoDA | | Left F3 | RTEHLAR | [GGG](http://bindr.gdcb.iastate.edu:8080/ZiFDB/controller/searchFinger?target=GGG) | - | CoDA | | Right F1 | DSPTLRR | [GCC](http://bindr.gdcb.iastate.edu:8080/ZiFDB/controller/searchFinger?target=GCC) | - | CoDA | | Right F2 | QSAHLKR | [GGA](http://bindr.gdcb.iastate.edu:8080/ZiFDB/controller/searchFinger?target=GGA) | - | CoDA | | Right F3 | QDVSLVR | [GCA](http://bindr.gdcb.iastate.edu:8080/ZiFDB/controller/searchFinger?target=GCA) | - | CoDA |   [ZF DNA Sequence](javascript:CoDAPopupNucleaseWindow("Left-ZFN-unknown-SP-6-3","TTALLKR","QSAHLKR","RTEHLAR","Right-ZFN-unknown-SP-6-3","DSPTLRR","QSAHLKR","QDVSLVR")) | |
| --- | --- | --- | --- | --- | --- | --- | --- | --- | --- | --- | --- | --- | --- | --- | --- | --- | --- | --- | --- | --- | --- | --- | --- | --- | --- | --- | --- | --- | --- | --- | --- | --- | --- | --- | --- | --- | --- | --- | --- |
| [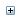](javascript:TreeView_ToggleNode(ctl00_ContentPlaceHolder1_tree12_Data,8,document.getElementById('ctl00_ContentPlaceHolder1_tree12n8'),'%20',document.getElementById('ctl00_ContentPlaceHolder1_tree12n8Nodes'))) | | ZFN-unknown-SP-7-2 1825 cCTCGTCACAATAAAGA[TAGGGGGGC](http://bindr.gdcb.iastate.edu:8080/ZiFDB/controller/searchArray?site=GGCGGGTAG)a 1851  1825 g[GAGCAGTGT](http://bindr.gdcb.iastate.edu:8080/ZiFDB/controller/searchArray?site=GAGGACTGT)TATTTCTATCCCCCCGt 1851 | |  |

|  | 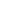 | | | FINGER | HELIX | TRIPLET | REFERENCE NUMBER | SOURCE | | --- | --- | --- | --- | --- | | Left F1 | RTHNLTR | [GAG](http://bindr.gdcb.iastate.edu:8080/ZiFDB/controller/searchFinger?target=GAG) | - | CoDA | | Left F2 | DRGNLTR | [GAC](http://bindr.gdcb.iastate.edu:8080/ZiFDB/controller/searchFinger?target=GAC) | - | CoDA | | Left F3 | QPHGLRA | [TGT](http://bindr.gdcb.iastate.edu:8080/ZiFDB/controller/searchFinger?target=TGT) | - | CoDA | | Right F1 | TNSKLTR | [GGC](http://bindr.gdcb.iastate.edu:8080/ZiFDB/controller/searchFinger?target=GGC) | - | CoDA | | Right F2 | RREHLVR | [GGG](http://bindr.gdcb.iastate.edu:8080/ZiFDB/controller/searchFinger?target=GGG) | - | CoDA | | Right F3 | RPESLAP | [TAG](http://bindr.gdcb.iastate.edu:8080/ZiFDB/controller/searchFinger?target=TAG) | - | CoDA |   [ZF DNA Sequence](javascript:CoDAPopupNucleaseWindow("Left-ZFN-unknown-SP-7-2","RTHNLTR","DRGNLTR","QPHGLRA","Right-ZFN-unknown-SP-7-2","TNSKLTR","RREHLVR","RPESLAP")) | |
| --- | --- | --- | --- | --- | --- | --- | --- | --- | --- | --- | --- | --- | --- | --- | --- | --- | --- | --- | --- | --- | --- | --- | --- | --- | --- | --- | --- | --- | --- | --- | --- | --- | --- | --- | --- | --- | --- | --- | --- |
| [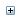](javascript:TreeView_ToggleNode(ctl00_ContentPlaceHolder1_tree12_Data,10,document.getElementById('ctl00_ContentPlaceHolder1_tree12n10'),'%20',document.getElementById('ctl00_ContentPlaceHolder1_tree12n10Nodes'))) | | ZFN-unknown-SP-6-4 2711 aGACAACATCTGTTGA[GGTGGGGAC](http://bindr.gdcb.iastate.edu:8080/ZiFDB/controller/searchArray?site=GACGGGGGT)t 2736  2711 t[CTGTTGTAG](http://bindr.gdcb.iastate.edu:8080/ZiFDB/controller/searchArray?site=GTCGTTGAT)ACAACTCCACCCCTGa 2736 | |  |

|  | 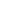 | | | FINGER | HELIX | TRIPLET | REFERENCE NUMBER | SOURCE | | --- | --- | --- | --- | --- | | Left F1 | TKKILTV | [GTC](http://bindr.gdcb.iastate.edu:8080/ZiFDB/controller/searchFinger?target=GTC) | - | CoDA | | Left F2 | HKSSLTR | [GTT](http://bindr.gdcb.iastate.edu:8080/ZiFDB/controller/searchFinger?target=GTT) | - | CoDA | | Left F3 | ISHNLAR | [GAT](http://bindr.gdcb.iastate.edu:8080/ZiFDB/controller/searchFinger?target=GAT) | - | CoDA | | Right F1 | EEANLRR | [GAC](http://bindr.gdcb.iastate.edu:8080/ZiFDB/controller/searchFinger?target=GAC) | - | CoDA | | Right F2 | RREHLVR | [GGG](http://bindr.gdcb.iastate.edu:8080/ZiFDB/controller/searchFinger?target=GGG) | - | CoDA | | Right F3 | VDHHLRR | [GGT](http://bindr.gdcb.iastate.edu:8080/ZiFDB/controller/searchFinger?target=GGT) | - | CoDA |   [ZF DNA Sequence](javascript:CoDAPopupNucleaseWindow("Left-ZFN-unknown-SP-6-4","TKKILTV","HKSSLTR","ISHNLAR","Right-ZFN-unknown-SP-6-4","EEANLRR","RREHLVR","VDHHLRR")) | |
| --- | --- | --- | --- | --- | --- | --- | --- | --- | --- | --- | --- | --- | --- | --- | --- | --- | --- | --- | --- | --- | --- | --- | --- | --- | --- | --- | --- | --- | --- | --- | --- | --- | --- | --- | --- | --- | --- | --- | --- |
| [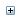](javascript:TreeView_ToggleNode(ctl00_ContentPlaceHolder1_tree12_Data,12,document.getElementById('ctl00_ContentPlaceHolder1_tree12n12'),'%20',document.getElementById('ctl00_ContentPlaceHolder1_tree12n12Nodes'))) | | ZFN-unknown-SP-6-5 3507 aCACAACAAATCAGAA[GACTGAGTT](http://bindr.gdcb.iastate.edu:8080/ZiFDB/controller/searchArray?site=GTTTGAGAC)a 3532  3507 t[GTGTTGTTT](http://bindr.gdcb.iastate.edu:8080/ZiFDB/controller/searchArray?site=GTGGTTTTT)AGTCTTCTGACTCAAt 3532 | |  |

|  | 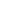 | | | FINGER | HELIX | TRIPLET | REFERENCE NUMBER | SOURCE | | --- | --- | --- | --- | --- | | Left F1 | RNFILQR | [GTG](http://bindr.gdcb.iastate.edu:8080/ZiFDB/controller/searchFinger?target=GTG) | - | CoDA | | Left F2 | HKSSLTR | [GTT](http://bindr.gdcb.iastate.edu:8080/ZiFDB/controller/searchFinger?target=GTT) | - | CoDA | | Left F3 | QRNALSG | [TTT](http://bindr.gdcb.iastate.edu:8080/ZiFDB/controller/searchFinger?target=TTT) | - | CoDA | | Right F1 | TNSVLGR | [GTT](http://bindr.gdcb.iastate.edu:8080/ZiFDB/controller/searchFinger?target=GTT) | - | CoDA | | Right F2 | QREHLTT | [TGA](http://bindr.gdcb.iastate.edu:8080/ZiFDB/controller/searchFinger?target=TGA) | - | CoDA | | Right F3 | DPSNLRR | [GAC](http://bindr.gdcb.iastate.edu:8080/ZiFDB/controller/searchFinger?target=GAC) | - | CoDA |   [ZF DNA Sequence](javascript:CoDAPopupNucleaseWindow("Left-ZFN-unknown-SP-6-5","RNFILQR","HKSSLTR","QRNALSG","Right-ZFN-unknown-SP-6-5","TNSVLGR","QREHLTT","DPSNLRR")) | |
| --- | --- | --- | --- | --- | --- | --- | --- | --- | --- | --- | --- | --- | --- | --- | --- | --- | --- | --- | --- | --- | --- | --- | --- | --- | --- | --- | --- | --- | --- | --- | --- | --- | --- | --- | --- | --- | --- | --- | --- |
| [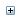](javascript:TreeView_ToggleNode(ctl00_ContentPlaceHolder1_tree12_Data,14,document.getElementById('ctl00_ContentPlaceHolder1_tree12n14'),'%20',document.getElementById('ctl00_ContentPlaceHolder1_tree12n14Nodes'))) | | ZFN-unknown-SP-6-6 4041 tTCCAGCAGAAACAGG[GCAGGAAAC](http://bindr.gdcb.iastate.edu:8080/ZiFDB/controller/searchArray?site=AACGGAGCA)a 4066  4041 a[AGGTCGTCT](http://bindr.gdcb.iastate.edu:8080/ZiFDB/controller/searchArray?site=GGAGCTTCT)TTGTCCCGTCCTTTGt 4066 | |  |

|  | 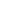 | | | FINGER | HELIX | TRIPLET | REFERENCE NUMBER | SOURCE | | --- | --- | --- | --- | --- | | Left F1 | RPAKLVL | [GGA](http://bindr.gdcb.iastate.edu:8080/ZiFDB/controller/searchFinger?target=GGA) | - | CoDA | | Left F2 | QRSDLTR | [GCT](http://bindr.gdcb.iastate.edu:8080/ZiFDB/controller/searchFinger?target=GCT) | - | CoDA | | Left F3 | QRNTLKG | [TCT](http://bindr.gdcb.iastate.edu:8080/ZiFDB/controller/searchFinger?target=TCT) | - | CoDA | | Right F1 | GGTALVM | [AAC](http://bindr.gdcb.iastate.edu:8080/ZiFDB/controller/searchFinger?target=AAC) | - | CoDA | | Right F2 | QSAHLKR | [GGA](http://bindr.gdcb.iastate.edu:8080/ZiFDB/controller/searchFinger?target=GGA) | - | CoDA | | Right F3 | QDVSLVR | [GCA](http://bindr.gdcb.iastate.edu:8080/ZiFDB/controller/searchFinger?target=GCA) | - | CoDA |   [ZF DNA Sequence](javascript:CoDAPopupNucleaseWindow("Left-ZFN-unknown-SP-6-6","RPAKLVL","QRSDLTR","QRNTLKG","Right-ZFN-unknown-SP-6-6","GGTALVM","QSAHLKR","QDVSLVR")) | |
| --- | --- | --- | --- | --- | --- | --- | --- | --- | --- | --- | --- | --- | --- | --- | --- | --- | --- | --- | --- | --- | --- | --- | --- | --- | --- | --- | --- | --- | --- | --- | --- | --- | --- | --- | --- | --- | --- | --- | --- |
| [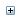](javascript:TreeView_ToggleNode(ctl00_ContentPlaceHolder1_tree12_Data,16,document.getElementById('ctl00_ContentPlaceHolder1_tree12n16'),'%20',document.getElementById('ctl00_ContentPlaceHolder1_tree12n16Nodes'))) | | ZFN-unknown-SP-6-7 6001 aGACCCCAACCCACAA[GAAGTAGTA](http://bindr.gdcb.iastate.edu:8080/ZiFDB/controller/searchArray?site=GTAGTAGAA)t 6026  6001 t[CTGGGGTTG](http://bindr.gdcb.iastate.edu:8080/ZiFDB/controller/searchArray?site=GTCGGGGTT)GGTGTTCTTCATCATa 6026 | |  |

|  | 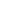 | | | FINGER | HELIX | TRIPLET | REFERENCE NUMBER | SOURCE | | --- | --- | --- | --- | --- | | Left F1 | TKSLLAR | [GTC](http://bindr.gdcb.iastate.edu:8080/ZiFDB/controller/searchFinger?target=GTC) | - | CoDA | | Left F2 | RREHLVR | [GGG](http://bindr.gdcb.iastate.edu:8080/ZiFDB/controller/searchFinger?target=GGG) | - | CoDA | | Left F3 | INHSLRR | [GTT](http://bindr.gdcb.iastate.edu:8080/ZiFDB/controller/searchFinger?target=GTT) | - | CoDA | | Right F1 | QQQALKR | [GTA](http://bindr.gdcb.iastate.edu:8080/ZiFDB/controller/searchFinger?target=GTA) | - | CoDA | | Right F2 | QRSSLVR | [GTA](http://bindr.gdcb.iastate.edu:8080/ZiFDB/controller/searchFinger?target=GTA) | - | CoDA | | Right F3 | QRNNLGR | [GAA](http://bindr.gdcb.iastate.edu:8080/ZiFDB/controller/searchFinger?target=GAA) | - | CoDA |   [ZF DNA Sequence](javascript:CoDAPopupNucleaseWindow("Left-ZFN-unknown-SP-6-7","TKSLLAR","RREHLVR","INHSLRR","Right-ZFN-unknown-SP-6-7","QQQALKR","QRSSLVR","QRNNLGR")) | |
| --- | --- | --- | --- | --- | --- | --- | --- | --- | --- | --- | --- | --- | --- | --- | --- | --- | --- | --- | --- | --- | --- | --- | --- | --- | --- | --- | --- | --- | --- | --- | --- | --- | --- | --- | --- | --- | --- | --- | --- |
| [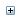](javascript:TreeView_ToggleNode(ctl00_ContentPlaceHolder1_tree12_Data,18,document.getElementById('ctl00_ContentPlaceHolder1_tree12n18'),'%20',document.getElementById('ctl00_ContentPlaceHolder1_tree12n18Nodes'))) | | ZFN-unknown-SP-7-3 7418 cAGCAGCAGAACAATTT[GCTGAGGGC](http://bindr.gdcb.iastate.edu:8080/ZiFDB/controller/searchArray?site=GGCGAGGCT)t 7444  7418 g[TCGTCGTCT](http://bindr.gdcb.iastate.edu:8080/ZiFDB/controller/searchArray?site=GCTGCTTCT)TGTTAAACGACTCCCGa 7444 | |  |

|  | 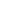 | | | FINGER | HELIX | TRIPLET | REFERENCE NUMBER | SOURCE | | --- | --- | --- | --- | --- | | Left F1 | MKNTLTR | [GCT](http://bindr.gdcb.iastate.edu:8080/ZiFDB/controller/searchFinger?target=GCT) | - | CoDA | | Left F2 | QRSDLTR | [GCT](http://bindr.gdcb.iastate.edu:8080/ZiFDB/controller/searchFinger?target=GCT) | - | CoDA | | Left F3 | QRNTLKG | [TCT](http://bindr.gdcb.iastate.edu:8080/ZiFDB/controller/searchFinger?target=TCT) | - | CoDA | | Right F1 | SPSKLVR | [GGC](http://bindr.gdcb.iastate.edu:8080/ZiFDB/controller/searchFinger?target=GGC) | - | CoDA | | Right F2 | RQDNLGR | [GAG](http://bindr.gdcb.iastate.edu:8080/ZiFDB/controller/searchFinger?target=GAG) | - | CoDA | | Right F3 | VSNTLTR | [GCT](http://bindr.gdcb.iastate.edu:8080/ZiFDB/controller/searchFinger?target=GCT) | - | CoDA |   [ZF DNA Sequence](javascript:CoDAPopupNucleaseWindow("Left-ZFN-unknown-SP-7-3","MKNTLTR","QRSDLTR","QRNTLKG","Right-ZFN-unknown-SP-7-3","SPSKLVR","RQDNLGR","VSNTLTR")) | |
| --- | --- | --- | --- | --- | --- | --- | --- | --- | --- | --- | --- | --- | --- | --- | --- | --- | --- | --- | --- | --- | --- | --- | --- | --- | --- | --- | --- | --- | --- | --- | --- | --- | --- | --- | --- | --- | --- | --- | --- |
| [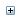](javascript:TreeView_ToggleNode(ctl00_ContentPlaceHolder1_tree12_Data,20,document.getElementById('ctl00_ContentPlaceHolder1_tree12n20'),'%20',document.getElementById('ctl00_ContentPlaceHolder1_tree12n20Nodes'))) | | ZFN-unknown-SP-6-8 8162 aATCTCCTACAGTATT[GGAGTCAGG](http://bindr.gdcb.iastate.edu:8080/ZiFDB/controller/searchArray?site=AGGGTCGGA)a 8187  8162 t[TAGAGGATG](http://bindr.gdcb.iastate.edu:8080/ZiFDB/controller/searchArray?site=GATGGAGTA)TCATAACCTCAGTCCt 8187 | |  |

|  | 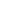 | | | FINGER | HELIX | TRIPLET | REFERENCE NUMBER | SOURCE | | --- | --- | --- | --- | --- | | Left F1 | TRQRLRI | [GAT](http://bindr.gdcb.iastate.edu:8080/ZiFDB/controller/searchFinger?target=GAT) | - | CoDA | | Left F2 | QSAHLKR | [GGA](http://bindr.gdcb.iastate.edu:8080/ZiFDB/controller/searchFinger?target=GGA) | - | CoDA | | Left F3 | QSTSLQR | [GTA](http://bindr.gdcb.iastate.edu:8080/ZiFDB/controller/searchFinger?target=GTA) | - | CoDA | | Right F1 | RRTHLRV | [AGG](http://bindr.gdcb.iastate.edu:8080/ZiFDB/controller/searchFinger?target=AGG) | - | CoDA | | Right F2 | DHSSLKR | [GTC](http://bindr.gdcb.iastate.edu:8080/ZiFDB/controller/searchFinger?target=GTC) | - | CoDA | | Right F3 | QTTHLSR | [GGA](http://bindr.gdcb.iastate.edu:8080/ZiFDB/controller/searchFinger?target=GGA) | - | CoDA |   [ZF DNA Sequence](javascript:CoDAPopupNucleaseWindow("Left-ZFN-unknown-SP-6-8","TRQRLRI","QSAHLKR","QSTSLQR","Right-ZFN-unknown-SP-6-8","RRTHLRV","DHSSLKR","QTTHLSR")) | |
| --- | --- | --- | --- | --- | --- | --- | --- | --- | --- | --- | --- | --- | --- | --- | --- | --- | --- | --- | --- | --- | --- | --- | --- | --- | --- | --- | --- | --- | --- | --- | --- | --- | --- | --- | --- | --- | --- | --- | --- |
| [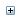](javascript:TreeView_ToggleNode(ctl00_ContentPlaceHolder1_tree12_Data,22,document.getElementById('ctl00_ContentPlaceHolder1_tree12n22'),'%20',document.getElementById('ctl00_ContentPlaceHolder1_tree12n22Nodes'))) | | ZFN-unknown-SP-6-9 8294 cACATACCTAGAAGAA[TAAGACAGG](http://bindr.gdcb.iastate.edu:8080/ZiFDB/controller/searchArray?site=AGGGACTAA)g 8319  8294 g[TGTATGGAT](http://bindr.gdcb.iastate.edu:8080/ZiFDB/controller/searchArray?site=TGTGTATAG)CTTCTTATTCTGTCCc 8319 | |  |

|  | 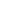 | | | FINGER | HELIX | TRIPLET | REFERENCE NUMBER | SOURCE | | --- | --- | --- | --- | --- | | Left F1 | RKQHLQL | [TGT](http://bindr.gdcb.iastate.edu:8080/ZiFDB/controller/searchFinger?target=TGT) | - | CoDA | | Left F2 | QRSSLVR | [GTA](http://bindr.gdcb.iastate.edu:8080/ZiFDB/controller/searchFinger?target=GTA) | - | CoDA | | Left F3 | RLDGLAG | [TAG](http://bindr.gdcb.iastate.edu:8080/ZiFDB/controller/searchFinger?target=TAG) | - | CoDA | | Right F1 | RRAHLLN | [AGG](http://bindr.gdcb.iastate.edu:8080/ZiFDB/controller/searchFinger?target=AGG) | - | CoDA | | Right F2 | DRGNLTR | [GAC](http://bindr.gdcb.iastate.edu:8080/ZiFDB/controller/searchFinger?target=GAC) | - | CoDA | | Right F3 | QSGNLHT | [TAA](http://bindr.gdcb.iastate.edu:8080/ZiFDB/controller/searchFinger?target=TAA) | - | CoDA |   [ZF DNA Sequence](javascript:CoDAPopupNucleaseWindow("Left-ZFN-unknown-SP-6-9","RKQHLQL","QRSSLVR","RLDGLAG","Right-ZFN-unknown-SP-6-9","RRAHLLN","DRGNLTR","QSGNLHT")) | |
| --- | --- | --- | --- | --- | --- | --- | --- | --- | --- | --- | --- | --- | --- | --- | --- | --- | --- | --- | --- | --- | --- | --- | --- | --- | --- | --- | --- | --- | --- | --- | --- | --- | --- | --- | --- | --- | --- | --- | --- |
| [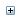](javascript:TreeView_ToggleNode(ctl00_ContentPlaceHolder1_tree12_Data,24,document.getElementById('ctl00_ContentPlaceHolder1_tree12n24'),'%20',document.getElementById('ctl00_ContentPlaceHolder1_tree12n24Nodes'))) | | ZFN-unknown-SP-7-4 8413 aGCCAGCAGCAGATAGG[GTGGGAGCA](http://bindr.gdcb.iastate.edu:8080/ZiFDB/controller/searchArray?site=GCAGGAGTG)g 8439  8413 t[CGGTCGTCG](http://bindr.gdcb.iastate.edu:8080/ZiFDB/controller/searchArray?site=GGCGCTGCT)TCTATCCCACCCTCGTc 8439 | |  |

|  | 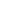 | | | FINGER | HELIX | TRIPLET | REFERENCE NUMBER | SOURCE | | --- | --- | --- | --- | --- | | Left F1 | APSKLAR | [GGC](http://bindr.gdcb.iastate.edu:8080/ZiFDB/controller/searchFinger?target=GGC) | - | CoDA | | Left F2 | QRSDLTR | [GCT](http://bindr.gdcb.iastate.edu:8080/ZiFDB/controller/searchFinger?target=GCT) | - | CoDA | | Left F3 | LRASLRR | [GCT](http://bindr.gdcb.iastate.edu:8080/ZiFDB/controller/searchFinger?target=GCT) | - | CoDA | | Right F1 | KNTRLSV | [GCA](http://bindr.gdcb.iastate.edu:8080/ZiFDB/controller/searchFinger?target=GCA) | - | CoDA | | Right F2 | QSAHLKR | [GGA](http://bindr.gdcb.iastate.edu:8080/ZiFDB/controller/searchFinger?target=GGA) | - | CoDA | | Right F3 | RNTALQH | [GTG](http://bindr.gdcb.iastate.edu:8080/ZiFDB/controller/searchFinger?target=GTG) | - | CoDA |   [ZF DNA Sequence](javascript:CoDAPopupNucleaseWindow("Left-ZFN-unknown-SP-7-4","APSKLAR","QRSDLTR","LRASLRR","Right-ZFN-unknown-SP-7-4","KNTRLSV","QSAHLKR","RNTALQH")) | |
| --- | --- | --- | --- | --- | --- | --- | --- | --- | --- | --- | --- | --- | --- | --- | --- | --- | --- | --- | --- | --- | --- | --- | --- | --- | --- | --- | --- | --- | --- | --- | --- | --- | --- | --- | --- | --- | --- | --- | --- |
| [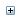](javascript:TreeView_ToggleNode(ctl00_ContentPlaceHolder1_tree12_Data,26,document.getElementById('ctl00_ContentPlaceHolder1_tree12n26'),'%20',document.getElementById('ctl00_ContentPlaceHolder1_tree12n26Nodes'))) | | ZFN-unknown-SP-7-5 8416 cAGCAGCAGATAGGGTG[GGAGCAGCA](http://bindr.gdcb.iastate.edu:8080/ZiFDB/controller/searchArray?site=GCAGCAGGA)t 8442  8416 g[TCGTCGTCT](http://bindr.gdcb.iastate.edu:8080/ZiFDB/controller/searchArray?site=GCTGCTTCT)ATCCCACCCTCGTCGTa 8442 | |  |

|  | 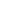 | | | FINGER | HELIX | TRIPLET | REFERENCE NUMBER | SOURCE | | --- | --- | --- | --- | --- | | Left F1 | MKNTLTR | [GCT](http://bindr.gdcb.iastate.edu:8080/ZiFDB/controller/searchFinger?target=GCT) | - | CoDA | | Left F2 | QRSDLTR | [GCT](http://bindr.gdcb.iastate.edu:8080/ZiFDB/controller/searchFinger?target=GCT) | - | CoDA | | Left F3 | QRNTLKG | [TCT](http://bindr.gdcb.iastate.edu:8080/ZiFDB/controller/searchFinger?target=TCT) | - | CoDA | | Right F1 | QRGTLNR | [GCA](http://bindr.gdcb.iastate.edu:8080/ZiFDB/controller/searchFinger?target=GCA) | - | CoDA | | Right F2 | QSTTLKR | [GCA](http://bindr.gdcb.iastate.edu:8080/ZiFDB/controller/searchFinger?target=GCA) | - | CoDA | | Right F3 | QKPHLSR | [GGA](http://bindr.gdcb.iastate.edu:8080/ZiFDB/controller/searchFinger?target=GGA) | - | CoDA |   [ZF DNA Sequence](javascript:CoDAPopupNucleaseWindow("Left-ZFN-unknown-SP-7-5","MKNTLTR","QRSDLTR","QRNTLKG","Right-ZFN-unknown-SP-7-5","QRGTLNR","QSTTLKR","QKPHLSR")) | |
| --- | --- | --- | --- | --- | --- | --- | --- | --- | --- | --- | --- | --- | --- | --- | --- | --- | --- | --- | --- | --- | --- | --- | --- | --- | --- | --- | --- | --- | --- | --- | --- | --- | --- | --- | --- | --- | --- | --- | --- |
| [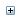](javascript:TreeView_ToggleNode(ctl00_ContentPlaceHolder1_tree12_Data,28,document.getElementById('ctl00_ContentPlaceHolder1_tree12n28'),'%20',document.getElementById('ctl00_ContentPlaceHolder1_tree12n28Nodes'))) | | | [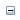](javascript:TreeView_ToggleNode(ctl00_ContentPlaceHolder1_tree12_Data,28,document.getElementById('ctl00_ContentPlaceHolder1_tree12n28'),'%20',document.getElementById('ctl00_ContentPlaceHolder1_tree12n28Nodes'))) | ZFN-unknown-SP-7-6 8482 aTACAGCAGCTACCAAT[GCTGCTTGT](http://bindr.gdcb.iastate.edu:8080/ZiFDB/controller/searchArray?site=TGTGCTGCT)g 8508  8482 t[ATGTCGTCG](http://bindr.gdcb.iastate.edu:8080/ZiFDB/controller/searchArray?site=GTAGCTGCT)ATGGTTACGACGAACAc 8508 | | --- | --- |  |  | 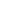 | | FINGER | HELIX | TRIPLET | REFERENCE NUMBER | SOURCE | | --- | --- | --- | --- | --- | | Left F1 | QQSSLLR | [GTA](http://bindr.gdcb.iastate.edu:8080/ZiFDB/controller/searchFinger?target=GTA) | - | CoDA | | Left F2 | QRSDLTR | [GCT](http://bindr.gdcb.iastate.edu:8080/ZiFDB/controller/searchFinger?target=GCT) | - | CoDA | | Left F3 | LRASLRR | [GCT](http://bindr.gdcb.iastate.edu:8080/ZiFDB/controller/searchFinger?target=GCT) | - | CoDA | | RightF1 | KRQHLEY | [TGT](http://bindr.gdcb.iastate.edu:8080/ZiFDB/controller/searchFinger?target=TGT) | - | CoDA | | RightF2 | QRSDLTR | [GCT](http://bindr.gdcb.iastate.edu:8080/ZiFDB/controller/searchFinger?target=GCT) | - | CoDA | | RightF3 | LRASLRR | [GCT](http://bindr.gdcb.iastate.edu:8080/ZiFDB/controller/searchFinger?target=GCT) | - | CoDA |   [ZF DNA Sequence](javascript:CoDAPopupNucleaseWindow("Left-ZFN-unknown-SP-7-6","QQSSLLR","QRSDLTR","LRASLRR","Right-ZFN-unknown-SP-7-6","KRQHLEY","QRSDLTR","LRASLRR")) | | --- | --- | --- | --- | --- | --- | --- | --- | --- | --- | --- | --- | --- | --- | --- | --- | --- | --- | --- | --- | --- | --- | --- | --- | --- | --- | --- | --- | --- | --- | --- | --- | --- | --- | --- | --- | --- | --- | | |  |
